# Antisense oligonucleotide therapeutic approach for Timothy syndrome

**DOI:** 10.1038/s41586-024-07310-6

**Published:** 2024-04-24

**Authors:** Xiaoyu Chen, Fikri Birey, Min-Yin Li, Omer Revah, Rebecca Levy, Mayuri Vijay Thete, Noah Reis, Konstantin Kaganovsky, Massimo Onesto, Noriaki Sakai, Zuzana Hudacova, Jin Hao, Xiangling Meng, Seiji Nishino, John Huguenard, Sergiu P. Pașca

**Affiliations:** 1 Department of Psychiatry and Behavioral Sciences, Stanford University, Stanford, CA USA; 2 Stanford Brain Organogenesis, Wu Tsai Neurosciences Institute & Bio-X, Stanford University, Stanford, CA USA; 3 Department of Neurology, Division of Child Neurology, Stanford University, Stanford, CA USA; 4 Department of Neurology and Neurological Sciences, Stanford University, Stanford, CA USA; 5 Present Address: Department of Human Genetics, Emory University, Atlanta, GA USA

**Keywords:** Autism spectrum disorders, Development of the nervous system

## Abstract

Timothy syndrome (TS) is a severe, multisystem disorder characterized by autism, epilepsy, long-QT syndrome and other neuropsychiatric conditions^1^. TS type 1 (TS1) is caused by a gain-of-function variant in the alternatively spliced and developmentally enriched *CACNA1C* exon 8A, as opposed to its counterpart exon 8. We previously uncovered several phenotypes in neurons derived from patients with TS1, including delayed channel inactivation, prolonged depolarization-induced calcium rise, impaired interneuron migration, activity-dependent dendrite retraction and an unanticipated persistent expression of exon 8A^2–6^. We reasoned that switching *CACNA1C* exon utilization from 8A to 8 would represent a potential therapeutic strategy. Here we developed antisense oligonucleotides (ASOs) to effectively decrease the inclusion of exon 8A in human cells both in vitro and, following transplantation, in vivo. We discovered that the ASO-mediated switch from exon 8A to 8 robustly rescued defects in patient-derived cortical organoids and migration in forebrain assembloids. Leveraging a transplantation platform previously developed^7^, we found that a single intrathecal ASO administration rescued calcium changes and in vivo dendrite retraction of patient neurons, suggesting that suppression of *CACNA1C* exon 8A expression is a potential treatment for TS1. Broadly, these experiments illustrate how a multilevel, in vivo and in vitro stem cell model-based approach can identify strategies to reverse disease-relevant neural pathophysiology.

## Abstract (web summary)

Antisense oligonucleotides effectively decrease the inclusion of exon  8A of *CACNA1C* in human cells both in vitro and in rodents transplanted with human brain organoids, and a single intrathecal administration rescued both calcium changes and in vivo dendrite morphology of patient neurons.

## Main

Timothy syndrome type 1 (TS1 or TS) is a severe genetic disorder with significant morbidity and mortality^8–11^ caused by the heterozygous c.1216G>A pathogenic variant in exon 8A of *CACNA1C*, resulting in a p.G406R missense variant in the α1 subunit of the L-type voltage-gated calcium channel Ca_V_1.2 (ref. ^8^). Ca_V_1.2 is broadly expressed in both the developing and adult nervous system, primarily in neurons but also in some progenitors and glial cells^12,13^. TS1 affects multiple organ systems and is one of the most penetrant genetic aetiologies of autism spectrum disorder and epilepsy^8^. Common variants in *CACNA1C* have also been strongly associated with other neuropsychiatric disorders including schizophrenia, bipolar disorder and attention deficit hyperactivity disorder^9,11^, suggesting that Ca_V_1.2 is a key susceptibility factor for neuropsychiatric conditions.

Studies in human-induced pluripotent stem (hiPS) cell-derived cardiomyocytes and neurons in both two- and three-dimensional systems reported that cells derived from individuals with TS1 showed delayed voltage-dependent channel inactivation and increased depolarization-induced calcium entry^2,4,14^, leading to abnormal excitability. Moreover, using human forebrain assembloids (hFA) generated by the integration of human cortical organoids (hCO) and human subpallial organoids (hSO), we previously described defects in cortical interneuron migration: TS1 interneurons undergo more frequent nucleokinetic saltations driven by enhanced GABA sensitivity but saltation length is reduced due to aberrant cytoskeletal function, leading to overall defective migration^3,4^.

Surprisingly, TS1-derived neurons have an abnormally high level of the *CACNA1C* splice form containing exon 8A compared with control neurons^4,6^. Moreover, splicing of *CACNA1C* is developmentally regulated in both mouse and human, with a shift in exon utilization from exon 8A to 8 during early development^15^. Inclusion of either of these mutually exclusive spliced exons has been shown to yield channel isoforms with relatively similar electrophysiological features^16,17^. These findings raise the possibility that decreasing inclusion of the 8A isoform of *CACNA1C* may function as a therapeutic strategy for TS1.

In this study we developed an antisense oligonucleotide (ASO)-based intervention to effectively decrease exon 8A inclusion in neural cells derived from three individuals with TS and an isogenic G406R hiPS cell line. ASOs are short oligonucleotides that can bind to target RNAs, activate cytoplasmic degradation of target RNAs or modulate splicing of pre-messenger RNAs inside the nucleus^18,19^. Several ASOs targeting splicing have advanced from the bench to the clinic as therapeutic options, including for spinal muscular atrophy^20–23^ and Duchenne muscular dystrophy^24^. Here we first demonstrated that the TS1 p.G406R mutation directly enhanced splicing of the mutated exon 8A. We then performed a screen to identify ASOs that can robustly inhibit splicing of exon 8A, in a time- and dose-dependent manner. Direct application of these ASOs to human cortical neurons in either two- or three-dimensional cultures derived from individuals with TS rescued both delayed channel inactivation and the defect in depolarization-induced calcium elevation. Moreover, these ASOs restored previously identified cortical interneuron migration defects in TS1 forebrain assembloids. Lastly, to verify ASO effectiveness in an in vivo setting, we leveraged a transplantation model that we have recently developed^7^. In this system, human stem cell-derived cortical organoids transplanted (t-hCO) into the somatosensory cortex of newborn athymic rats grow and develop mature cell types that integrate into sensory and motivation-related circuits. We discovered that intrathecal injection of an ASO into rats transplanted with human TS1 cortical organoids resulted in a robust downregulation of exon 8A, accompanied by rescue of both depolarization-induced calcium defects and aberrant activity-dependent dendritic morphology. Taken together, these experiments demonstrate a new genetic rescue strategy for a devastating neurodevelopmental disorder.

## Enhanced inclusion of *CACNA1C* exon 8A results in abnormal channel function in human cortical neurons

**Fig. 1:**
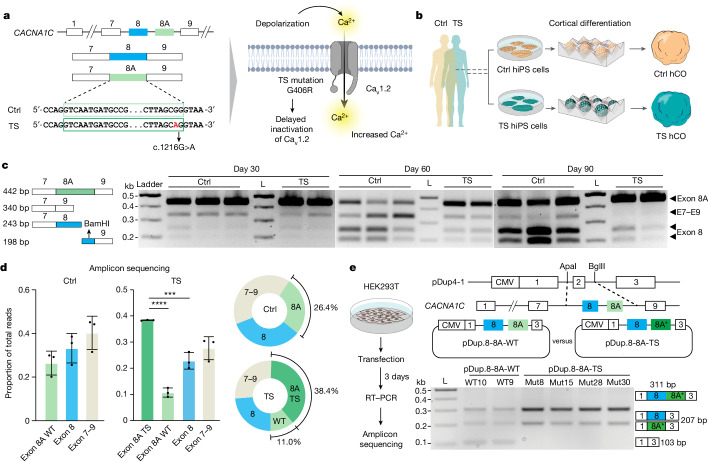
The TS G406R variant enhances inclusion of *CACNA1C* exon 8A in human neurons. **a**, Schematics illustrating the TS pathogenic variant in the alternatively spliced exon 8A (left) and the resulting gain-of-function channel variant (right). The heterozygous G>A variant (black arrow) is located towards the 3′ end of exon 8A. **b**, Generation of hCO from control (Ctrl) and TS hiPS cells. **c**, Schematic of the RFLP assay. Left, PCR products amplified from hCO cDNA; the exon 8-containing amplicon is recognized by restriction enzyme BamHI; exon 8A, 8 and 7–9 amplicons have different molecular weights on agarose gel. Right, RFLP gel image of control and TS hCO at days 30, 60 and 90 of differentiation. Each column represents a hCO derived from different hiPS cell lines. L, ladder. **d**, Next-generation sequencing of amplicons generated from day 60 hCO. Left, PCR products were obtained using a forward primer targeting exon 7 and a reverse primer targeting exon 9; both primers have an Illumina adaptor at their 5′. Right, proportions of exon 8A WT, exon 8A TS, exon 8 and exons 7–9 are shown (*n* = 3 for WT hCO, *n* = 3 for TS hCO). Data presented as mean ± s.d. One-way analysis of variance (ANOVA) with Tukey’s post hoc test: for control hCO, *F*_2,6_ = 3.246, *P* = 0.1108; for TS hCO, *F*_3,8_ = 50.28, *P* < 0.0001. ****P < 0.0001, ***P < 0.001, **P < 0.01. **e**, Generation of minigene splicing reporters for exons 8 and 8A of *CACNA1C*. Left, experimental strategy for testing minisplicing reporters in HEK293T cells. Right, a *CACNA1C* DNA fragment (isolated from TS hiPS cells) was inserted into a pDup4-1backbone resulting in two vectors, pDup8-8A-WT and pDup8-8A-TS. bp, base pairs. Source Data

**Extended Data Fig. 1:**
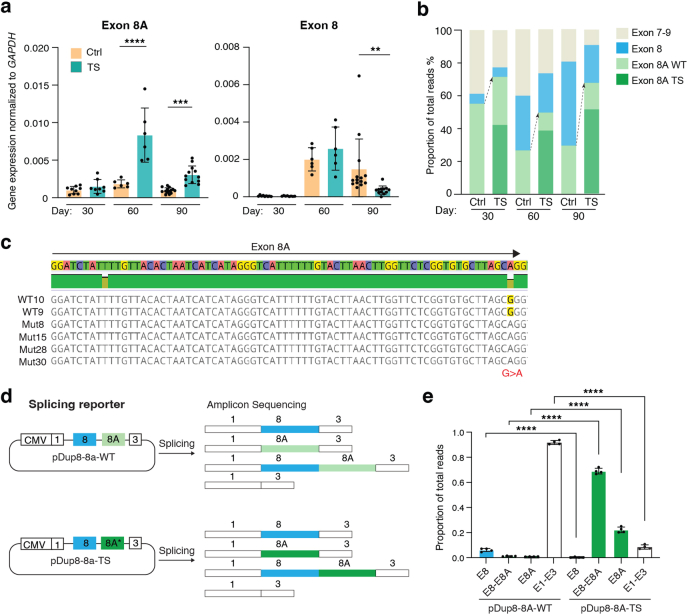
The TS variant causes abnormal splicing of *CACNA1C* in human neurons in vitro*.* **a**. RT-qPCR analysis of the exon 8A (left) and exon 8 (right) of *CACNA1C* during hCO differentiation. Data are shown as mean ± s.d. Dots represent individual samples collected from different differentiations and different hiPS cell lines (day 30: n = 9 Ctrl and n = 8 TS; day 60: n = 3 Ctrl and n = 6 TS; day 90: n = 14 Ctrl and n = 12 TS). One-way ANOVA with Bonferroni test: exon 8A, F_5, 49_ = 29.05, P < 0.0001, TS vs Ctrl, day 30, P > 0.9999; day 60, ****P < 0.0001; day 90, ***P = 0.0009. exon 8, F_5, 49_ = 10.32,P < 0.0001, TS vs. Ctrl, day 30, P > 0.9999; day 60, P = 0.6449; day 90, **P = 0.0098. **b**. Next-generation sequencing of the amplicons generated from day 60 hCO. The PCR products were obtained using a forward primer targeting exon 7 and a reverse primer targeting exon 9 (both primers have an Illumina adapter at their 5’). **c**. Sequencing validation of the minigene splicing reporter vectors. The c.1216 G > A TS variant is annotated in red. **d**. Schematic illustrating possible splicing outcomes from splicing reporters pDup8-8a^WT^ and. pDup8-8a^TS^. **e**. Sequencing of the amplicons generated from the cDNA of transfected HEK293T cells (n = 4; Data are presented as mean ± s.d. One-way ANOVA with Tukey’s post hoc test: F_7,24_ = 2295, P < 0.0001, ****P < 0.0001). Source Data

**Extended Data Fig. 2:**
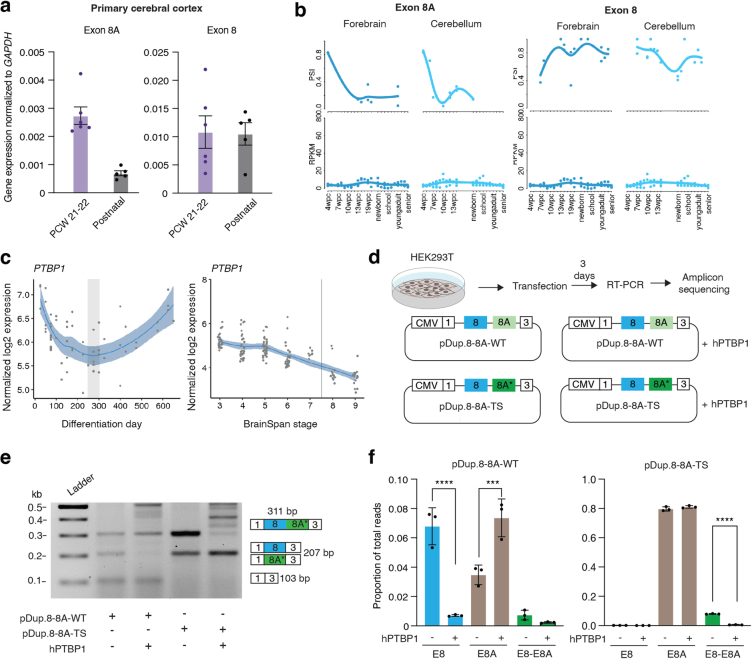
*CACNA1C* splicing and the role of PTBP1. **a**. qPCR of exon 8A and exon 8 in human cerebral cortex at postconceptional week 21 and 22 (n = 2 samples), and postnatal frontal cerebral cortex at age of 3 and 18 years (n = 2 samples). Data are presented as mean ± s.e.m. Each dot represents a piece of tissue individually processed for RNA. Statistical analysis was not performed. **b**. Exon 8A (hum.12879.s20) and exon 8 (hum.12879.s18) splicing patterns across developmental stages in human forebrain and cerebellum. Data from Evo-devo Alternative Splicing: https://apps.kaessmannlab.org/alternative-splicing (ref. ^25^). **c**. PTBP1 gene expression during differentiation of hCO (left) and in BrainSpan. Data plotted from: http://solo.bmap.ucla.edu/shiny/GECO/. The vertical bar indicates birth. Shaded areas represent 95% confidence intervals and vertical gray areas denote the prenatal to postnatal transition (ref. ^26^). **d**. Experimental strategy to explore the role of PTBP1 in exon 8/8A splicing. pDup8-8A-WT and pDup8-8A-TS were transfected either with or without the PTBP1-encoding plasmid. RNA was extracted at 3 days post transfection and cDNA was amplified and loaded onto a 2% agarose gel. **e**. Gel image of the PCR product amplifying the cDNA from the transfected mini-splicing reporters and PTBP1. RNA was extracted at 3 days post-transfection and cDNA was amplified and loaded onto a 2% agarose gel. **f**. Next generation sequencing of the amplicons generated from the cDNA of transfected HEK293T cells (n = 3 independent experiments). Data are presented as mean ± s.d. One-way ANOVA with multiple comparisons between conditions with PTBP1 or without PTBP1. For pDup8-8A-WT, F_5,12_ = 48.56, P < 0.0001, ****P < 0.0001, ***P = 0.0002. For pDup8-8A-TS, F_5,12_ = 10599, P < 0.0001, ****P < 0.0001. Source Data

Exons 8 and 8A are mutually exclusive, 104-nucleotide-long exons of the *CACNA1C* gene (Fig. 1a,b). During cortical differentiation in vitro, in both two-dimensional cultures^2,6^ and three-dimensional hCO (Extended Data Fig. 1a), exon 8A is expressed at higher levels in the early stages but this changes in favour of exon 8 over time (Extended Data Fig. 1a; *P* < 0.001). We also verified this using human primary cortical tissues^25^ (Extended Data Fig. 2a,b). Interestingly, hCO derived from patients with TS1 expressed considerably higher levels of exon 8A compared with control hCO at days 60–90 of differentiation, which is consistent with our previous observations^4,6^. A restriction fragment-length polymorphism (RFLP) assay that uses the BamHI restriction enzyme to selectively cut exon 8 further confirmed this finding (Fig. 1c), raising the possibility that the G406R mutation may directly interfere with splicing and enhance its own inclusion, which could amplify disease phenotypes by prolonging the expression of mutated Ca_V_1.2.

To determine whether the TS mutation directly causes increased levels of exon 8A we first analysed its mRNA composition in TS hCO, which contains both wild-type (WT) and p.G406R exon 8A alleles. Given the heterozygous nature of the mutation in patients with TS1, we reasoned that equal amounts of WT and p.G406R alleles of exon 8A are present in neural cells. However, by sequencing the amplicons spanning exons 7–9 from complementary DNA of both TS and WT hCO, we discovered that the elevated exon 8A expression in TS samples predominantly contained the p.G406R allele (Fig. 1d,e and Extended Data Fig. 1b). We next asked whether this TS-associated enhanced splicing depends on the cellular or genomic context. We generated two minigene splicing reporters in which DNA fragments of around 1 kb spanning exons 8 and 8A (either WT or TS) were inserted into the pDup4-1 reporter backbone (pDup8-8A^WT^ and pDup8-8A^TS^; Fig. 1e and Extended Data Fig. 1c,d). Transfection and amplification of these two vectors in HEK293T cells showed markedly different splicing outcomes (*P* < 0.0001; Fig. 1e and Extended Data Fig. 1d,e). The WT pDup8-8A^WT^ mostly transcribed exon 8 whereas the mutant pDup8-8A^TS^ preferentially transcribed exon 8A, indicating that the TS mutation is sufficient to shift *CACNA1C* splicing in favour of exon 8A, independently of the cellular context. Previous studies found that the splicing master regulator Ptbp1 modulates exon 8 versus 8A splicing of mouse *Cacna1c*^15^. Both organoids and human primary brain RNA sequencing data show that *PTBP1* expression decreases over time^26^ (Extended Data Fig. 2c). To explore the role of PTBP1 in *CACNA1C* splicing, we transfected *CACNA1C* minigene splicing reporters in the presence or absence of human PTBP1 (Extended Data Fig. 2c,d). We found marked changes in splicing patterns following the addition of PTBP1 (Extended Data Fig. 2d), and also increased exon 8A-containing transcripts (Extended Data Fig. 2f). Taken together, these experiments demonstrate that the TS exon 8A *CACNA1C* variant directly and persistently enhances its own abundance, potentially by interfering with splicing machinery, and that splicing regulator PTBP1 affects the selection of exon 8 versus 8A.

## Screening of ASOs that can reduce exon 8A in favour of exon 8 *CACNA1C* isoforms in human neural cells

**Fig. 2:**
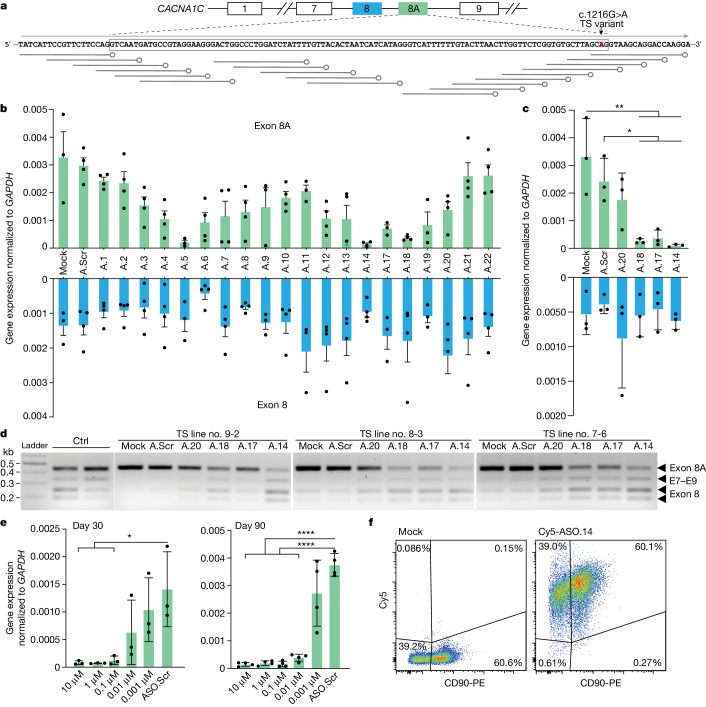
Screening of ASOs that can reduce exon 8A in favour of exon 8 *CACNA1C* isoforms in human neural cells. **a**, ASO design. Arrow denotes the location of the TS variant. **b**, RT–qPCR of exons 8A and 8 in ASO-treated dissociated TS hCO differentiated for 152 days. hCO derived from *n* = 2 TS hiPS cell lines (nos. 9-2 and 8-3) were dissociated and plated. For both TS lines, 10 μM ASO was added to two separated wells resulting in a total of four data points. RNA extraction was carried out 3 days post-exposure. Data are mean + s.e.m. **c**, RT–qPCR analysis of exons 8A and 8 of ASO-treated hCO. Data are mean + s.d. Three TS hiPS cell lines were used (*n* = 3). One-way ANOVA with Tukey’s post hoc test: for exon 8A, *F*_5,12_ = 8.870, *P* = 0.0010, *P < 0.05, **P < 0.01; for exon 8, *F*_5,12_ = 0.6689, *P* = 0.6546. **d**, RFLP analysis from **c**. The size of corresponding amplicons is annotated (black arrowheads). **e**, Serial concentration dilutions of ASO.14 were used to evaluate dose-dependent splicing modulation on *CACNA1C* in hCO. ASO.14 was applied at differentiation day 30 (*n* = 3 individual hCO from three hiPS cell lines, left) and at day 90 (*n* = 4 individual hCO from two hiPS cell lines, right). Data presented as mean ± s.d. One-way ANOVA with Tukey’s post hoc test: day 30, *F*_5,12_ = 5.131, *P* = 0.0095; day 90, *F*_5,18_ = 36.81, *P* < 0.0001, ****P < 0.0001. **f**, Flow cytometry of hCO (day 152) following 2 days of incubation with 1 μM Cy5-ASO.14. hCO were dissociated and stained with neuronal cell surface protein CD90; non-treated hCO were used as control (Supplementary Fig. 1). Source Data

**Extended Data Fig. 3:**
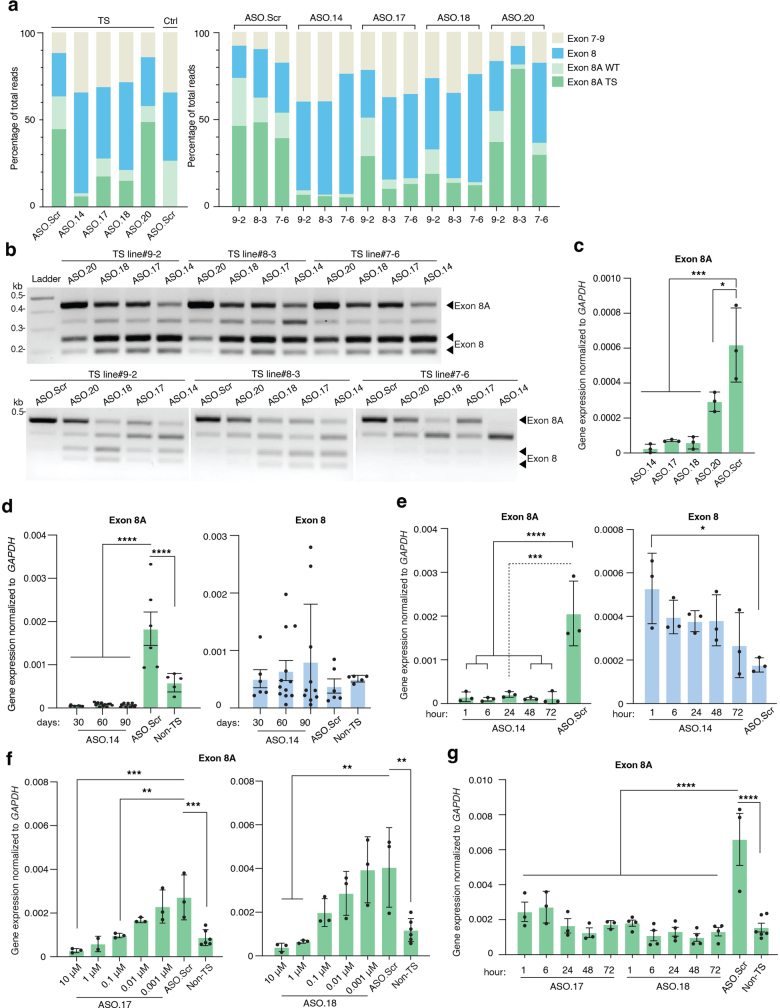
ASOs modulate *CACNA1C* splicing in human neurons. **a**. Amplicon sequencing of the ASO-treated hCO (differentiation day 60, 10 μM ASO). Left: percent of PCR products. Right: data from the left panel separated by TS lines. **b**. RFLP of ASOs-treated hCO for 15 days (upper) and 30 days (lower). **c**. RT-qPCR of exon 8A (differentiation day 30, 1 μM ASO, n = 3). One-way ANOVA with Tukey’s post hoc test: F_4,10_ = 18.59, P = 0.0001, ***P < 0.0001. **d**. qPCR of exon 8A and 8 (1 μM ASO). (n = 6 for day 30 ASO.14; n = 12 for day 60 ASO.14; n = 10 for day 90 ASO.14; n = 6 for TS ASO.Scr; n = 5 for non-TS ASO.Scr.) One-way ANOVA with Tukey’s post hoc test: for exon 8A, F_4, 34_ = 27.91, P < 0.0001; **** P < 0.0001; for exon 8, F_4, 34_ = 0.4948, P = 0.7396. **e**. RT-qPCR of exon 8A (left) and exon 8 (right) (1 μM ASO, n = 3). One-way ANOVA with Tukey’s post hoc test: for exon 8A, F_5,12_ = 18.96, P < 0.0001, ****P < 0.0001, ***P = 0.0001; for exon 8, F_5, 12_ = 3.654, P = 0.0306, *P < 0.05. **f**. RT-qPCR of exon 8A (differentiation day 75, 1 μM ASO) (n = 3 for TS and TS ASO except for ASO.17 1 μM where n = 2 and this was not included in the comparison, n = 6 for non-TS control). One-way ANOVA with Dunnett’s correction: for ASO.17, F_5,15_ = 10.33, P = 0.0002; **P < 0.005, ***P ≤ 0.0005; for ASO.18, F_6,17_ = 7.900, P = 0.0004; **P < 0.005. **g**. qPCR of exon 8A (differentiation day 74, 1 μM) (n = 3 for ASO.17, n = 4 for ASO.18, n = 6 for non-TS control). One-way ANOVA with Tukey’s post hoc test to compare ASO.17 and ASO.18 to ASO.Scr: F_11,32_ = 9.775, P < 0.0001; ****P < 0.0001. Each dot represents an individual hCO from a different hiPS cell line for **c**-**g**. Data are presented as mean ± s.d. for **c**-**g**. Source Data

**Extended Data Fig. 4:**
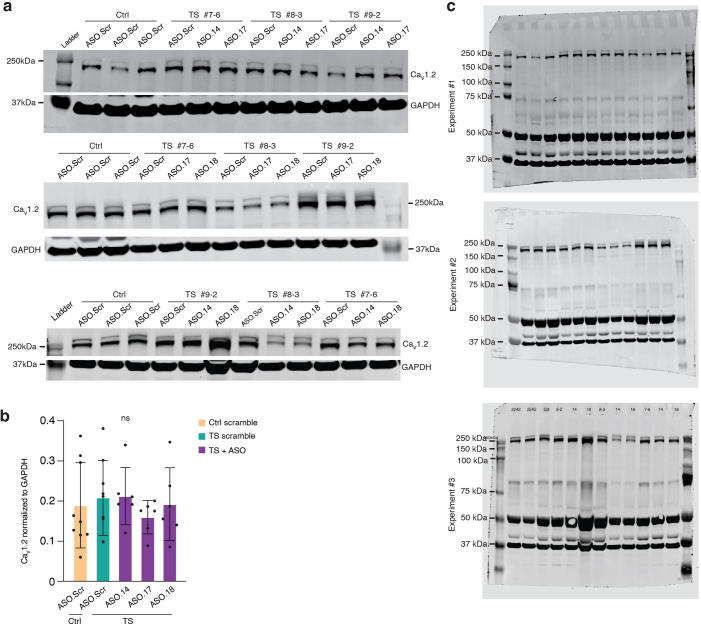
Western blot of human Ca_V_1.2 in ASO-treated hCO. **a**. Western blot of hCO treated with ASO.14, ASO.17, ASO.18 or ASO.Scr (differentiation day 70-80). **b**. Ca_V_1.2 protein blot normalized to GAPDH. Each dot represents an individual sample containing 2–3 hCO from an independent experiment (Ctrl: n = 8; TS Scr: n = 8; TS ASO.14: n = 6; TS ASO.17: n = 6; TS ASO.18: n = 6). Data are presented as mean ± s.d. One-way ANOVA with Tukey’s post hoc test: F_4,31_ = 0.3548, P = 0.8387. **c**. Raw images of the western blots corresponding to **a**–**b**. Source Data

To screen for ASOs that could modify exon 8 splicing, we designed an ‘ASO walking’ strategy (ref. ^21^) with 5-nucleotide (nt) spacing covering exon 8A. We used ASOs with a universal 2′-*O*-methoxyethylribose (MOE) modification to avoid potential degradation of *CACNA1C* mRNA^18,19^ (Fig. 2a). We differentiated TS hiPS cells into hCO, dissociated them into two-dimensional neural cultures and added 10 μM ASO targeting either exon 8A or a scrambled control ASO (ASO.Scr or A.Scr). Three days later, quantitative PCR with reverse transcription (RT–qPCR) of exons 8 and 8A showed that several ASOs had induced robust downregulation of exon 8A without changing exon 8 expression (Fig. 2b). To validate these results in three-dimensional hCO we selected the top four ASOs (ASO.14, ASO.17, ASO.18 and ASO.20). Exposure to ASOs for 3 days in three-dimensional organoid cultures also yielded selective exon 8A downregulation, as shown by both RT–qPCR analysis (Fig. 2c; *P < 0.05, **P < 0.001) and RFLP (Fig. 2d). Sequencing these amplicons further confirmed that ASO.14, ASO.17 and ASO.18 targeted and downregulated exon 8A in hCO derived from three patients with TS (Extended Data Fig. 3a). These effects were long-lasting; a single ASO administration effectively suppressed exon 8A up to 90 days post-exposure (Extended Data Fig. 3b,d; *P* < 0.05). Moreover, the switch from 8A to 8 was not associated with changes in the total amount of Ca_V_1.2 protein, as indicated by immunoblots of hCO (Extended Data Fig. 4a–c).

**Extended Data Fig. 5:**
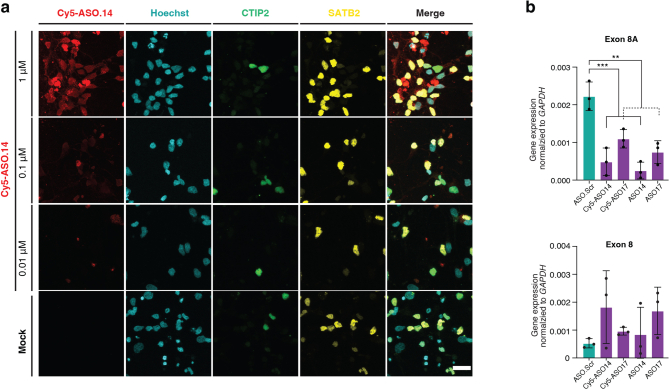
Cy5-labeled ASO in hCO. **a**. CTIP2 and SATB2 immunostaining in hCO (differentiation day 210) dissociated and cultured in 2D. Cy5-ASO.14 was added to the 2D culture for 3 days before immunostaining. **b**. RT-qPCR analysis of exon 8A and exon 8 from TS hCO that were incubated with ASOs for 3 days. n = 3 hCO derived from 3 TS hiPS cells. Data are presented as mean ± s.d. One-way ANOVA with Tukey’s post hoc test: for exon 8A, F_4,10_ = 18.66, P = 0.0001, ***P < 0.001, **P < 0.01; for exon 8, F_4, 10_ = 1.370, P = 0.3117. Source Data

To study the pharmacodynamics of these ASOs, we treated hCO with different concentrations of ASO.14 ranging from 0.001 to 10 μM at differentiation days 30 and 90. We observed a dose-dependent decrease in exon 8A expression (Fig. 2e; *P < 0.05 for day 30, ****P < 0.0001 for day 90). We then treated hCO with ASO.14 and performed RT–qPCR analysis at 1, 6, 24, 48 and 72 h following exposure. Surprisingly, we found that ASO exposure altered the expression of exon 8A as early as 1 h post exposure in vitro (Extended Data Fig. 3e; *P* < 0.001). The pharmacodynamics of ASO.17 and ASO.18 were similar to that of ASO.14 (Extended Data Fig. 3f,g). Finally, to demonstrate the penetration efficacy of ASOs, we labelled ASO.14 with Cy5 and quantified Cy5^+^ cells isolated from hCO by flow cytometry. Most cells, including CD90-expressing neurons, were Cy5^+^ (Fig. 2f). Moreover, 3 days of exposure to Cy5-ASO correlated well with a dose-dependent reduction in Cy5 fluorescence by immunostaining (Extended Data Fig. 5a,b). This indicates that human neurons take up ASO and can, in a dose-dependent manner and within a short period of time, reduce exon 8A expression in this TS model.

**Extended Data Fig. 6:**
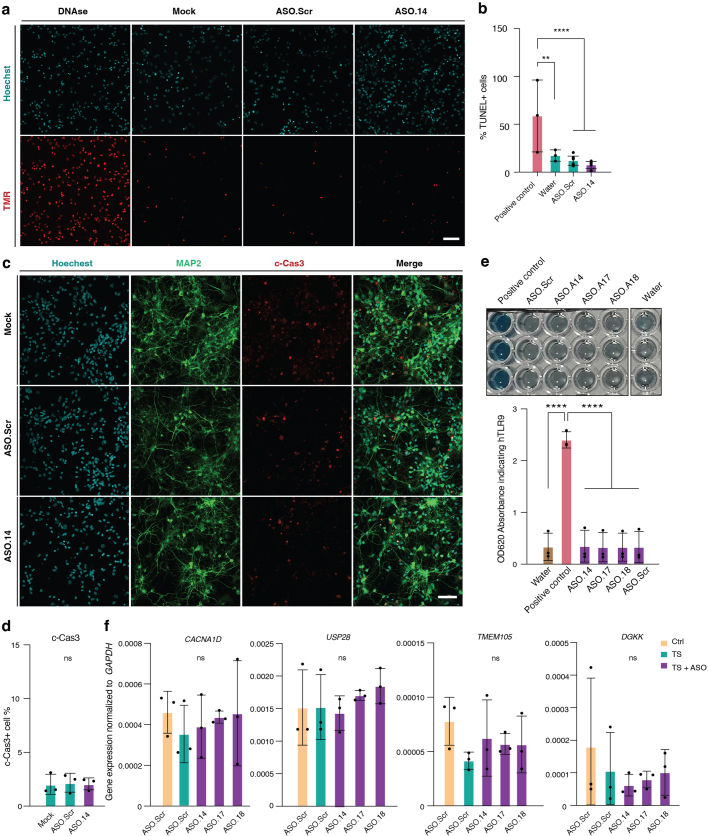
ASO toxicity and off-target effects. **a**. TUNEL staining (red) in dissociated TS neurons exposed to ASOs for 2 days. hCO derived from 3 TS lines (differentiation day 210) were used. Hoechst (blue) stains DNA. DNase treatment was used as a positive control (scale bar 50 μm). **b**. Percentage of TUNEL^+^ cells from **6a**. Each dot represents the averaged percentage of TUNEL^+^ cells on multiple images from one coverslip. Three TS lines were used (n = 3). F_3,20_ = 13.13, P < 0.0001; ****P < 0.0001, **P = 0.0033. **c**. Immunocytochemistry of cleaved caspase 3 (c-Cas3) in dissociated TS neurons (differentiation day 210) exposed to ASOs for 2 days (n = 3 TS lines; scale bar 50 μm). **d**. Percentage of c-Cas3^+^ cells. Each dot represents the average percentage of c-Cas3 from multiple images taken from one coverslip (n = 3 TS lines). F_2,6_ = 0.03288, P = 0.9678. **e**. Human TLR9 reporter HEK 293 cells assay to measure NF-κB-dependent TLR9 signaling. The hTLR9 ligand ODN was used as a positive control. Three independent experiments were performed. Two-way ANOVA: F_4,48_ = 379.1, P < 0.0001; ****P < 0.0001. **f**. qPCR of *CACNA1D*, *USP28*, *TEME105 and DGKK* to evaluate off target effects. hCO were exposed to 1 μM ASOs for 3 days (n = 3 TS lines and 3 control lines). For *CACNA1D*, F_4,10_ = 0.2715, P = 0.8897; for *USP28*, F_4,10_ = 0.5381, P = 0.7114; for *DGKK*, F_4,10_ = 0.4405, P = 0.7769; for *TEME105*, F_4,10_ = 1.012, P = 0.4461. Data are presented as mean ± s.d. in **b**, **d**-**f**. One-way ANOVA with Tukey’s post hoc test for **b**, **d** and **f**. Source Data

Moreover, to identify adverse effects of ASOs in human neural cells we measured their toxicity, immunogenicity and off-target effects^27^ (Extended Data Fig. 6). In both TUNEL assay and cleaved caspase 3 (c-Cas3) staining used for estimation of apoptosis we found no differences among ASO.Scr-, ASO.14- and mock-exposed neurons (Extended Data Fig. 6a–d). hTLR9 reporter cells are used to evaluate the immunogenicity of exogenous DNA^28^ and we did not detect hTLR9 signalling activation following ASO delivery (Extended Data Fig. 6e). Because the universal MOE modification of our ASOs does not recruit the RNase H1 pathway, this is unlikely to cause off-target gene knockdown. Nonetheless, we performed qPCR analysis for *CACNA1D* encoding Ca_v_1.3, another L-type calcium channel, and top off-target gene candidates based on sequence homology. We found no significant differences among ASO-treated groups, the ASO.Scr group and the control group (Extended Data Fig. 6f). These preliminary results evaluating ASO adverse effects in vitro are consistent with previous studies on ASO toxicity and off target^27,28^.

## ASO exposure rescues delayed channel inactivation and interneuron migration defects in TS hCO and hFA

**Extended Data Fig. 7:**
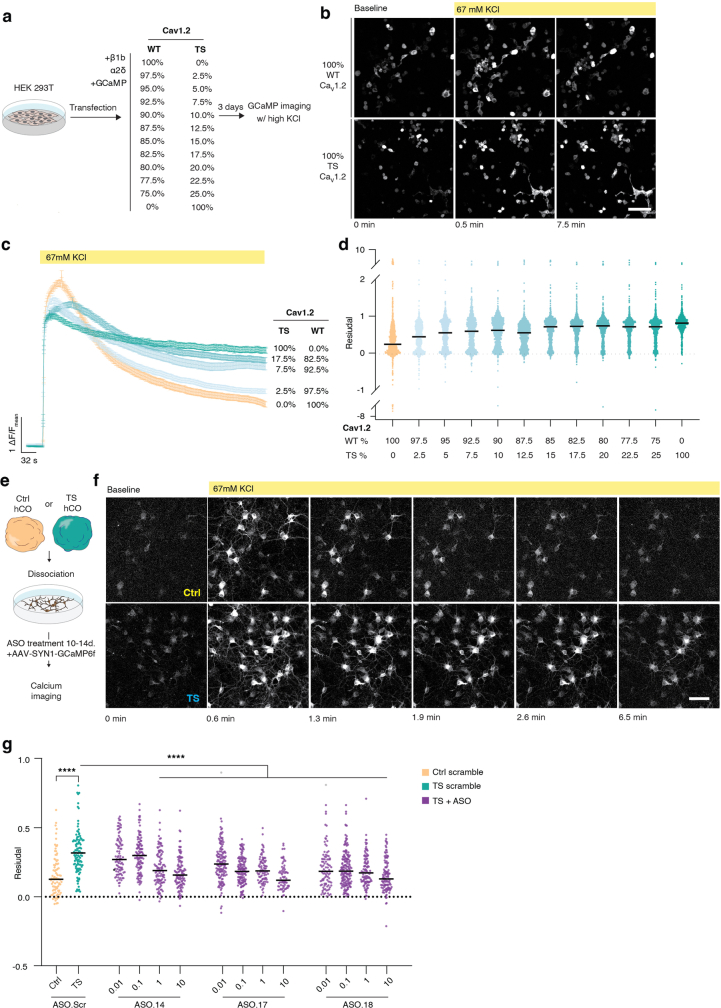
Impact of different *CACNA1C* isoform ratios on calcium signaling and threshold estimates for ASO rescue. **a**. Experimental design. Each condition contains a mix of two plasmids encoding either the WT or the TS Ca_V_1.2 that are co-transfected with plasmids encoding the β1b, α2δ subunit of the channel plus GCaMP6-x. **b**. Representative images of GCaMP imaging before and after 67 mM KCl application. Transfected cells contain only WT Ca_V_1.2 (upper panel) or only TS Ca_V_1.2 (lower panel). Scale bar 100 μm. **c**. Representative traces of chemically induced intracellular GCaMP signal. (0% TS + 100% WT, n = 257 cells; 2.5% TS + 97.5% WT, n = 388 cells; 7.5% TS + 92.5% WT, n = 335 cells; 17.5% TS + 82.5% WT, n = 325 cells; 100% TS + 0% WT, n = 190 cells). Data are presented as mean ± s.e.m. **d**. Comparison of residual Ca^2+^ measured after 67 mM KCl application. Each dot represents one cell (n = 11,228 cells). One-way ANOVA with Dunnett’s correction was used was used to compare TS Ca_V_1.2 to WT: F_11, 11266_ = 68.73, P < 0.0001, ****P < 0.0001. **e**. Experimental procedure for evaluating rescue by ASOs at various concentrations. **f**. Representative images of GCaMP6f imaging acquired before and after 67 mM KCl application to control (upper panel) and TS neurons (lower panel) neurons. Control and TS neurons are derived from isogenic hiPS cells (242 to 254 days of differentiation). Scale bar 50 μm. **g**. Comparison of residual Ca^2+^ measured by GCaMP6f after 67 mM KCl exposure. Each dot represents one cell (n = 1,527 cells). One-way ANOVA with Dunnett’s correction was used to compare ASO.14, ASO.17 and ASO.18 to TS ASO.Scr: F_13, 1513_ = 20.45, P < 0.0001; ****P < 0.0001. Source Data

We previously demonstrated that TS cortical neurons show delayed inactivation of barium currents, increased intracellular calcium following depolarization and impaired interneuron migration^3^. To gain further insights into the threshold of TS Ca_V_1.2 expression necessary to detect a cellular phenotype, we measured depolarization-induced residual Ca^2+^ signal in HEK293T cells expressing 12 variable-ratio combinations of WT and TS Ca_V_1.2 (Extended Data Fig. 7a–d). We detected a significant difference in residual Ca^2+^ between WT and TS Ca_V_1.2 (Extended Data Fig. 7c,d) and found that even a small proportion of TS Ca_V_1.2 is sufficient to perturb the kinetics of channel inactivation (Extended Data Fig. 7c,d). This highlights the effect of TS Ca_V_1.2 on calcium influx and further indicates ASO therapeutic potential, even at postnatal stages when exon 8A expression is lower than prenatally.

**Fig. 3:**
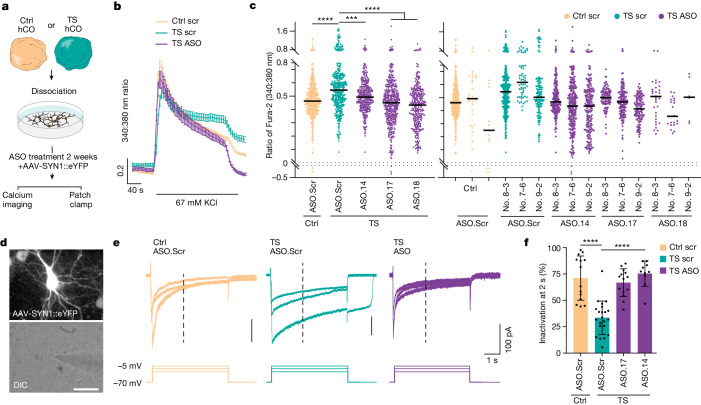
ASO exposure rescues delayed channel inactivation in TS cortical neurons. **a**, Strategy used to evaluate the effect of ASO on human neurons. **b**, Representative traces of depolarization-induced calcium responses measured by Fura-2 imaging (control scramble (Scr), *n* = 55 cells; TS scramble, *n* = 31 cells; TS + ASO.14, *n* = 24 cells). Data presented as mean ± s.e.m. **c**, Residual calcium in ASO-treated neurons (days 100–120 of differentiation). Left, data pooled across hiPS cell lines; right, data separated by cell line. Each dot represents one cell (*n* = 2,017 cells); Kruskal–Wallis test, *P* < 0.0001. Control versus TS, ****P < 0.0001; TS versus ASO.14, ***P < 0.001; TS versus ASO.17, ****P < 0.0001; TS versus ASO.18, ****P < 0.0001. Data presented as mean ± s.e.m. DIC, differential interference contrast. **d**, Representative example of patch-clamp recordings from AAV-SYN1::eYFP-infected hCO neurons. Scale bar, 20 µm **e**, Representative examples of barium currents following 5 s depolarization steps (–70 to –25, –15 and –5 mV, respectively). **f**, Summary graph of barium current inactivation (percentage of inactivated current compared with amplitude of peak current at 2 s) for maximal current. Ctrl Scr, *n* = 14 cells from two lines; TS Scr, *n* = 22 cells from two lines; TS ASO.17, *n* = 14 cells from two lines; TS ASO.14, *n* = 10 cells from one line. Data presented as mean ± s.d. One-way ANOVA with Tukey’s post hoc test, *F*_3,56_ = 25.34, *P* < 0.0001, ****P < 0.0001. Source Data

**Extended Data Fig. 8:**
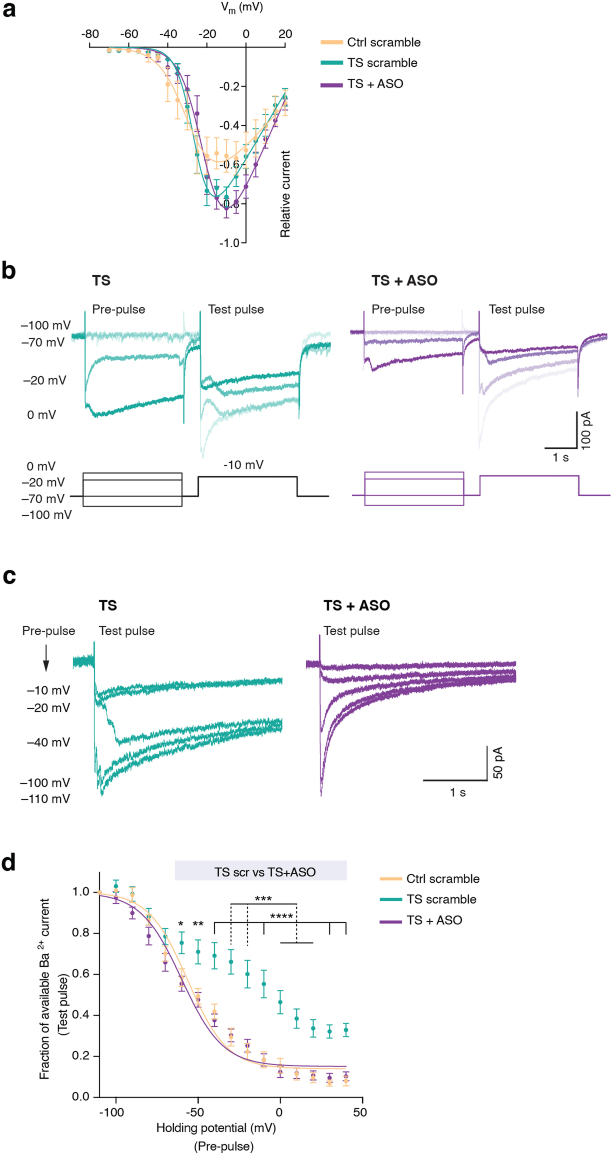
Patch-clamp of ASO-treated TS and control neurons. **a**. I-V curves of barium current amplitudes recorded from hCO neurons. I-V curves were fitted with Boltzmann exponential functions (Ctrl scramble, n = 11 cells; TS scramble, n = 15 cells, TS + ASO, n = 13 cells). Data are mean ± s.e.m. A mixed-effects model with the Geisser-Greenhouse correction was used for comparison among groups: F_2,36_ = 1.021, P = 0.3706. **b**. Upper: representative traces of barium currents in 3-second-long pre-pulse depolarization to –100 mV, –70 mV, –20 mV and 0 mV followed by 3-second-long test pulse depolarization to 0 mV. Lower: representative traces of barium currents in the test pulse depolarizations after pre-pulse depolarization. **c**. Voltage dependence of barium current inactivation with a test pulse to 0 mV after a series of pre-pulses from –110 mV to +40 mV with an increment of 10 mV. **d**. Voltage-dependent inactivation curves of Ctrl and TS + ASO were fitted with exponential functions (Ctrl Scr: n = 14 cells from 2 hiPS cell lines; TS Scr: n = 15 cells from 2 hiPS cell lines; TS ASO.17: n = 6 cells and TS ASO.14: n = 6 cells from 2 hiPS cell lines). Data are presented as mean ± s.e.m. A mixed-effects model with the Geisser-Greenhouse correction was used for comparison among groups: F_2,38_ = 16.42, P < 0.0001. Turkey’s multiple comparisons test was used for comparison between TS and TS + ASO groups. *P = 0.0132, ***P < 0.001, ****P < 0.0001. Source Data

Next we tested whether alteration of exon 8A/8 splicing via ASOs could restore Ca_V_1.2 channel function. We exposed TS hCO neurons to ASO.14, ASO.17, ASO.18 or ASO.Scr and compared these with control hCO neurons exposed to ASO.Scr in a Fura-2 AM calcium imaging assay (Fig. 3a). As expected, TS neurons showed slower decay kinetics following depolarization compared with control neurons (Fig. 3b,c; ****P < 0.0001). All three selected ASOs restored residual calcium to control levels, suggesting that ASOs can functionally rescue the TS Ca_V_1.2 channel (Fig. 3b,c; *P* < 0.001). We then applied ASO.14 and ASO.17 (the latter has effects similar to ASO.18) to both TS and control hCO and performed whole-cell patch-clamping of neurons labelled by SYN1:YFP (Fig. 3a,d). TS neurons showed delayed inactivation of barium currents as measured by percentage channel inactivation following 2 s of current-clamping (Fig. 3e, Extended Data Fig. 8a–d). Similar to the delayed inactivation we observed with calcium imaging, this defect was rescued by both ASO.14 and ASO.17 (Fig. 3f; ****P < 0.0001). To further explore the functional rescue of ASOs we set up a scalable GCaMP6f imaging readout on dissociated hCO neurons (Extended Data Fig. 7e,f). Following the application of single doses of ASOs at various concentrations for 10 days, we measured GCaMP6f signals before and after acute KCl depolarization. We found that, for all three ASOs tested, both 1 and 10 μM effectively rescued the TS phenotype whereas neither 0.1 nor 0.01 μM for ASO.14 did (Extended Data Fig. 7e–g). This suggests that there might be a discrepancy between the levels of RNA expression and protein function for TS rescue. Consistent with this, one recent ASO study found that a highly efficient knockdown of UBE3A-ATS was required to elevate the Ube3a protein level, yet UBE3A protein continued to increase with higher ASO concentrations even when mRNA restoration plateaued^29^.

**Fig. 4:**
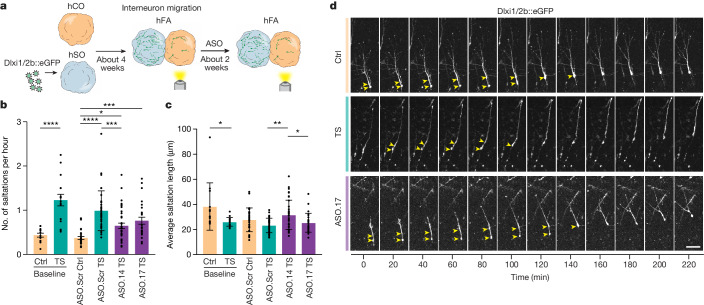
ASO exposure rescues delayed migration defects in TS hFA. **a**, Strategy used to test the effect of ASO on interneuron migration using hFA. Preceding fusion of hSO and hCO, hSO were infected with cortical interneuron reporter Lenti-Dlxi1/2b::eGFP around day 40. Imaging was performed at 4 weeks following assembly and again at 2 weeks post ASO incubation. **b**, Saltation frequency of Dlxi1/2b::eGFP^+^ migrating cortical interneurons in hFA. Pre ASO exposure, *n* = 13 Ctrl cells and *n* = 16 TS cells; post ASO exposure, *n* = 30 Ctrl ASO.Scr, *n* = 37 TS ASO.Scr, *n* = 38 TS ASO.14 and *n* = 26 TS ASO.17 cells. Data presented as mean ± s.d. One-way ANOVA with Tukey’s post hoc test for post-ASO exposure groups, *F*_3,125_ = 14.03, *P* < 0.0001, ****P < 0.0001, ***P = 0.0009, *P = 0.0177. Two-tailed unpaired *t*-test with Welch’s correction was used to compare baseline control and TS, ****P < 0.0001. **c**, Saltation length of Dlxi1/2b::eGFP^+^ migrating cortical interneurons in hFA. Data presented as mean ± s.d. One-way ANOVA with Tukey’s post hoc test for post-ASO exposure groups, *F*_3,125_ = 5.648, *P* = 0.0012, **P = 0.0007, *P = 0.0376. Two-tailed unpaired *t*-test with Welch’s correction was used to compare baseline control and TS, *P = 0.0386. **d**, Representative images of saltatory movement (yellow arrowheads) of Dlxi1/2b::eGFP^+^ migrating cortical interneurons; scale bar, 50 μm. Source Data

We previously discovered that TS interneurons migrate abnormally in hFA^3,4^. To investigate whether ASOs can correct this cellular migratory defect in three-dimensional cultures we derived TS and control hCO and hSO, labelled interneurons in hSO with a lineage-specific reporter (LV.Dlxi1/2b::eGFP) and generated hFA, as previously demonstrated^3^ (Fig. 4a–d). Three to four weeks post assembly we imaged and quantified saltation frequency and the average saltation length of TS and control interneurons at baseline; we then exposed hFA to ASO.14, ASO.17 or ASO.Scr and performed a further imaging experiment 2 weeks later. At baseline before ASO exposure, as previously described, we found increased saltation frequency (Fig. 4b; *P < 0.05, ***P < 0.005, ****P < 0.0001) and shortened saltation length in TS interneurons compared with control interneurons (Fig. 4c; *P* < 0.05). Exposure to ASO.14 and ASO.17 reduced the saltation frequency of TS interneurons (Fig. 4b; *P* < 0.05) and increased saltation length (Fig. 4c; *P* < 0.05). In summary, we found that exposure to exon 8A–8-switching ASOs effectively rescued channel function, calcium signalling dynamics and cellular phenotypes in in vitro cultures derived from patients with TS.

## ASO delivery in vivo rescues TS-related phenotypes in transplanted human TS cells

**Fig. 5:**
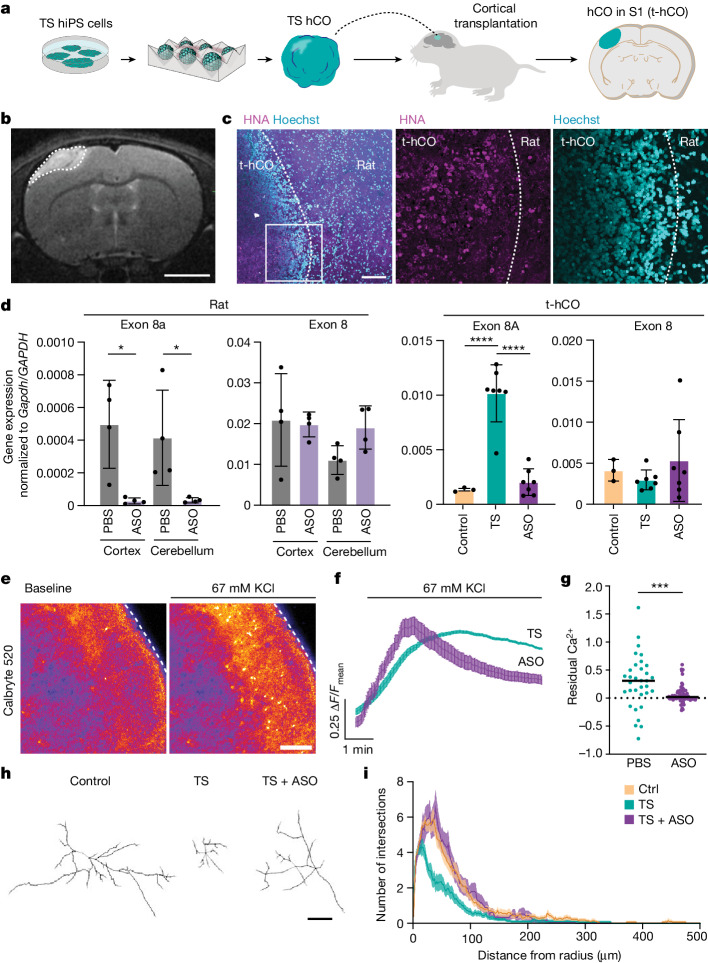
ASO delivery in vivo rescues TS-related phenotypes in transplanted human TS cells. **a**, Schematic illustrating transplantation of hCO (t-hCO) into rat somatosensory cortex. **b**, Representative MRI showing t-hCO (scale bar, 4 mm). **c**, Immunostaining in t-hCO for the human-specific marker HNA (scale bar, 2 mm). **d**, RT–qPCR analysis of t-hCO (days 162–258) and rat neural tissue following ASO injection. Data presented as mean ± s.d. Left, exons 8A and 8 of rat *Cacna1c* in cerebral cortex and cerebellum (*n* = 4 animals per group); two-sided unpaired student’s *t*-tests were used to compare ASO versus PBS in cortex (*P* = 0.0129) and ASO versus PBS in cerebellum (*P* = 0.0382). Right, exons 8A and 8 of human *CACNA1C* (Ctrl, *n* = 4; TS, *n* = 7; TS + ASO, *n* = 7; t-hCO. each point represents either qPCR or average qPCR value from t-hCO from the same animal. The same t-hCO samples were also used for the RFLP assay shown in Extended Data Fig. 10a). One-way ANOVA with Tukey’s post hoc test: for exon 8A, *F*_2,14_ = 40.40, *P* < 0.0001, ****P < 0.0001; for exon 8, *F*_2,14_ = 0.8211, *P* = 0.4601. **e**, Calbryte 520-based calcium imaging of t-hCO. Slices of t-hCO were incubated with the dye for 1 h and then imaged on a confocal microscope before and after stimulation by 67 mM KCl; scale bar, 100 μm. **f**, Representative traces of responses to Calbryte 520 imaging. **g**, Residual calcium in Calbryte 250-based imaging of t-hCO (PBS treated, *n* = 33; ASO.14, *n* = 77; Mann–Whitney test, two-tailed, ***P = 0.0002). **h**, Representative images of cell morphology tracing with Golgi staining; scale bar, 50 μm. **i**, Sholl analysis of Ctrl, TS and TS + ASO neurons in t-hCO (*n* = 24 Ctrl t-hCO neurons, *n* = 24 TS t-CO neurons, *n* = 11 TS + ASO t-hCO neurons). Data presented as mean ± s.e.m. Source Data

Encouraged by these findings and motivated to assess the translational potential of these ASOs in TS, we next validated their effect in an in vivo setting. We have recently developed a strategy for transplantation into the developing cerebral cortex of early postnatal rats that allows hCO to develop mature cell types and integrate both anatomically and functionally into the rodent brain^7^. We now applied this in vivo platform to test the delivery of ASOs in vivo and their ability to rescue genetic and functional defects in cells from patients with TS1 (Fig. 5a,b).

**Extended Data Fig. 9:**
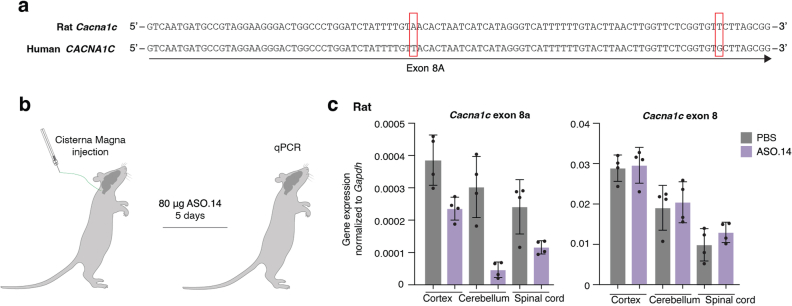
ASO delivery and effectiveness in rats that contain no t-hCO. **a**. Sequence alignment between exon 8a of rat *Cacna1c* and exon 8A of human *CACNA1C*. Red boxes indicate mismatched nucleotides. **b**. Schematic showing injection of 80 μg ASO.14 into the rat cisterna magna. Rat brain, cerebellum and spinal cord were collected 5 days post-injection. **c**. RT-qPCR analysis of rat *Cacna1c* exon 8a and exon 8 after ASO injection (n = 2 animals; for each sample, 2 pieces of adjacent tissue (technical replicates) were collected for each brain region: cortex, cerebellum and spinal cord). Data are presented as mean ± s.d. Source Data

We first tested whether our ASOs would act on rat brain tissue, in particular because rat *Cacna1c* is highly homologous to human *CACNA1C* (Extended Data Fig. 9a). To this end, in the rat cisterna magna we injected 80 μg of ASO.14, an ASO that robustly suppresses exon 8A expression in vitro. Five days later we discovered that ASO.14 had reduced *Cacna1c* exon 8A expression in the cortex, cerebellum and spinal cord (Extended Data Fig. 9b,c).

**Extended Data Fig. 10:**
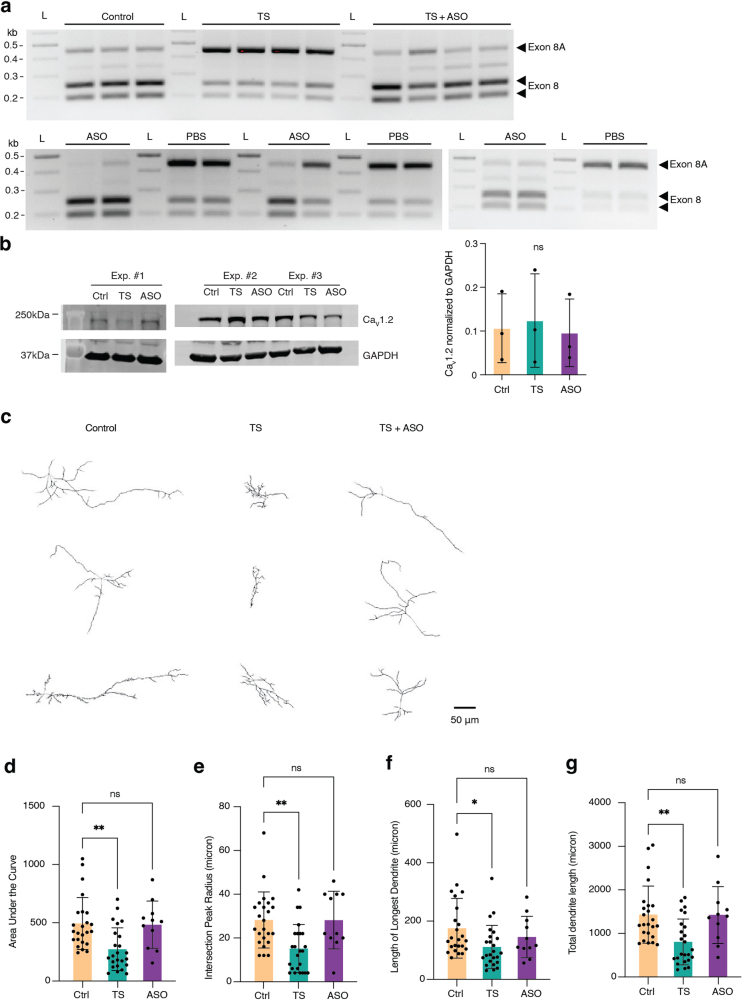
Effect of in vivo ASO administration on dendrite morphology in TS. **a**. RFLP analysis of t-hCO. Upper panel: n = 3 control, n = 4 TS, n = 4 for TS + ASO t-hCO. Lower panel: n = 3 t-hCO per group. The two gel columns for each conditions represent two pieces cut from same t-hCO. The same t-hCO were used for qPCR shown in Fig. 5d. **b**. Western blot of t-hCO treated with ASO.14 or PBS. Data are presented as mean ± s.d. n = 3 individual t-hCO extracted from the rat cortex. One-way ANOVA with Tukey’s post hoc test: F_2,6_ = 0.07231, P = 0.9310. Gel source data is shown in Supplementary Fig. 2. **c**. Representative images of cell morphology tracing using Golgi staining. **d**. Analysis of area under the curve (n = 24 control t-hCO neurons, n = 24 TS t-CO neurons, n = 11 TS + ASO t-hCO neurons). F_2,56_ = 8.134, P = 0.0008; **P = 0.0012. **e**. Quantification of intersection peak radius (n = 24 control t-hCO neurons, n = 24 TS t-CO neurons, n = 11 TS + ASO t-hCO neurons). F_2,56_ = 8.225, P = 0.0007; **P = 0.0013. **f**. Comparison of the longest dendrite length among groups (n = 24 control t-hCO neurons, n = 24 TS t-CO neurons, n = 11 TS ASO t-hCO neurons). F_2,56_ = 3.266, P = 0.0455; *P = 0.0352. **g**. Comparison of total dendrite length among groups (n = 24 control t-hCO neurons, n = 24 TS t-CO neurons, n = 11 TS + ASO t-hCO neurons). F_2,56_ = 7.826, P = 0.0010; **P = 0.0017. For **d**-**g**, Data are presented as mean ± s.d. and one-way ANOVA with Dunnett’s correction was used. Source Data

We next transplanted hCO from three individuals with TS and monitored t-CO and graft position by magnetic resonance imaging (MRI) and immunostaining (Fig. 5b,c). We then injected 300 μg of ASO.14 into rat cisterna magna. Seven to 14 days later we extracted the hCO graft and found that *CACNA1C* exon 8A in TS t-hCO had reduced the level of expression (*P* < 0.0001; Fig. 5d and Extended Data Fig. 10a). This was accompanied by a reduction in the expression of rat *Cacna1c* exon 8a in both cortex and cerebellum (Fig. 5d; *P* < 0.05). Similar to the in vitro ASO experiments, overall Ca_V_1.2 levels were not affected (Extended Data Fig. 10b). This experiment indicates that ASOs can be delivered intrathecally and can effectively modulate splicing in human transplanted cells. Lastly, we attempted to verify the effects of ASO administration on cellular dysfunction resulting from the TS1 mutation. To do so we extracted t-hCO, sliced the tissue and performed ex vivo calcium imaging using the calcium indicator Calbryte 520 AM (Fig. 5e). We found that ASO.14 normalized the increase in post-depolarization residual calcium found in cortical TS neurons (Fig. 5f,g). Finally, TS is associated with activity-dependent dendrite morphology defects^5^ and this can be detected in patient-derived cortical neurons following transplantation in vivo^7^. To test whether ASOs could rescue this morphological phenotype, we traced neurons using Golgi staining in t-hCO at 14 days post ASO injection. We found that ASO.14 corrected the dendritic morphology of TS neurons in vivo (Fig. 5h,i and Extended Data Fig. 10c–g).

These experiments indicate that ASOs can modulate splicing of human *CACNA1C* both in vitro and in vivo and thereby rescue both molecular and cellular phenotypes of TS1.

## Discussion

Developing therapies for neuropsychiatric disorders remains a substantial challenge due to the inaccessibility of human brain tissue. This holds true especially for disorders that emerge during fetal development, such as TS. Despite an understanding of the genetic cause and of some of the molecular mechanisms of TS, we still do not have a promising therapeutic avenue. L-type calcium channel blockers do not restore many of the cellular phenotypes in TS, and roscovitine has extensive off-target effects^2,14^. Some, but not all, of the defects identified with human cellular models have been recapitulated in a mouse^30^ expressing the channel with the TS type 2 variant (the p.G406R variant is in exon 8 that also carries a stop codon in exon 8A), suggesting that species-specific differences in gene regulation can change the cellular phenotypes associated with a disease.

Here we developed a potential therapeutic strategy for a severe neurodevelopmental disorder caused by a single nucleotide variant in an alternatively spliced exon. To do this we first investigated splicing profiles in human neurons and found that the persistent elevation of exon 8A in TS is biased towards the TS gain-of-function variant, which probably amplifies defects downstream of this dysfunctional calcium channel. We subsequently screened and identified ASOs that can effectively modulate splicing in TS to reduce exon 8A without changing the overall level of Ca_V_1.2 protein. We demonstrate, in human neurons derived from three patients with TS1 in human organoid and assembloid models that these ASOs can, in a dose- and time-dependent manner, modulate exon 8A and rescue ion flux kinetics, calcium dynamics and associated cellular movement defects. Lastly, we show that ASOs can be delivered in vivo using a organoid transplantation platform that we previously developed and, importantly, that they can rescue splicing and intracellular calcium flux defects in human neurons integrated into the rat cerebral cortex.

There are a number of limitations to our study. First, our current ASOs do not distinguish WT exon 8A and TS exon 8A. Further refinement, including testing ASOs of varying length, chemical modifications and targeting upstream and downstream of the TS variant, may be needed to increase specificity. Longer and earlier exposure to ASOs may also be needed to fully restore migration defects. The p.G406R mutation in TS1 modelled here is in exon 8A of *CACNA1C*. It would be clinically relevant to investigate whether TS2, caused by the same amino acid mutation but in exon 8, also shows abnormal splicing of exon 8/8A and whether ASOs can correct splicing defects and rescue channel function. Because patients with TS have cardiac arrythmias, it would be useful to test the ability of these ASOs to rescue defects in cardiac organoids. Finally, our assessment of ASO toxicity was performed in vitro. Recent studies have shown that ASOs containing a gapmer design can show dose-dependent acute neurotoxicity in the central nervous system^31,32^; therefore, in vivo short- and long-term pharmacology will be necessary to evaluate the relative toxicity of the full MOE-modified, splicing-modulating candidates that we identified. Notably, for some ASOs we observed a strong dose–response with full splice modulation at 10 nM. Futures studies should explore the contribution to this effect by ASO sequence and chemical modification, endogenous pre-mRNA levels, the cell type context as well as the potential off-mechanism effects. Moreover, evaluation of efficacy in vivo will benefit from using nontargeting control of ASO rather than simply PBS. Transplantation of hCO allows unpreceded maturation and circuit integration of human neurons into animals. Of note, this therapeutic approach is unlikely to influence cell specification defects that may take place prenatally, but rather will correct channel dysfunction and associated defects postnatally. Future directions include understanding how the TS variant affects circuit development in vivo, how splicing of exons 8/8A is regulated across brain regions in postnatal primate brain and the functional consequences of this switch. This may give insights into whether there is an optimal developmental window for ASO treatment to rescue these cellular phenotypes.

Our proof-of-concept study, which includes a combination of in vitro and in vivo studies with human patient-derived, three-dimensional, multicellular models, illustrates how this platform could be used to study other neuropsychiatric diseases, and to evaluate therapeutic efficiency and safety, including but not limited to ASOs, viral vectors and small molecules. This will be particularly relevant when animal models are not available or do not fully recapitulate human pathophysiology.

## Methods

### Culture of hiPS and HEK293T cells

The hiPS cells in this study were previously described and validated^2,3^. A total of six hiPS cell lines were derived from fibroblasts collected from three healthy individuals and three with TS. Approval for this study was obtained from the Stanford IRB panel, and informed consent was obtained from all participants. The isogenic TS1 (G406R) line was derived in the KOLF2.1 hiPS cell line via nucleofection using the guide RNA-targeting GGTGTGCTTAGCGG and the homologous repair template ssODN, aggaatagcagaaagaataaataaaaataaatggaaaaatcaagacctttttccttggtcctgcttacCTGCTAAGCACACCGAGAACCAAGTTAAGTAC^33^. The CW30293 hiPS cell line was obtained from CIRM. The presence of the heterozygous mutation was confirmed by Sanger sequencing. hiPS cells were cultured in feeder-free essential 8 medium (E8, Thermo Fisher Scientific, catalogue no. A1517001) without antibiotics and kept in the wells of six-well plates (Corning, catalogue no. 3506) coated for 1 h at room temperature with vitronectin recombinant human protein (VTN-N, Thermo Fisher Scientific, no. A14700) diluted 1:100 to a final concentration of 5 ng ml^−1^ in Dulbecco’s PBS (DPBS), with neither calcium nor magnesium (Thermo Fisher Scientific, catalogue no. 14190136). To facilitate passaging, hiPS cells were first washed with DPBS and then incubated with 0.5 mM EDTA (Invitrogen, catalogue no. 15575020) in DPBS at room temperature for 7 min. Following removal of EDTA solution, cells were seeded in fresh wells of six-well plates coated with VTN-N and containing E8 medium. The hiPS cells used in this study were maintained free of *Mycoplasma* at 37 °C in a humidified-air atmosphere with 5% CO_2_. The lenti-X 293T cell line, a subclone of HEK293T cells, was obtained from Takara Bio (catalogue no. 632180) and cultured in DMEM (Gibco, catalogue no. 10313021) supplemented with 10% fetal bovine serum (Corning, catalogue no. 35016CV) and 1× GlutaMAX (Thermo Fisher Scientific, catalogue no. 35050061). This cell line was chosen because it is compatible with robust plasmid overexpression.

### Generation of hCO and hSO from hiPS cells

The generation of hCO, hSO and hFA was performed as previously described^3,34,35^. In brief, hiPS cells were incubated with Accutase (Innovate Cell Technologies, no. AT-104) at 37 °C for 7–8 min and dissociated into single hiPS cells. Single-cell suspensions were collected in a 50 ml Falcon tube and cell pellets obtained via centrifugation at 300*g* for 3 min. Cell numbers were counted following resuspension of cell pellets. Approximately 3 × 10^6^ cells in 2 ml of E8 medium supplemented with ROCK inhibitor Y-27632 (10 μM, Selleckchem, catalogue no. S1049) were added per well of an AggreWell 800 plate (STEMCELL Technologies, catalogue no. 34815). The plates were then centrifuged at 100*g* for 3 min to allow cells to sink to the bottom of the wells (day 0). Twenty-four hours following cell aggregation (day 1), spheroids were dislodged by pipetting (with a P1000 tip cut at the end) and transferred to ultralow-attachment plastic dishes (Corning, no. 3262) in essential 6 medium (E6, Life Technologies, no. A1516401) supplemented with 2.5 μM dorsomorphin (Sigma-Aldrich, catalogue no. P5499) and 10 μM SB-431542 (Tocris, catalogue no. 1614). From days 2 to 6, E6 medium was changed daily and supplemented with dorsomorphin and SB-431542. In addition the Wnt pathway inhibitor XAV-939 (XAV, 1.25 μM, Tocris, catalogue no. 3748) was added, together with dorsomorphin and SB-431542. On the seventh day in suspension, basal medium was switched to neural medium consisting of Neurobasal A (Life Technologies, catalogue no. 10888), B-27 supplement without vitamin A (B-27, Life Technologies, catalogue no. 12587), GlutaMAX (1:100, Life Technologies, catalogue no. 35050) and 10 U ml^−1^ penicillin-streptomycin (Gibco, catalogue no. 15140122). From days 6 to 24 the neural medium was supplemented with 20 ng ml^−^^1^ epidermal growth factor (EGF, R&D Systems, catalogue no. 236-EG) and 20 ng ml^−1^ basic fibroblast growth factor (FGF, R&D Systems, catalogue no. 233-FB) for 19 days (until day 24), with medium changed daily from days 7–18 and every other day until day 24. From days 25–42 the neural medium contained 20 ng ml^−1^ brain-derived neurotrophic factor (Peprotech, catalogue no. 450-02) and 20 ng ml^−1^ NT3 (Peprotech, catalogue no. 450-03), with medium change every other day. From day 43, hCO were cultured with only neural medium without growth factors. The generation of hSO differs from that of hCO in that, from days 7–12, the neural medium was supplemented with XAV (1.25 μM) in addition to EGF and FGF; from days 13–24 the neural medium was supplemented with XAV (1.25 μM) and SAG (100 nM, EMD Millipore, catalogue no. 566660) in addition to EGF and FGF.

### ASOs

ASOs were 20-nt-long synthesized using the phosphorothioate backbone and with a MOE modification. 5-Methylcytosine was used during synthesis rather than cytosine. ASOs tested on hiPS cell-derived forebrain organoids were purified by standard desalting followed by Na^+^ salt exchange. These ASOs were reconstituted in nuclease-free water at a concentration of 1 mM and stored at −20 °C thereafter for in vitro experiments. For in vivo injection, ASO.14 was reconstituted at a concentration of 10 μg μl^−1^ in DPBS for injection of 30 μl of 300 μg ASO into rat cisterna magna. All ASOs used in this study were manufactured by Integrated DNA Technologies. Cy5-labelled ASOs were synthesized by the addition of Cy5 to the 5′ of the ASO (Integrated DNA Technologies) followed by HPLC purification and Na^+^ salt exchange.

### Recombinant DNA and viruses

pDup4-1 was obtained from Addgene (plasmid no. 23022) and was used as the backbone for the minigene splicing reporter. pDup4-1 was digested with ApaI and BglII (New England Biolabs) and the resulting 4,595 bp fragment was purified following loading on a 1% agarose gel using the QIAquick PCR Purification Kit (Qiagen, catalogue no. 28106). Genomic DNA from TS hiPS cells was purified with the DNeasy Blood & Tissue Kit (Qiagen, catalogue no. 69506). Amplicons encompassing exons 8 and 8A of *CACNA1C* were amplified with GoTaq Long PCR Master Mix (Promega, catalogue no. M4021). Primer sequences and cycling conditions are listed in Supplementary Tables 1 and 2. Purified PCR products were digested with ApaI and BglII. Following one further round of purification, DNA was dephosphorylated with FastAP thermosensitive alkaline phosphatase (Thermo Fisher Scientific, catalogue no. EF0654) then ligated to the pDup4-1 backbone using T4 DNA ligase (Thermo Fisher Scientific, catalogue no. EL0011). Following transformation (One Shot Stbl3 Chemically Competent *E. coli*, Thermo Fisher Scientific, catalogue no. C737303), colonies were picked for sequence verification. The human PTBP1 ORF plasmid was obtained from Genscript (clone ID OHu15891D, accession no. NM_002819.5). Plasmids encoding WT and TS Ca_V_1.2 were synthesized by VectorBuilder based on transcript ENST00000399655.6 under a CAG promoter into a lentivirus backbone. An HA tag was placed in between Q683 and T684. The GCaMP plasmid was obtained from Addgene (plasmid no. 111543). Plasmids encoding the β1b and a2δ subunits of the L-type calcium channel were described previously^5^. The maps and sequences of minigene splicing reporters and human Ca_V_1.2 expression plasmids are included in Supplementary Figs. 3–6 (generated by SnapGene 5.1.4.1, SnapGene software from Dotmatics).

### RNA extraction and qPCR

For all samples, RNA was extracted using the RNeasy Plus Mini Kit (Qiagen, catalogue no. 74136). Unless otherwise noted, reverse transcription was performed using the SuperScript III First-Strand Synthesis SuperMix for qRT-PCR (Invitrogen, catalogue no. 11752050) according to the manufacturer’s instructions. qPCR was performed on a QuantStudio 6 Flex Real-Time PCR system (Thermo Fisher Scientific, catalogue no. 4485689) using SYBR Green PCR Master Mix (Thermo Fisher Scientific, catalogue no. 4312704). Primers for qPCR are listed in Supplementary Tables 1 and 2.

### Transcript analysis of *CACNA1C* exons 8 and 8A

Restriction fragment-length polymorphism analysis of *CACNA1C* exons 8 and 8A was performed on PCR fragments amplified from cDNA. DNA was purified using AMPure XP beads (Beckman Coulter, catalogue no. A63881) according to the manufacturer’s instructions. Purified DNA was digested with BamHI (Thermo Fisher Scientific, catalogue no.ER0055) at 37 °C for 3 h and loaded on 2% agarose gel. Gel images were taken on a Gel Doc XR+ imager (Bio-Rad, catalogue no. 1708195). For next-generation sequencing analysis of transcripts, primers with the Illumina adaptor were used to amplify the region encompassing exons 7–9. Following bead purification, DNA was eluted in water and sent for sequencing using the Genewiz Amplicon-EZ module. Next-generation sequencing analysis of the minigene splicing reporter was performed similarly by amplifying minigene transcripts from the cDNA of transfected HEK cells 3 days post transfection. Primers and cycling conditions are listed in Supplementary Tables 1 and 2.

### Transfection of HEK cells

Approximately 30,000–75,000 HEK cells were seeded per well of a 24-well plate (Corning, catalogue no. 353047). The following day, plasmids were mixed with 1 mg ml^−1^ PEI MAX (Polysciences, catalogue no. 24765-1) in 50 μl of a 150 mM NaCl solution. Following about 10 s of vigorous vortexing, plasmid mixtures were incubated for 15 min at room temperature and then added to the wells (Supplementary Tables 3–5).

### Dissociation for monolayer culture

Dissociation of hCO for monolayer culture was performed as previously described, with minor optimizations^4^. Coverslips were coated with approximately 0.001875% polyethylenimine (PEI, Sigma-Aldrich, catalogue no. 03880) for 1 h at 37 °C, washed four times with water and dried. On the day of dissociation, betweeen four and six hCO per hiPS cell line were transferred to wells in six-well plates (Corning, catalogue no. 3506) and incubated for 45–60 min at 37 °C with 3 ml of enzymatic dissociation solution. This solution consisted of 30 U ml^−1^ papain (Worthington Biochemical, catalogue no. LS003127), 1× EBSS (Millipore Sigma, catalogue no. E7150), 0.46% d(+)-glucose, 0.5 mM EDTA, 26 mM NaHCO_3_, 10 μM Y-27632, 125 U ml^−1^ deoxyribonuclease I (Worthington Biochemical, catalogue no. LS002007) and 6.1 mM l-cysteine (Millipore Sigma, catalogue no. C7880). Following papain incubation, samples were collected in a 15 ml Falcon tube and centrifuged at 1,200 rpm for 1 min. Following removal of the supernatant, samples were washed with 1 ml of inhibitor solution with 2% trypsin inhibitor (Worthington Biochemical, catalogue no.LS00308) and resuspended in 1 ml of the same solution for trituration. Following trituration, 1 ml of inhibitor solution with 4% trypsin inhibitor was added slowly beneath the cell suspension to create a gradient layer; the gradient solution was then centrifuged at 1,200 rpm for 5 min. Cell pellets were resuspended in culture medium consisting of Neurobasal A supplemented with B-27 and 10 μM Y-27632. Undissociated tissue was removed by passing the cell suspension through a 40 μm cell strainer (Corning, catalogue no. 352340). Finally, dissociated cells were seeded on the coverslip at a density of 50,000 cells per coverslip in 1 ml of culture medium. The inhibitor solution differs from the enzyme solution in that it contains neither papain nor EDTA. All centrifugation steps were performed at room temperature.

### Calcium imaging

Fura-2 calcium imaging on monolayer hCO cells was performed as previously described^26^. In brief, cells were loaded with 1 mM Fura-2 acetoxymethyl ester (Fura-2 AM, Invitrogen, no. F1221) for 30 min at 37 °C in NM medium, washed with NM medium for 5 min and then transferred to a perfusion chamber (RC-20, Warner instruments) in low-potassium Tyrode’s solution (5 mM KCl, 129 mM NaCl, 2 mM CaCl_2_, 1 mM MgCl_2_, 30 mM glucose, 25 mM HEPES pH 7.4) on the stage of an inverted fluorescence microscope (Eclipse TE2000U, Nikon). Following 0.5 min of baseline imaging, high-potassium Tyrode’s solution was perfused for 1 min. Imaging was performed at room temperature (25 °C) on an epifluorescence microscope equipped with an excitation filter wheel and an automated stage. Openlab software (PerkinElmer) and IGOR Pro (v.5.1, WaveMetrics) were used to collect and quantify time-lapse excitation 340:380-nm-ratio images at an imaging rate of approximately 1 Hz, as previously described^20^. Residual calcium was calculated as (*C* − *A*)/(*B* − *A*), where *A* is the baseline value (fifth frame), *B* is the peak value following depolarization (manually determined) and *C* is the decay value (*B* + 25th frame).

For GCaMP imaging, HEK293T cells were seeded in 24-well plates. The following day, cells were transfected with a mixture of plasmids including subunits Ca_V_1.2 β1b, α2δ and α1 and GCaMP6-X (Supplementary Table 3). Three days post transfection, imaging was performed with an SP8 confocal microscope (Leica Microsystems) at a frame interval of 1.2875 s. Before imaging, cell culture medium was replaced with 500 μl of 5 mM Tyrode’s solution. Following 30 s of baseline imaging, 500 μl of 129 mM Tyrode’s solution (final concentration 67 mM KCl) was added.

Similarly, for GCaMP imaging in two-dimensional neurons, TS and WT hCO were dissociated into 24-well imaging plates (Cellvis P24-0-N) and infected with AAV-DJ-hSYN1::GCaMP6f (Gene Vector and Virus Core, Wu Tsai Neurosciences Institute, Stanford University). Various concentrations of ASOs (ASO.14, ASO.17, ASO.18 and ASO.Scr) were applied to dissociated neurons. After 10 days, GCaMP imaging was carried out with an SP8 confocal microscope using the 20× objective at 1.2875 s per frame). Before imaging, culture medium was replaced with 500 μl of 5 mM Tyrode’s solution. Following 30 s of baseline imaging, 500 μl of 129 mM Tyrode’s solution (final concentration 67 mM KCl) was added. Imaging was acquired over a total time of 8 min.

For GCaMP imaging analysis of HEK293T cells, regions of interest (ROIs) corresponding to cell somas were identified semiautomatically using a custom-written ImageJ segmentation macro. ROIs were detected in the frame following depolarization (fifth or sixth frame following KCl administration) by applying a mask, watershedding and using the ‘Analyze particles’ function (size 10–1,000, circularity 0.4–1.0). A minority of ROIs were manually excluded due to either cell drift, off-target detection of background or detection of more than a single soma within the same ROI. For GCaMP analysis in neurons, ROIs corresponding to cell somas were manually annotated. Downstream analyses for both HEK293T cells and neurons were performed using custom-written R codes. Mean grey values were transformed to relative changes in fluorescence: d*F*/*F*(*t*) = (*F*(*t*) − *F*_0_)/*F*_0_, where *F*_0_ represents average grey values of the time series of each ROI. Cells were excluded if their amplitude was lower than the baseline mean or more than 20× baseline mean. Residual calcium values were calculated as described above, with *B* representing peak value, *A* baseline value (20 frames upstream of the peak-value frame) and *C* decay value (200 frames after the peak-value frame). Extreme residual calcium values (lower than −5 or higher than +5) were excluded.

### Patch-clamp recordings

Patch-clamp recordings were performed on cortical neurons dissociated from hCO, as previously described^4^. hCO were dissociated at days 100–150. A few days following dissociation, cells were infected with AAV-DJ-SYN1::eYFP and 1 μM ASO was added 1 week following dissociation. Recordings were typically made around 3–4 weeks following dissociation. Cells were identified as eYFP^+^ with an upright slice scope microscope (Scientifica) equipped with an Infinity2 CCD camera and Infinity Capture software (Teledyne Lumenera). Recordings were performed with borosilicate glass electrodes with a resistance of 7–10 MΩ. For barium current recordings the external solution contained 100 mM NaCl, 3 mM KCl, 2 mM MgCl_2_, 20 mM BaCl_2_, 25 mM TEA-Cl, 4 mM 4-AP, 10 mM HEPES and 20 mM glucose pH 7.4, with NaOH and 300 mOsm. The internal solution contained 110 mM CsMethylSO_3_, 30 mM TEA-Cl, 10 mM EGTA, 4 mM MgATP, 0.3 mM Na_2_GTP, 10 mM HEPES and 5 mM QX314-Cl pH 7.2, with CsOH and 290 mOsm. Data were acquired with a MultiClamp 700B Amplifier (Molecular Devices) and a Digidata 1550B Digitizer (Molecular Devices), low-pass filtered at 2 kHz, digitized at 20 kHz and analysed with pCLAMP (v.10.6, Molecular Devices). Cells were subjected to −10 mV hyperpolarization (100 ms) every 10 s to monitor input and access resistance. Cells were excluded for analysis if they showed a change of over 30%. Liquid junction potential was not corrected in this study.

For barium current recordings, cells were recorded in the presence of tetrodotoxin (TTX) (0.5 μM) to block sodium currents and were held at −70 mV in voltage-clamp and depolarizing voltage steps (5 s for the majority of cells, from −70 to +20 mV) in increments of 5 mV. Inactivation of barium current was calculated from cells subjected to 5 s or 2–3-s depolarization steps at 2 s under maximal current (−20 to 0 mV for the majority). For some cells, recordings with a prestep of −110 mV (or −100 mV) hyperpolarization were also included for inactivation at 2 s. Leak subtraction was used to minimize the artefact of membrane resistance in MultiClamp 700B. *I*–*V* curves were fitted in Origin (OriginPro 2021b, OriginLab) with a Boltzmann exponential function: *I* = *G*_max_ × (*V* − *E*_Ba_)/{1 + exp[(*V*_0.5_ − *V*)/*K*]}, where *G*_max_ is the maximal conductance of calcium channels, *E*_Ba_ is the reversal potential of barium estimated by the curve-fitting programme, *V*_0.5_ is the potential for half-maximal, steady-state activation of barium current and *K* is a voltage-dependent slope factor.

For voltage-dependent barium current inactivation, cells were held at −70 mV. A series of prepulse voltage steps (3 s) were administered, from −110 or −100 to +40 mV, in increments of 10 mV. Testing of the voltage step (−10 or 0 mV, where maximal current was recorded) was then carried out for a further 1–3 s. Barium current inactivation was calculated as relative current normalized to current amplitude from the first test pulse. Voltage-dependent inactivation curves were fitted with exponential functions in Origin.

### Immunostaining

Dissociated cells from TS hCO at 100–120 days of differentiation were plated on precoated coverslips and placed in wells of a 12-well plate; different concentrations of Cy5-ASO.14 were then added. After 3 days the coverslips were first fixed for 10 min at room temperature with a solution containing one volume each of culture medium and fixation buffer comprising 4% paraformaldehyde (PFA) and 4% sucrose in DPBS. Next, two volumes of fixation buffer were added for an extra 20 min to finalize the fixation step. Following two rounds of washing with DPBS, coverslips were incubated for 1 h with blocking buffer consisting of 0.3% Triton X-100 and 10% normal donkey serum prepared with PBS. Following removal of the blocking buffer, primary antibodies were added for overnight incubation at 4 °C. Antibodies CTIP2 (abcam, catalogue no. ab18465) and SATB2 (abcam, catalogue no. ab51502) were diluted in blocking buffer at 1:300. Coverslips were washed twice with DPBS then incubated with secondary antibody (1:1,000 in blocking buffer; donkey anti-rat Alexa 488, Thermo Fisher Scientific, catalogue no. A-21208; and donkey anti-mouse Alexa 568, Thermo Fisher Scientific, catalogue no. A10037) at room temperature for 1 h. Following a further two rounds of washing with DPBS, Hoechst 33258 (Thermo Fisher Scientific, catalogue no. H3569) was added to coverslips for 10 min followed by a final round of washing with DPBS. Finally, coverslips were mounted on slides (Fisherbrand Superfrost Plus Microscope Slides, Fisher Scientific, catalogue no. 12-550-15) using Aqua-Poly/Mount (Polysciences, catalogue no. 18606). Images were acquired with a confocal SP8 (Leica Microsystems) using a 20× objective.

The TUNEL assay was performed using the in situ cell death detection kit (Roche, catalogue no. 12156792910). In brief, hCO were dissociated and exposed to either 1 μM ASO or scrambled control for 48 h. Cells were then fixed in 4% PFA, permeabilized in Triton X-100 and incubated with TUNEL reaction solution for 1 h at 37 °C. Samples pretreated with DNase1 for 10 min were used as positive control. Following rinsing and counterstaining with Hoechst, coverslips were imaged with a Stellaris microscope using the 20× objective. Images were stitched in Fiji and a custom macro was used to split channels, set thresholds for detection of nuclei via Hoechst and determine Cy3^+^ nuclei via thresholds set blindly on control samples.

For c-Cas3, immunostaining was performed as for Cy5 samples except that rabbit anti-c-Cas3 (Asp175) (1:300, CST, catalogue no. 9661S) and mouse anti-MAP2 antibody (1:100, Sigma-Aldrich, catalogue no. M4403) were used as primary antibodies and donkey anti-rabbit 568 (1:1,000, Thermo Fisher Scientific, catalogue no. A10042) and donkey anti-mouse Alexa:568 (1:1,000, Thermofisher Scientific, catalogue no. A10037) as secondary antibodies. Coverslips were imaged with a confocal SP8 microscope using the 40× objective. Three to four fields were acquired per coverslip. Images were analysed using Fiji with maximal projection, standardized thresholding and circularization to identify cells (via Hoechst nuclear staining) and then c-Cas3^+^ cells (via Cas3 staining).

For staining of t-hCO, following slicing of fresh rat brain containing t-hCO, slices were postfixed in 4% PFA overnight at 4 °C and then washed three times with PBS. Next, slices were incubated with blocking buffer at room temperature for 1 h with 10% normal donkey serum and 0.3% (vol/vol) Triton X-100 in DPBS then incubated with primary antibody diluted in blocking buffer overnight at 4 °C (anti-HNA, mouse, 1:200, abcam, catalogue no. ab191181). Washing steps, staining with secondary antibody and staining of nuclei are described above.

### Flow cytometry

TS hCO were incubated with 1 μM Cy5.ASO.14 in wells of 24-well, ultralow-attachment plate (Corning, catalogue no. 3473) for 2 days. hCO were then dissociated and resuspended in 200 μl of staining buffer containing 3% bovine serum albumin and 0.5 mM EDTA. Cells were incubated either with or without PE Mouse Anti-Human CD90 (BD Biosciences, catalogue no. 555596, dilution 1:100) for 30 min at 4 °C. Next, three rounds of washing steps were performed using the staining buffer and cells were resuspended in 200 μl of staining buffer and passed through a 40 μm cell strainer. Non-treated hCO not stained with CD90 served as a control for setting up the gate during cell acquisition. G575 and R670 were used for measurement of PE and Cy5 signal, respectively. Flow cytometry was performed on a BD Aria cell sorter at the Stanford Shared FACS Facility according to the Facility’s calibration instructions. Data were processed using FlowJo 10.7.1 software (BD).

### Immunoblot for measurement of Ca_V_1.2 protein level

hCO derived from control and TS iPS cell lines were aliquoted to wells of a 24-well, ultralow-attachment plate (Corning, catalogue no. 3473). Each well contained two or three hCO cultured in 2 ml of neural medium, followed by the addition of 1 μM ASO. Medium was 50% replaced following 3 days of ASO exposure and samples collected following 7 days of ASO exposure. Protein lysates for hCO were prepared using the RIPA buffer system (Santa Cruz, catalogue no. sc-24948). Protein lysates of t-hCO were prepared by the brief addition of 50 µl of SDS Buffer (1.5% SDS, 25 mM Tris pH 7.5) in a 1.5 ml tube followed by sonication (Qsonica Q500 sonicator; pulse 3 s on, 3 s off, amplitude 20%). Protein concentrations were quantified using the bicinchoninic acid assay (Pierce, ThermoFisher, catalogue no. 23225): 20 μg of protein per sample per lane was loaded and run on a 4–12% Bis-Tris PAGE gel (Bolt 4–12% Bis-Tris Protein Gel, Invitrogen, no. NW04122BOX) and transferred to a polyvinylidene difluoride membrane (Immobulin-FL, EMD Millipore, catalogue no. IPFL00010). Membranes were blocked with 5% bovine serum albumin in Tris buffered saline with Tween (TBS-T) for 1 h at room temperature and incubated overnight with primary antibodies against GAPDH (mouse, 1:5,000, GeneTex, catalogue no. GTX627408) and Ca_V_1.2 (rabbit, 1:1,000, Alamone labs, catalogue no. ACC-003) for 48 h for hCO samples, and for 96 h for transplanted samples, at 4 °C. Membranes were washed three times with TBS-T and then incubated with near-infrared fluorophore-conjugated species-specific secondary antibodies—either goat anti-mouse IgG polyclonal antibody (IRDye 680RD, 1:10,000, LI-COR Biosciences, catalogue no. 926–68070) or goat anti-rabbit IgG polyclonal antibody (IRDye 800CW, 1:10,000, LI-COR Biosciences, catalogue no. 926–32211), for 1 h at room temperature. Following the application of secondary antibody, membranes were washed three times with TBS-T, once with TBS and then imaged using a LI-COR Odyssey CLx imaging system (LI-COR).

### TLR9 assay for ASO toxicity

We used the human TLR9 reporter assay (Invivogen, catalogue no. hkb-htlr9) according to the manufacturer’s instructions. In brief, modified HEK293T cells were grown on 100 mm cell culture plates to 50–80% confluency. They were then detached in PBS, resuspended at 450,000 cells ml^−1^ in HEKBlue solution and replated into a 96-well plate. Positive controls were exposed to ODN2006 (Invivogen, catalogue no. tlrl-2006), and negative controls to sterile water; other samples were exposed to 1 μM ASO for 16–24 h. Following exposure, TLR9 activation was detected by spectrophotometer (620–655 nm absorption) using a monochromator plate reader (Tecan, Infinite M1000) and XFluor 2.0 software.

### Interneuron migration and imaging analysis

Following 45–50 days of differentiation, hSO were incubated overnight with LV.Dlxi1/2b::eGFP lentiviral particles in an Eppendorf tube and transferred to a 24-well plate. After 3–7 days, hSO were coincubated with an hCO in an Eppendorf tube supplemented with 1 ml of medium to generate hFA, which were then cultured in a single well of an ultralow-attachment 24-well plate (Corning). Baseline imaging of interneuron migration was taken around 3–4 weeks following the formation of hFA. Next, 1 μM ASO was added to hFA followed by reimaging 2 weeks later. All imaging was taken over a period of 20 min for 12–15 h inside a confocal chamber at 37 °C in a humidified-air atmosphere with 5% CO_2_. Quantification of saltation length and frequency was performed as previously described^3^. Only mobile cells were included for analysis. ImageJ was used for analysis of interneuron migration. In cases where hFA moved during imaging, linear stack alignment with SIFT was used to correct minor shifts. To estimate the distance of individual saltations, Dlxi1/2b::eGFP cells showing a swelling of the soma were identified and distance (in μm) to the new position of the soma following nucleokinesis was recorded manually. The time necessary for this movement was used to calculate the speed when mobile. Typically, only cells showing two or more saltation movements were included.

### Transplantation into athymic newborn rats

Animal procedures were performed following animal care guidelines approved by Stanford University’s Administrative Panel on Laboratory Animal Care (APLAC). Pregnant RNU euthymic (rnu/^+^) rats were either purchased (Charles River Laboratories) or bred in house. Animals were maintained under a 12/12 h light/dark cycle and provided food and water ad libitum. Three-to-seven-day-old athymic (*FOXN1*^−/−^) rat pups were identified by immature whisker growth before culling. Pups (both male and female) were anaesthetized with 2–3% isoflurane and mounted on a stereotaxic frame. A craniotomy, of about 2–3 mm in diameter, was performed above S1, preserving the dura intact. Next, the dura mater was punctured using a 30-G needle (approximately 0.3 mm) close to the lateral side of the craniotomy. A hCO was next moved onto a thin, 3 × 3 cm parafilm and excess medium removed. Using a Hamilton syringe connected to a 23-G, 45° needle, the hCO was gently pulled into the distal tip of the needle. The syringe was next mounted on a syringe pump connected to the stereotaxic device. The sharp tip of the needle was positioned above the 0.3-mm-wide prefabricated puncture in the dura mater (*z* = 0 mm) and the syringe was reduced by 1–2 mm (*z* = approximately −1.5 mm) until a tight seal between needle and dura mater had formed. Next, the syringe was elevated to the centre of the cortical surface at *z* = −0.5 mm and the hCO ejected at a speed of 1–2 μl min^−1^. Following completion of hCo injection, the needle was retracted at a rate of 0.2–0.5 mm min^−1^, the skin was closed and the pup immediately placed on a warm heat pad until complete recovery.

### MRI of transplanted rats

All animal procedures followed animal care guidelines approved by Stanford University’s APLAC. Rats (more than 60 days post transplantation) were anaesthetized with 5% isoflurane for induction and 1–3% isoflurane during imaging. For imaging, an actively shielded Bruker 7 Tesla horizontal bore scanner (Bruker Corp.) with International Electric Company gradient drivers, a 120-mm-inner-diameter shielded gradient insert (600 mT m^−1^, 1,000 T m^−1^ s^−1^), AVANCE III electronics, eight-channel multicoil radiofrequency and multinuclear capabilities, and the supporting Paravision 6.0.1 platform, were used. Acquisitions were performed with an 86-mm-inner-diameter actively decouplable volume radiofrequency coil with a four-channel, cryocooled, receive-only radiofrequency coil. Axial two-dimensional Turbo-RARE (TR 2,500 ms, TE 33 ms, two averages) 16-slice acquisitions were performed at 0.6–0.8 mm slice thickness with samples of approximately 256 Å. Signal was received by a 2-cm-inner-diameter quadrature transmit–receive volume radiofrequency coil (Rapid MR International). Successful transplantations were defined as those resulting in a continuous area of T2-weighted MRI signal in the transplanted hemisphere.

### ASO injection into rat cisterna magna

Rats were anaesthetized with 5% isoflurane for induction and 2–3% isoflurane during ASO injection through the cisterna magna. Animals were placed in the prone position with a small paper roll under the neck to tilt the head downwards. The neck was shaved and wiped clean with ethanol. To target the cisterna magna the foramen magnum was determined by touch and a 27-G needle attached to a syringe (BD, catalogue no. 305620) filled with 300 μg of ASO was percutaneously inserted into the cisterna magna perpendicularly to the neck. The needle was held with the bevel face upwards and 30 μl of ASO was slowly injected into the cisterna magna. The procedure took less than 2 min per rat. Animals recovered from anaesthesia within 10 min of isoflurane induction. ASO injections were performed in rats with t-hCO at 162–258 days and were not blinded. Sample sizes were estimated empirically.

### Processing of ASO-injected rats

Rats were anaesthetized with isoflurane and brain tissue was removed and placed in cold (approximately 4 °C), oxygenated (95% O_2_ and 5% CO_2_) sucrose slicing solution containing 234 mM sucrose, 11 mM glucose, 26 mM NaHCO_3_, 2.5 mM KCl, 1.25 mM NaH_2_PO_4_, 10 mM MgSO_4_ and 0.5 mM CaCl_2_ (approximately 310 mOsm). Coronal rat brain slices (300–400 μm) containing t-hCO were sectioned using a Leica VT1200 vibratome as previously described^3^. t-hCO sections were then moved to a continuously oxygenated slice chamber, at room temperature, which contained aCSF (10 mM glucose, 26 mM NaHCO_3_, 2.5 mM KCl, 1.25 mM NaHPO_4_, 1 mM MgSO_4_, 2 mM CaCl_2_ and 126 mM NaCl (298 mOsm)).

### Calcium imaging in t-hCO from rats receiving ASO injection

Following dissection and sectioning of rat brains with t-hCO, slices were incubated with Calbryte 520 AM (AAT Bioquest, catalogue no. 20650) in 1:1 of NPC medium and PBS for 45–60 min at 37 °C. Slices were then transferred to a 24-well imaging plate containing 500 μl of warm, low-potassium Tyrode’s solution (5 mM KCl, 129 mM NaCl, 2 mM CaCl_2_, 1 mM MgCl_2_, 30 mM glucose, 25 mM HEPES pH 7.4) and imaged with a confocal microscope (Leica Stellaris) for 30 s at 37 °C, after which medium was replaced by high-potassium Tyrode’s solution (high-KCl, 67 mM KCl: 67 mM NaCl, 2 mM CaCl_2_, 1 mM MgCl_2_, 30 mM glucose and 25 mM HEPES pH 7.4) and imaging resumed for 25 min. Mean grey values were collected from ROIs delineating Calbryte^+^ somas (visualized by standard deviation projection of the entire time series) with Fiji (ImageJ v.2.1.0, NIH). Mean grey values were transformed to relative changes in fluorescence: d*F*/*F*(*t*) = (*F*(*t*) − *F*_0_)/*F*_0_, where *F*_0_ represents average grey values of the time series of each ROI. Residual calcium was calculated as (*C* − *A*)/(*B* − *A*), where *B* is the peak value following depolarization (maximal peak value determined by custom-written MATLAB routines (v. R2019b and v. R2022b, 9.4.0, MathWorks), *A* is the baseline value (*B* − 50th frame) and *C* is the decay value (*B* + 150th frame).

### Golgi staining

Golgi staining was conducted using the FD Rapid GolgiStain Kit (FD Neurotechnologies, catalogue no. PK401) according to the manufacturer’s instructions. In brief, freshly dissected t-hCO were incubated with solution A/B mixture in the dark and then transferred to solution C. After 72 h the tissue was embedded in agarose, the vibratome chamber filled with solution C and tissue sectioned at 100 μm using a Leica VT1200S vibratome. Sections were mounted on gelatin-coated slides, stained in solution D/E, washed, dehydrated, cleared and coverslipped. Images were acquired on a SP8 confocal microscope with brightfield. Cells were counted as neurons based on their morphology; dendrites were manually traced using neuTube. Both tracing and analysis were performed blinded.

### Statistics and reproducibility

Data are presented as either mean ± s.d. or mean ± s.e.m. unless otherwise indicated. Distribution of raw data was tested for normality of distribution; statistical analyses were performed using either two-tailed student’s *t*-tests, one-way ANOVA with multiple comparisons, two-tailed Mann–Whitney tests or Kruskal–Wallis tests. Statistical analysis was performed in Prism (GraphPad). Data shown for representative experiments were repeated, with similar results, in at least three independent biological replicates, unless otherwise noted. Sample sizes were estimated empirically.

### Reporting summary

Further information on research design is available in the Nature Portfolio Reporting Summary linked to this article.

## Online content

Any methods, additional references, Nature Portfolio reporting summaries, source data, extended data, supplementary information, acknowledgements, peer review information; details of author contributions and competing interests; and statements of data and code availability are available at 10.1038/s41586-024-07310-6.

### Supplementary information

Supplementary Information

Reporting Summary

### Source data

Source Data Fig. 1.

Source Data Fig. 2.

Source Data Fig. 3.

Source Data Fig. 4.

Source Data Fig. 5.

Source Data Extended Data Fig. 1.

Source Data Extended Data Fig. 2.

Source Data Extended Data Fig. 3.

Source Data Extended Data Fig. 4.

Source Data Extended Data Fig. 5.

Source Data Extended Data Fig. 6.

Source Data Extended Data Fig. 7.

Source Data Extended Data Fig. 8.

Source Data Extended Data Fig. 9.

Source Data Extended Data Fig. 10.

## Data Availability

The data that support the findings of this study are available on request from the corresponding author. The following public datasets were used to support this study: the Gene Expression in Cortical Organoids (GECO) database^26^ (http://solo.bmap.ucla.edu/shiny/GECO/) and Evo-devo: Alternative splicing database^25^ (https://apps.kaessmannlab.org/alternative-splicing/). Source data are provided with this paper.

## Code availability

The code for calcium imaging analysis is available in Supplementary Notes.
