# Supplementary material for: Antisense oligonucleotide therapeutic approach for Timothy syndrome
Source: Nature. 2024 Apr 24;628(8009):818–25. doi: 10.1038/s41586-024-07310-6 (PMC11043036; doi:10.1038/s41586-024-07310-6)
Supplement: Supplementary file 1 — Supplementary Information [file 41586_2024_7310_MOESM1_ESM.pdf]

nature portfolio

https://doi.org/10.1038/s41586-024-07310-6

Supplementary information

Antisense oligonucleotide therapeutic approach for Timothy syndrome

In the format provided by the authors and unedited

Antisense oligonucleotide therapeutic approach for Timothy syndrome

Xiaoyu Chen[1,2#], Fikri Birey[1,2,5#], Min-Yin Li[1,2], Omer Revah[1,2,6], Rebecca Levy[3], Mayuri Vijay Thete[1,2], Noah Reis[1,2], Konstantin Kaganovsky[1,2], Massimo Onesto[1,2], Noriaki Sakai[1], Zuzana Hudacova[1], Jin Hao[1,2], Xiangling Meng[1,2], Seiji Nishino[1], John Huguenard[4], and Sergiu P. Pașca[1,2*]

[1]Department of Psychiatry and Behavioral Sciences, Stanford University, Stanford, CA, USA
[2]Stanford Brain Organogenesis, Wu Tsai Neurosciences Institute & Bio-X, Stanford University, Stanford, CA, USA
[3]Department of Neurology, Division of Child Neurology, Stanford University, Stanford, CA, USA
[4]Department of Neurology and Neurological Sciences, Stanford University, Stanford, CA, USA
[5]Current address: Department of Human Genetics, Emory University, Atlanta, GA, USA

[#]These authors contributed equally to this study
*Corresponding author: spasca@stanford.edu

Supplementary Table 1. Primer sequences used in this study.

Supplementary Table 2. PCR cycling conditions.

Supplementary Table 3. HEK cells transfection scheme used for minigene splicing reporters.

Supplementary Table 4. HEK cells transfection scheme used for studying the role of PTBP1 on exon 8A and exon 8 splicing.

Supplementary Table 5. HEK cells transfection scheme used for GCaMP imaging shown in **Extended Data Fig. 8**.

Supplementary Figure 1
Gating strategy used for the flow cytometry data presented in **Fig. 2**
hCO not treated (**a**) and treated with Cy5-ASO (**b**) were dissociated and stained with the neuronal cell surface protein CD90.

Supplementary Figure 2
Full Western blot images for the data shown in **Extended Data Fig. 10b**

Supplementary Figure 3 Plasmid map and sequence of the WT minigene splicing reporter.

Supplementary Figure 4 Plasmid map and sequence of the TS minigene splicing reporter.

Supplementary Figure 5 Plasmid map and sequence of over expression plasmids of human WT *CACNA1C*.

Supplementary Figure 6 Plasmid map and sequence of over expression plasmids of human *CACNA1C* containing TS mutation.

Supplementary Notes
Source code in R for analyzing the GCaMP imaging shown **Extended Data Fig. 8**

Supplementary Table 1

Name	Squence	Purpose
NGS.exon7-9.S.2	ACACTCTTTCCCTACACGACGCTCTTCCGATCTacaactttgccttcgccatg	NGS
NGS.exon7-9.AS.2	GACTGGAGTTCAGACGTGTGCTCTTCCGATCTgtcttcggcctgagtgatcc	
mini.NGS.S.2	ACACTCTTTCCCTACACGACGCTCTTCCGATCTatgaagttggtggtgaggcc	
mini.NGS.AS.2	GACTGGAGTTCAGACGTGTGCTCTTCCGATCTaggagtggacagatccccaa	
GAPDH.S	CATGAGAAGTATGACAACAGCCT	qPCR-human
GAPDH.AS	AGTCCTTCCACGATACCAAAGT	
qPCR.exon8.S	ACGCTATGGGCTATGAGTTACC	
qPCR.exon8.AS	GGCCTTCTCCCTCTCTTTG	
qPCR.exon8A.S	TTTGACAACTTTGCCTTCGC	
qPCR.exon8A.AS	TCCCTTCCTACGGCATCATT	
Rat.Gapdh.S	CAACTCCCTCAAGATTGTCAGCAA	qPCR-rat
Rat.Gapdh.AS	GGCATGGACTGTGGTCATGA	
Rat.Exon8.S.1	ACGCTATGGGCTATGAGTTGCC	
Rat.Exon8.AS.1	GGCTTTCTCCCTCTCTTTG	
Rat.Exon8a.S	TTCGACAACTTCGCCTTCGC	
Rat.Exon8a.AS.1	TCCCTTCCTACGGCATCATT	
1.Minigene.AS.bgiii	TGCCCAGATCTCTCCTCCATTGTCCACCACC	Cloning of minisplicing reporter
1.Minigene.S.apa	TGATAGGGCCCGCGGGCCGAGGTCCCCTTCG	
CA.RT.S2	GATGACCCTTCCCCTTGTGC	RFLP
CA.RT.AS2	CATCCATGCCTTCGTCCTCA	
PCR.mini.S.1	AGAAGTCTGCCGTTACTGCC	Minisplicing reporter RT-PCR
PCR.mini.AS.3	AACCTCTGGGTCCAAGGGTA	
DGKK.S	AGAAGAGATGAACACCCAGGGCAA	Off-target assessment
DGKK.AS	GCAGGTTTGGCAAGGAGATGGTTT	
TMEM105.S	TGGCAGCAGGGATAACAG	
TMEM105.AS	TGAGCAACAGAGCAAGACT	
Cav1.3-F	CTTCGACAACGTCCTCTCTGCT	
Cav1.3-R	GCCGATGTTCTCTCCATTCGAG	
USP28.S	ACTCAGACTATTGAACAGATGTACTGC	
USP28.AS	CTGCATGCAAGCGATAAGG	

Supplementary Table 2

Purpose	PCR cycling condition	Polymerase
NGS	98°C 2min	Phire Tissue Direct PCR Master Mix
	98°C 7s, 65°C 7s, 72°C 20s for 40 cycles	
	72°C 1min	
Cloning of minigene Splicing reporter	95°C 2min	GoTaq Long PCR Master Mix
	92°C 30s, 65°C 1min for 35 cycles	
	72°C 10min	
RFLP	98°C 2min	Phire Tissue Direct PCR Master Mix
	98°C 7s, 66°C 7s, 72°C 20s for 40 cycles	
	72°C 1min	
Minisplicing reporter RT-PCR	98°C 2min	Phire Tissue Direct PCR Master Mix
	98°C 7s, 68°C 7s, 72°C 20s for 40 cycles	
	72°C 1min	

Supplementary Table 3

	24-well plate	
HEK 293T Cells	30,000-75,000 cells/well (1.65 μl PEI per well, medium replaced at 24 h post transfection)	
Plasmids	pDup.8-8a^{WT}	pDup.8-8a^{TS}
DNA (ng)		600
	600	

Supplementary Table 4

	24-well plate		
HEK 293T cells	30,000-75,000 cells/well (1.65 μl PEI per well, medium replaced at 24 h post transfection)		
	pDup.8-8a^{WT}	pDup.8-8a^{TS}	pPTBP1
Plasmids DNA (ng)	300		
	300		300
		300	
		300	300

Supplementary Table 5

	24-well plate				
HEK 293T cells	30,000-75,000 cells/well (1.65 μl PEI per well, medium replaced at 24 h post transfection)				
	pLV.CAG.CACNA1C	pLV.CAG.CACNA1C.TS	pCMV.GCaMP6	pβ1b	pα2δ
Plasmids DNA (ng)	400	0	100	200	200
	390	10	100	200	200
	380	20	100	200	200
	370	30	100	200	200
	360	40	100	200	200
	350	50	100	200	200
	340	60	100	200	200
	330	70	100	200	200
	320	80	100	200	200
	310	90	100	200	200
	300	100	100	200	200
	0	400	100	200	200

Supplementary Figure 1

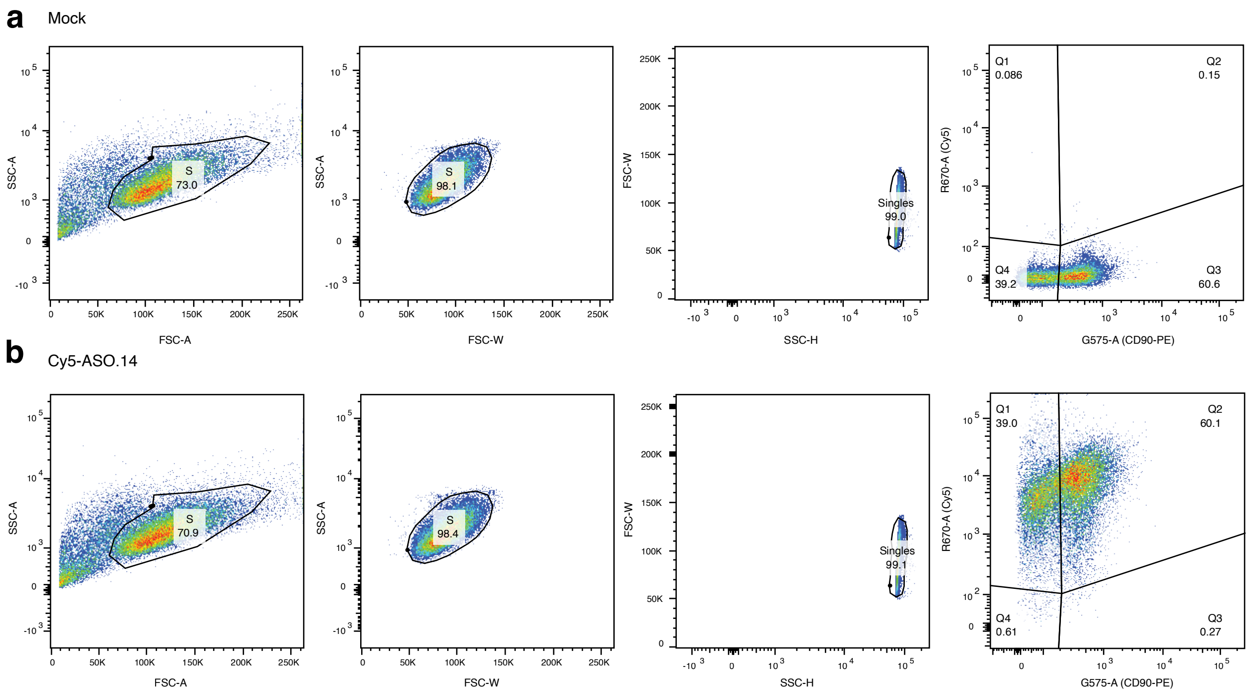

Supplementary Figure 2

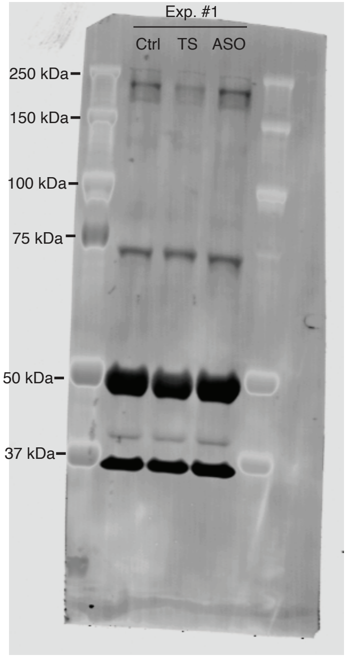

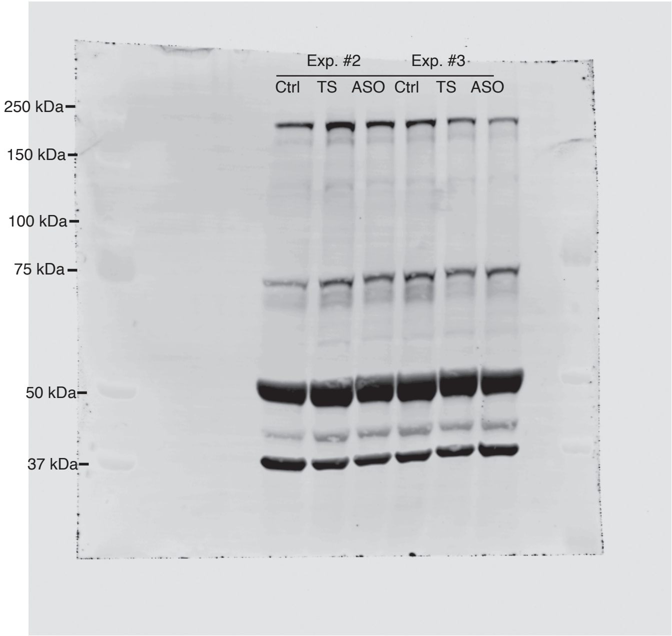

Supplementary Figure 3

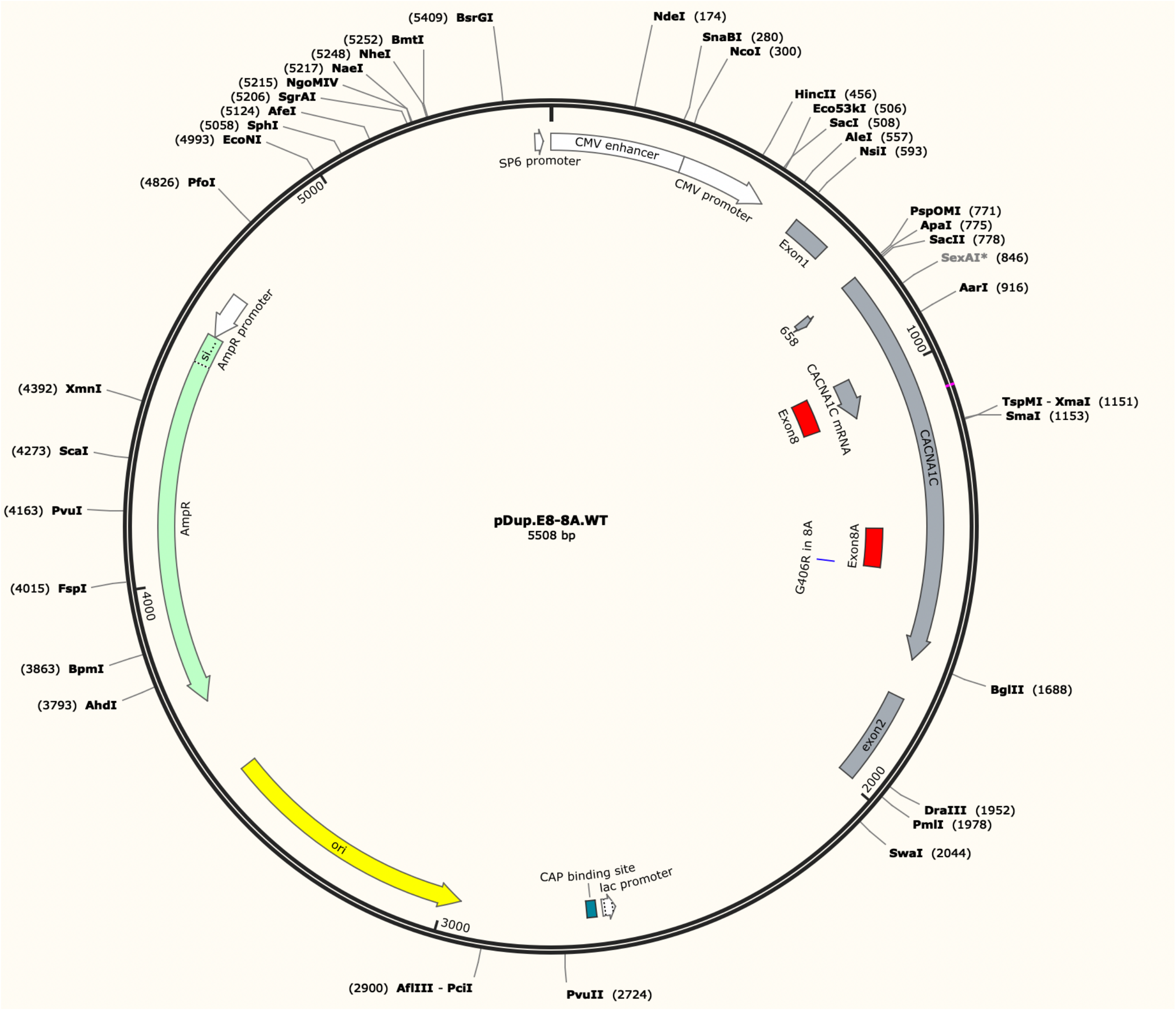

CGTTACATAACTTACGGTAAATGGCCCGCCTGGCTGACCGCCCAACGACCCCCGCCCATTGACGTCAATAATGACGTATGTTCCCATAGTAACGCCAATAGGGACTTT
CCATTGACGTCAATGGGTGGAGTATTTACGGTAAACTGCCCACTTGGCAGTACATCAAGTGTATCATATGCCAAGTCCGCCCCCTATTGACGTCAATGACGGTAAATG
GCCCGCCTGGCATTATGCCCAGTACATGACCTTACGGGACTTTCCTACTTGGCAGTACATCTACGTATTAGTCATCGCTATTACCATGGTGATGCGGTTTTGGCAGTAC
ACCAATGGGCGTGGATAGCGGTTTGACTCACGGGGATTTCCAAGTCTCCACCCCATTGACGTCAATGGGAGTTTGTTTTGGCACCAAAATCAACGGGACTTTCCAA
AATGTCGTAATAACCCCGCCCCGTTGACGCAAATGGGCGGTAGGCGTGTACGGTGGGAGGTCTATATAAGCAGAGCTCGTTTAGTGAACCGTCAGATCAGCTTGCT
TACATTTGCTTCTGACACAACTGTGTTCACTAGCAACCTCAAACAGACACCATGCATGGTGCACCTGACTCCTGAGGAGAAGTCTGCCGTTACTGCCCTGTGGGGCA
AGGTGAACGTGGATGAAGTTGGTGGTGAGGCCCTGGGCAGGTTGGTATCAAGGTTACAAGACAGGTTTAAGGAGACCAATAGAAACTGGGCATGTGGAGACAG
AGAAGACTCTTGGGTTTCTGATAGGGCCCGCGGGCCGAGGTCCCCTTCGGTCACAGGAGTTCCTTTGAACATGGGCGATGCCCTGGGTAACACGGGTAACCTGGT
GCACATGAACAAAGCCCACGTTCAGCCCCGTCTGTCCCCTCCCAATCTGCTCACACCTGCTGCCTGCCTCTTTGCTGTAACCCAATTCTGCTTCTTCTTTCCTAACTTT
CCTTCGTCTTTCCAGATGCAGGACGCTATGGGCTATGAGTTACCCTGGGTGTATTTTGTCAGTCTGGTCATCTTTGGATCCTTTTTCGTTCTAAATCTGGTTCTCGGTG
TGTTGAGCGGGTAAGCTGACCGTTTCTATGTCCTCTCCACAACGCAGCCGAGCAAGGTCTCAGGTTCCACTCCGTACATGCCCGGGGTCCTCAGGGATGGGACCCT
GACAGGCCCAGGAAAACCACAACAAAGCCTCTGTTCAACCACAGATTCTGACCCATTGGCCAGGCAGGCTGTTTGGCCTCTGATTTGCACCTAGAGGGTCCCCGG
ATCCTGGCGCTGCGTGGGTCAGTGTCTCGGGAGCCGGGGACCGGCACTGGCCGTGCTCGGTTGCTGAGTGTGCCTCACTAACTATCATTCCGTTCTTCCAGGTCAA
TGATGCCGTAGGAAGGGACTGGCCCTGGATCTATTTTGTTACACTAATCATCATAGGGTCATTTTTTGTACTTAACTTGGTTCTCGGTGTGCTTAGCGGGTAAGCAGG
ACCAAGGAAAAAGGTCTTGATTTTTCCATTTATTTTTATTTATTCTTTCTGCTATTCCTGGCTGTATTCTTTTTCTGGCTCTGATGAACCTGGGATAAGGGGTCACCACA
GGAGCCTTGAAGTGGATGTCCTTGTCCTGGCTGGGTAAAGGGTCAGATGTGCCACTGGTCTTGGTGGTGGACAATGGAGGAGAGATCTGGGCATGTGGAGACAG
AGAAGACTCTTGGGTTTCTGATAGGCACTGACTCTCTCTGCCTATTGGTCTATTTTCCCACCCTTAGGCTGCTGGTGGTCTACCCTTGGACCCAGAGGTTCTTTGAGT
CCTTTGGGGATCTGTCCACTCCTGATGCTGTTATGGGCAACCCTAAGGTGAAGGCTCATGGCAAGAAAGTGCTCGGTGCCTTTAGTGATGGCCTGGCTCACCTGGA
CAACCTCAAGGGCACCTTTGCCACACTGAGTGAGCTGCACTGTGACAAGCTGCACGTGGATCCTTGAGCATCTGGATTCTGCCTAATAAAAAACATTTATTTTCATT
GCAATGATGTATTTAAATTATTTCTGAATATTTTACTAAAAAGGGAATGTGGGAGGTCAGTGCATTTAAAACATAAAGAAATGAAGAGCTAGTTCAAACCTTGGGAA

AATACACTATATCTTAAACTCCATGAAAGAAGGTGAGGCTGCAAACAGCTAATGCACATTGGCAACAGCCCTGATGCCTATGCCTTATTCATCCCTCAGAAAAGGATT
CAAGTAGAGGCTTGATTTGGAGGTTAAAGTTTTGCTATGCTGTATTTTACATTACTTATTGTTTTAGCTGTCCTCATGAATGTCTTTTCACTACCCATTTGCTTATCCTGC
ATCTCTCAGCCTTGACTCCACTCAGTTCTCTTGCTTAGAGATACCACCTTTCCCCTGAAGTGTTCCTTCCATGTTTTACGGCGAGATGGTTTCTCCTCGCCTGGCCACT
CAGCCTTAGTTGTCTCTGTTGTCTTATAGAGGTCTACTTGAAGAAGGAAAAACAGGGGGCATGGTTTGACTGTCCTGTGAGCCCTTCTTCCCTGCCTCCCCCACTCA
CAGTGACCCGGAATCATCCGCTCACAATTCCACACAACATACGAGCCGGAAGCATAAAGTGTAAAGCCTGGGGTGCCTAATGAGTGAGCTAACTCACATTAATTGCG
TTGCGCTCACTGCCCGCTTTCCAGTCGGGAAACCTGTCGTGCCAGCTGCATTAATGAATCGGCCAACGCGCGGGGAGAGGCGGTTTGCGTATTGGGCGCTCTTCC
GCTTCCTCGCTCACTGACTCGCTGCGCTCGGTCGTTCGGCTGCGGCGAGCGGTATCAGCTCACTCAAAGGCGGTAATACGGTTATCCACAGAATCAGGGGATAACG
CAGGAAAGAACATGTGAGCAAAAGGCCAGCAAAAGGCCAGGAACCGTAAAAAGGCCGCGTTGCTGGCGTTTTTCCATAGGCTCCGCCCCCCTGACGAGCATCAC
AAAAATCGACGCTCAAGTCAGAGGTGGCGAAACCCGACAGGACTATAAAGATACCAGGCGTTTCCCCCTGGAAGCTCCCTCGTGCGCTCTCCTGTTCCGACCCTGC
CGCTTACCGGATACCTGTCCGCCTTTCTCCCTTCGGGAAGCGTGGCGCTTTCTCATAGCTCACGCTGTAGGTATCTCAGTTCGGTGTAGGTCGTTCGCTCCAAGCTGG
GCTGTGTGCACGAACCCCCCGTTCAGCCCGACCGCTGCGCCTTATCCGGTAACTATCGTCTTGAGTCCAACCCGGTAAGACACGACTTATCGCCACTGGCAGCAGCC
ACTGGTAACAGGATTAGCAGAGCGAGGTATGTAGGCGGTGCTACAGAGTTCTTGAAGTGGTGGCCTAACTACGGCTACACTAGAAGAACAGTATTTGGTATCTGCG
CTCTGCTGAAGCCAGTTACCTTCGGAAAAAGAGTTGGTAGCTCTTGATCCGGCAAACAAACCACCGCTGGTAGCGGTGGTTTTTTTGTTTGCAAGCAGCAGATTAC
GCGCAGAAAAAAAGGATCTCAAGAAGATCCTTTGATCTTTTCTACGGGGTCTGACGCTCAGTGGAACGAAAACTCACGTTAAGGGATTTTGGTCATGAGATTATCA
AAAAGGATCTTCACCTAGATCCTTTTAAATTAAAAATGAAGTTTTAAATCAATCTAAAGTATATATGAGTAAACTTGGTCTGACAGTTACCAATGCTTAATCAGTGAGG
CACCTATCTCAGCGATCTGTCTATTTCGTTCATCCATAGTTGCCTGACTCCCCGTCGTGTAGATAACTACGATACGGGAGGGCTTACCATCTGGCCCCAGTGCTGCAAT
GATACCGCGAGACCCACGCTCACCGGCTCCAGATTTATCAGCAATAAACCAGCCAGCCGGAAGGGCCGAGCGCAGAAGTGGTCCTGCAACTTTATCCGCCTCCATC
CAGTCTATTAATTGTTGCCGGGAAGCTAGAGTAAGTAGTTCGCCAGTTAATAGTTTGCGCAACGTTGTTGCCATTGCTACAGGCATCGTGGTGTCACGCTCGTCGTTT
GGTATGGCTTCATTCAGCTCCGGTTCCCAACGATCAAGGCGAGTTACATGATCCCCCATGTTGTGCAAAAAAGCGGTTAGCTCCTTCGGTCCTCCGATCGTTGTCAG
AAGTAAGTTGGCCGCAGTGTTATCACTCATGGTTATGGCAGCACTGCATAATTCTCTTACTGTCATGCCATCCGTAAGATGCTTTTCTGTGACTGGTGAGTACTCAACC
AAGTCATTCTGAGAATAGTGTATGCGGCGACCGAGTTGCTCTTGCCCGGCGTCAATACGGGATAATACCGCGCCACATAGCAGAACTTTAAAAGTGCTCATCATTGG
AAAACGTTCTTCGGGGCGAAAACTCTCAAGGATCTTACCGCTGTTGAGATCCAGTTCGATGTAACCCACTCGTGCACCCAACTGATCTTCAGCATCTTTTACTTTCAC
CAGCGTTTCTGGGTGAGCAAAAACAGGAAGGCAAAATGCCGCAAAAAAGGGAATAAGGGCGACACGGAAATGTTGAATACTCATACTCTTCCTTTTTCAATATTAT
TGAAGCATTTATCAGGGTTATTGTCTCATGAGCGGATACATATTTGAATGTATTTAGAAAAATAAACAAATAGGGGTTCCGCGCACATTTCCCCGAAAAGTGCCACCT
GACGTCTAAGAAACCATTATTATCATGACATTAACCTATAAAAATAGGCGTATCACGAGGCCCTTTCGTCTCGCGCGTTTCGGTGATGACGGTGAAAACCTCTGACAC
ATGCAGCTCCCGGAGACGGTCACAGCTTGTCTGTAAGCGGATGCCGGGAGCAGACAAGCCCGTCAGGGCGCGTCAGCGGGTGTTGGCGGGTGTCGGGGCTGGC
TTAACTATGCGGCATCAGAGCAGATTGTACTGAGAGTGCACCATTCGACGCTCTCCCTTATGCGACTCCTGCATTAGGAAGCAGCCCAGTAGTAGGTTGAGGCCGTT
GAGCACCGCCGCCGCAAGGAATGGTGCATGCAAGGAGATGGCGCCCAACAGTCCCCCGGCCACGGGGCCTGCCACCATACCCACGCCGAAACAAGCGCTCATGA
GCCCGAAGTGGCGAGCCCGATCTTCCCCATCGGTGATGTCGGCGATATAGGCGCCAGCAACCGCACCTGTGGCGCCGGTGATGCCGGCCACGATGCGTCCGGCGT
AGAGGATCTGGCTAGCGATGACCCTGCTGATTGGTTCGCTGACCATTTCCGGGTGCGGGACGGCGTTACCAGAAACTCAGAAGGTTCGTCCAACCAAACCGACTC
TGACGGCAGTTTACGAGAGAGATGATAGGGTCTGCTTCAGTAAGCCAGATGCTACACAATTAGGCTTGTACATATTGTCGTTAGAACGCGGCTACAATTAATACATAA
CCTTATGTATCATACACATACGATTTAGGTGACACTATAGAATACAAGCTGATCCGTC

Supplementary Figure 4

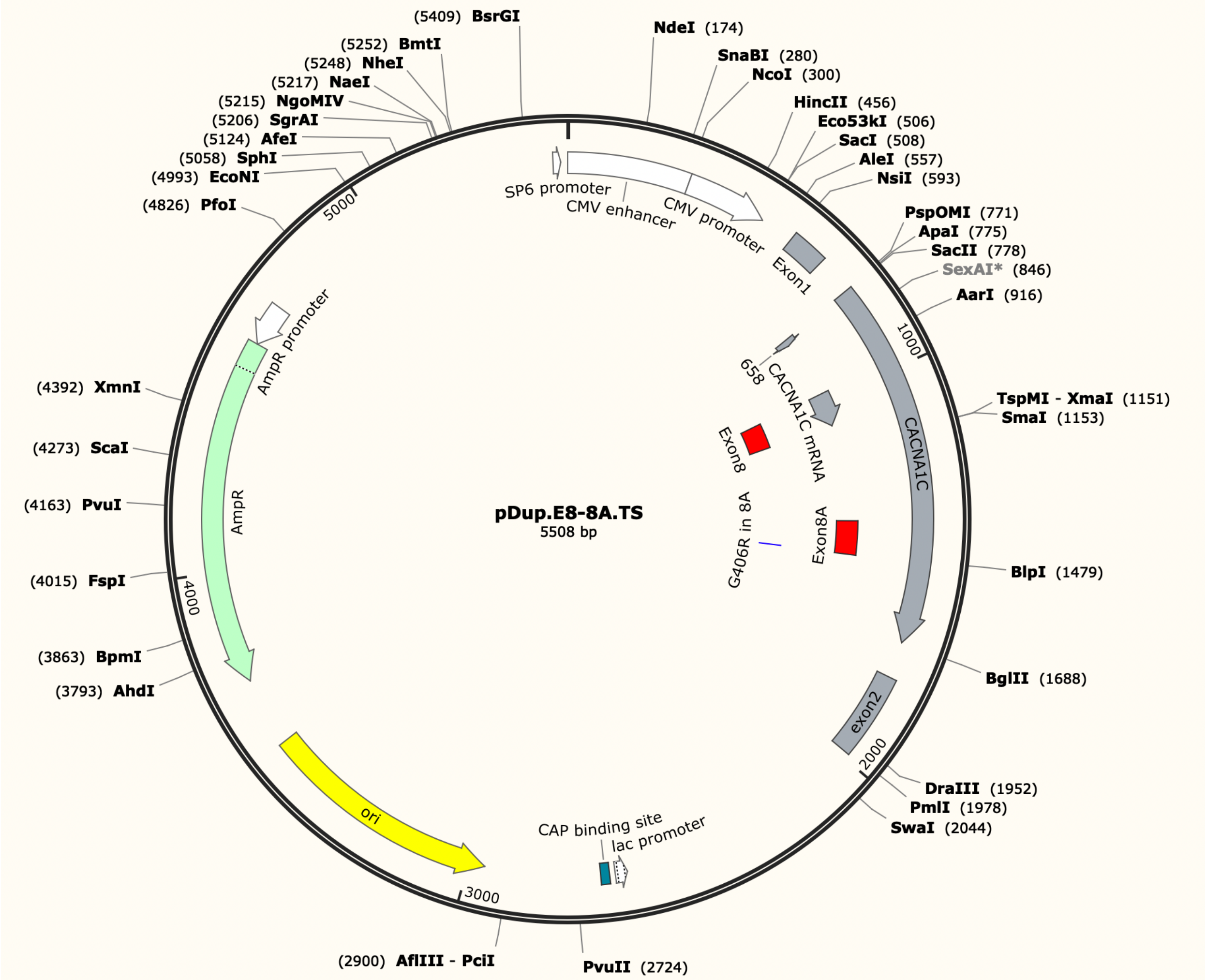

CGTTACATAACTTACGGTAAATGGCCCGCCTGGCTGACCGCCCAACGACCCCCGCCCATTGACGTCAATAATGACGTATGTTCCCATAGTAACGCCAATAGGGACTTT
CCATTGACGTCAATGGGTGGAGTATTTACGGTAAACTGCCCACTTGGCAGTACATCAAGTGTATCATATGCCAAGTCCGCCCCCTATTGACGTCAATGACGGTAAATG
GCCCGCCTGGCATTATGCCCAGTACATGACCTTACGGGACTTTCCTACTTGGCAGTACATCTACGTATTAGTCATCGCTATTACCATGGTGATGCGGTTTTGGCAGTAC
ACCAATGGGCGTGGATAGCGGTTTGACTCACGGGGATTTCCAAGTCTCCACCCCATTGACGTCAATGGGAGTTTGTTTTGGCACCAAAATCAACGGGACTTTCCAA
AATGTCGTAATAACCCCGCCCCGTTGACGCAAATGGGCGGTAGGCGTGTACGGTGGGAGGTCTATATAAGCAGAGCTCGTTTAGTGAACCGTCAGATCAGCTTGCT
TACATTTGCTTCTGACACAACTGTGTTCACTAGCAACCTCAAACAGACACCATGCATGGTGCACCTGACTCCTGAGGAGAAGTCTGCCGTTACTGCCCTGTGGGGCA
AGGTGAACGTGGATGAAGTTGGTGGTGAGGCCCTGGGCAGGTTGGTATCAAGGTTACAAGACAGGTTTAAGGAGACCAATAGAAACTGGGCATGTGGAGACAG
AGAAGACTCTTGGGTTTCTGATAGGGCCCGCGGCCGAGGTCCCCTTCGGTCACAGGAGTTCCTTTGAACATGGGCGATGCCCTGGGTAACACGGGTAACCTGGT
GCACATGAACAAAGCCCACGTTCAGCCCCGTCTGTCCCCTCCCAATCTGCTCACACCTGCTGCCTGCCTCTTTGCTGTAACCCAATTCTGCTTCTTCTTTCCTAACTTT
CCTTCGTCTTTCCAGATGCAGGACGCTATGGGCTATGAGTTACCCTGGGTGTATTTTGTCAGTCTGGTCATCTTTGGATCCTTTTTCGTTCTAAATCTGGTTCTCGGTG
TGTTGAGCGGGTAAGCTGACCGTTTCTATGTCCTCTCCACAACGCAGCCGAGCAAGGTCTCAGGTTCCACTCCGTACATGCCCGGGGTCCTCAGGGATGGGACCCT
GACAGGCCCAGGAAAACCACAACAAAGCCTCTGTTCAACCACAGATTCTGACCCATTGGCCAGGCAGGCTGTTTGGCCTCTGATTTGCACCTAGAGGGTCCCCGG
ATCCTGGCGCTGCGTGGGTCAGTGTCTCGGGAGCCGGGGACCGGCACTGGCCGTGCTCGGTTGCTGAGTGTGCCTCACTAACTATCATTCCGTTCTTCCAGGTCAA
TGATGCCGTAGGAAGGGACTGGCCCTGGATCTATTTTGTTACACTAATCATCATAGGGTCATTTTTTGTACTTAACTTGGTTCTCGGTGTGCTTAGCAGGTAAGCAGG
ACCAAGGAAAAAGGTCTTGATTTTTCCATTTATTTTTATTTATTCTTTCTGCTATTCCTGGCTGTATTCTTTTTCTGGCTCTGATGAACCTGGGATAAGGGGTCACCACA
GGAGCCTTGAAGTGGATGTCCTTGTCCTGGCTGGGTAAAGGGTCAGATGTGCCACTGGTCTTGGTGGTGGACAATGGAGGAGAGATCTGGGCATGTGGAGACAG
AGAAGACTCTTGGGTTTCTGATAGGCACTGACTCTCTCTGCCTATTGGTCTATTTTCCCACCCTTAGGCTGCTGGTGGTCTACCCTTGGACCCAGAGGTTCTTTGAGT
CCTTTGGGGATCTGTCCACTCCTGATGCTGTTATGGGCAACCCTAAGGTGAAGGCTCATGGCAAGAAAGTGCTCGGTGCCTTTAGTGATGGCCTGGCTCACCTGGA
CAACCTCAAGGGCACCTTTGCCACACTGAGTGAGCTGCACTGTGACAAGCTGCACGTGGATCCTTGAGCATCTGGATTCTGCCTAATAAAAAACATTTATTTTCATT
GCAATGATGTATTTAAATTATTTCTGAATATTTTACTAAAAAGGGAATGTGGGAGGTCAGTGCATTTAAAACATAAAGAAATGAAGAGCTAGTTCAAACCTTGGGAA
AATACACTATATCTTAAACTCCATGAAAGAAGGTGAGGCTGCAAACAGCTAATGCACATTGGCAACAGCCCTGATGCCTATGCCTTATTCATCCCTCAGAAAAGGATT
CAAGTAGAGGCTTGATTTGGAGGTTAAAGTTTTGCTATGCTGTATTTTACATTACTTATTGTTTTAGCTGTCCTCATGAATGTCTTTTCACTACCCATTTGCTTATCCTGC
ATCTCTCAGCCTTGACTCCACTCAGTTCTCTTGCTTAGAGATACCACCTTTCCCCTGAAGTGTTCCTTCCATGTTTTACGGCGAGATGGTTTCTCCTCGCCTGGCCACT
CAGCCTTAGTTGTCTCTGTTGTCTTATAGAGGTCTACTTGAAGAAGGAAAAACAGGGGGCATGGTTTGACTGTCCTGTGAGCCCTTCTTCCCTGCCTCCCCCACTCA

CAGTGACCCGGAATCATCCGCTCACAATTCCACACAACATACGAGCCGGAAGCATAAAGTGTAAAGCCTGGGGTGCCTAATGAGTGAGCTAACTCACATTAATTGCG
TTGCGCTCACTGCCCGCTTTCCAGTCGGGAAACCTGTCGTGCCAGCTGCATTAATGAATCGGCCAACGCGCGGGGAGAGGCGGTTTGCGTATTGGGCGCTCTTCC
GCTTCCTCGCTCACTGACTCGCTGCGCTCGGTCGTTCGGCTGCGGCGAGCGGTATCAGCTCACTCAAAGGCGGTAATACGGTTATCCACAGAATCAGGGGATAACG
CAGGAAAGAACATGTGAGCAAAAGGCCAGCAAAAGGCCAGGAACCGTAAAAAGGCCGCGTTGCTGGCGTTTTTCCATAGGCTCCGCCCCCCTGACGAGCATCAC
AAAAATCGACGCTCAAGTCAGAGGTGGCGAAACCCGACAGGACTATAAAGATACCAGGCGTTTCCCCCTGGAAGCTCCCTCGTGCGCTCTCCTGTTCCGACCCTGC
CGCTTACCGGATACCTGTCCGCCTTTCTCCCTTCGGGAAGCGTGGCGCTTTCTCATAGCTCACGCTGTAGGTATCTCAGTTCGGTGTAGGTCGTTCGCTCCAAGCTGG
GCTGTGTGCACGAACCCCCCGTTCAGCCCGACCGCTGCGCCTTATCCGGTAACTATCGTCTTGAGTCCAACCCGGTAAGACACGACTTATCGCCACTGGCAGCAGCC
ACTGGTAACAGGATTAGCAGAGCGAGGTATGTAGGCGGTGCTACAGAGTTCTTGAAGTGGTGGCCTAACTACGGCTACACTAGAAGAACAGTATTTGGTATCTGCG
CTCTGCTGAAGCCAGTTACCTTCGGAAAAAGAGTTGGTAGCTCTTGATCCGGCAAACAAACCACCGCTGGTAGCGGTGGTTTTTTTGTTTGCAAGCAGCAGATTAC
GCGCAGAAAAAAAGGATCTCAAGAAGATCCTTTGATCTTTTCTACGGGGTCTGACGCTCAGTGGAACGAAAACTCACGTTAAGGGATTTTGGTCATGAGATTATCA
AAAAGGATCTTCACCTAGATCCTTTTAAATTAAAAATGAAGTTTTAAATCAATCTAAAGTATATATGAGTAAACTTGGTCTGACAGTTACCAATGCTTAATCAGTGAGG
CACCTATCTCAGCGATCTGTCTATTTCGTTCATCCATAGTTGCCTGACTCCCCGTCGTGTAGATAACTACGATACGGGAGGGCTTACCATCTGGCCCCAGTGCTGCAAT
GATACCGCGAGACCCACGCTCACCGGCTCCAGATTTATCAGCAATAAACCAGCCAGCCGGAAGGGCCGAGCGCAGAAGTGGTCCTGCAACTTTATCCGCCTCCATC
CAGTCTATTAATTGTTGCCGGGAAGCTAGAGTAAGTAGTTCGCCAGTTAATAGTTTGCGCAACGTTGTTGCCATTGCTACAGGCATCGTGGTGTCACGCTCGTCGTTT
GGTATGGCTTCATTCAGCTCCGGTTCCCAACGATCAAGGCGAGTTACATGATCCCCCATGTTGTGCAAAAAAGCGGTTAGCTCCTTCGGTCCTCCGATCGTTGTCAG
AAGTAAGTTGGCCGCAGTGTTATCACTCATGGTTATGGCAGCACTGCATAATTCTCTTACTGTCATGCCATCCGTAAGATGCTTTTCTGTGACTGGTGAGTACTCAACC
AAGTCATTCTGAGAATAGTGTATGCGGCGACCGAGTTGCTCTTGCCCGGCGTCAATACGGGATAATACCGCGCCACATAGCAGAACTTTAAAAGTGCTCATCATTGG
AAAACGTTCTTCGGGGCGAAAACTCTCAAGGATCTTACCGCTGTTGAGATCCAGTTCGATGTAACCCACTCGTGCACCCAACTGATCTTCAGCATCTTTTACTTTCAC
CAGCGTTTCTGGGTGAGCAAAAACAGGAAGGCAAAATGCCGCAAAAAAGGGAATAAGGGCGACACGGAAATGTTGAATACTCATACTCTTCCTTTTTCAATATTAT
TGAAGCATTTATCAGGGTTATTGTCTCATGAGCGGATACATATTTGAATGTATTTAGAAAAATAAACAAATAGGGGTTCCGCGCACATTTCCCCGAAAAGTGCCACCT
GACGTCTAAGAAACCATTATTATCATGACATTAACCTATAAAAATAGGCGTATCACGAGGCCCTTTCGTCTCGCGCGTTTCGGTGATGACGGTGAAAACCTCTGACAC
ATGCAGCTCCCGGAGACGGTCACAGCTTGTCTGTAAGCGGATGCCGGGAGCAGACAAGCCCGTCAGGGCGCGTCAGCGGGTGTTGGCGGGTGTCGGGGCTGGC
TTAACTATGCGGCATCAGAGCAGATTGTACTGAGAGTGCACCATTCGACGCTCTCCCTTATGCGACTCCTGCATTAGGAAGCAGCCCAGTAGTAGGTTGAGGCCGTT
GAGCACCGCCGCCGCAAGGAATGGTGCATGCAAGGAGATGGCGCCCAACAGTCCCCCGGCCACGGGGCCTGCCACCATACCCACGCCGAAACAAGCGCTCATGA
GCCCGAAGTGGCGAGCCCGATCTTCCCCATCGGTGATGTCGGCGATATAGGCGCCAGCAACCGCACCTGTGGCGCCGGTGATGCCGGCCACGATGCGTCCGGCGT
AGAGGATCTGGCTAGCGATGACCCTGCTGATTGGTTCGCTGACCATTTCCGGGTGCGGGACGGCGTTACCAGAAACTCAGAAGGTTCGTCCAACCAAACCGACTC
TGACGGCAGTTTACGAGAGAGATGATAGGGTCTGCTTCAGTAAGCCAGATGCTACACAATTAGGCTTGTACATATTGTCGTTAGAACGCGGCTACAATTAATACATAA
CCTTATGTATCATACACATACGATTTAGGTGACACTATAGAATACAAGCTGATCCGTC

Supplementary Figure 5

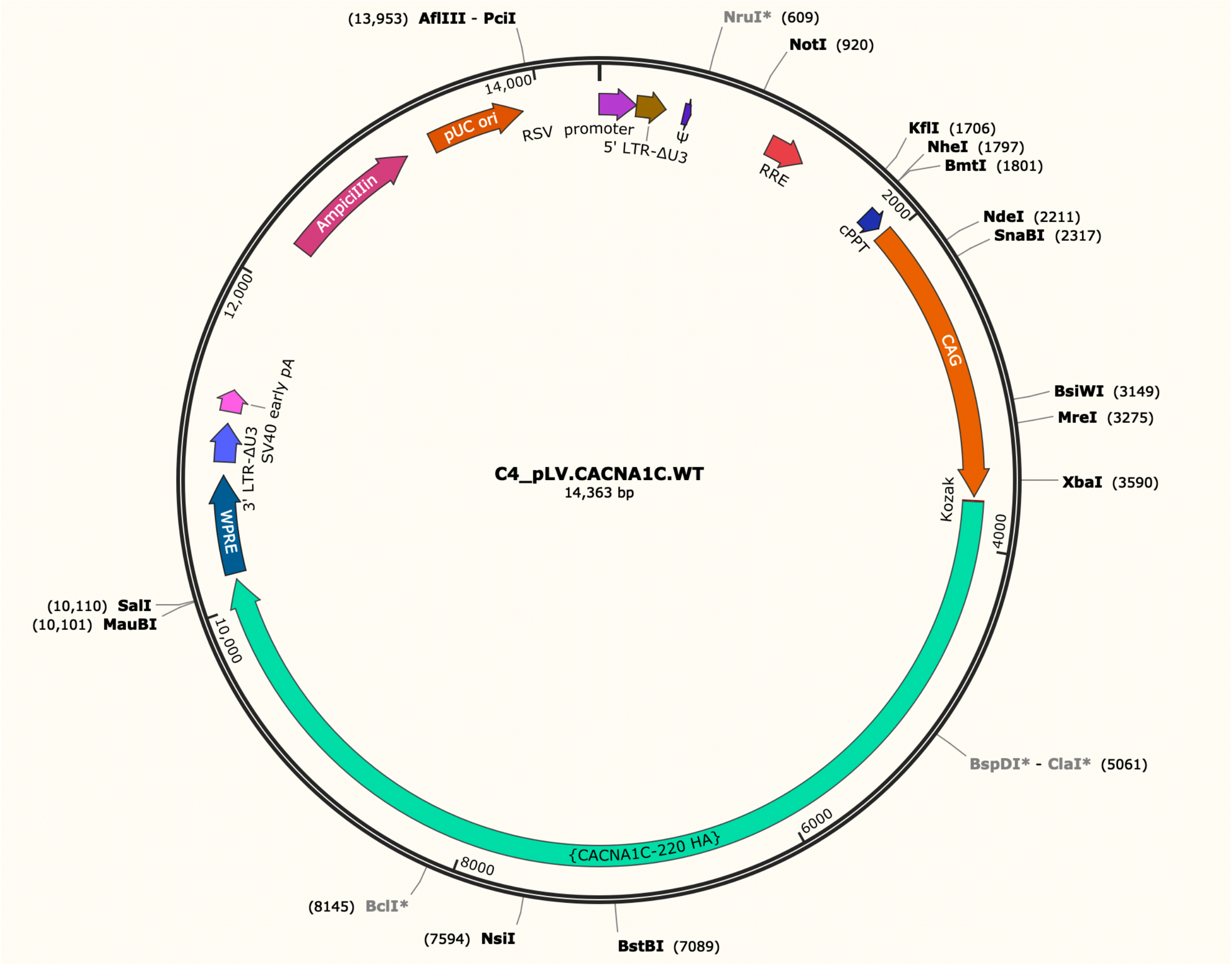

AATGTAGTCTTATGCAATACTCTTGTAGTCTTGCAACATGGTAACGATGAGTTAGCAACATGCCTTACAAGGAGAGAAAAAGCACCGTGCATGCCGATTGGTGGAAG
TAAGGTGGTACGATCGTGCCTTATTAGGAAGGCAACAGACGGGTCTGACATGGATTGGACGAACCACTGAATTGCCGCATTGCAGAGATATTGTATTTAAGTGCCTA
GCTCGATACATAAACGGGTCTCTCTGGTTAGACCAGATCTGAGCCTGGGAGCTCTCTGGCTAACTAGGGAACCCACTGCTTAAGCCTCAATAAAGCTTGCCTTGAGT
GCTTCAAGTAGTGTGTGCCCGTCTGTTGTGTGACTCTGGTAACTAGAGATCCCTCAGACCCTTTTAGTCAGTGTGGAAAATCTCTAGCAGTGGCGCCCGAACAGGG
ACTTGAAAGCGAAAGGGAAACCAGAGGAGCTCTCTCGACGCAGGACTCGGCTTGCTGAAGCGCGCACGGCAAGAGGCGAGGGGCGGCGACTGGTGAGTACGC
CAAAAATTTTGACTAGCGGAGGCTAGAAGGAGAGAGATGGGTGCGAGAGCGTCAGTATTAAGCGGGGGAGAATTAGATCGCGATGGGAAAAAATTCGGTTAAG
GCCAGGGGGAAAGAAAAAATATAAATTAAAACATATAGTATGGGCAAGCAGGGAGCTAGAACGATTCGCAGTTAATCCTGGCCTGTTAGAAACATCAGAAGGCTG
TAGACAAATACTGGGACAGCTACAACCATCCCTTCAGACAGGATCAGAAGAACTTAGATCATTATATAATACAGTAGCAACCCTCTATTGTGTGCATCAAAGGATAGA
GATAAAAGACACCAAGGAAGCTTTAGACAAGATAGAGGAAGAGCAAAACAAAAGTAAGACCACCGCACAGCAAGCGGCCGCTGATCTTCAGACCTGGAGGAGG
AGATATGAGGGACAATTGGAGAAGTGAATTATATAAATATAAAGTAGTAAAAATTGAACCATTAGGAGTAGCACCCACCAAGGCAAAGAGAAGAGTGGTGCAGAG
AGAAAAAAGAGCAGTGGGAATAGGAGCTTTGTTCCTTGGGTTCTTGGGAGCAGCAGGAAGCACTATGGGCGCAGCGTCAATGACGCTGACGGTACAGGCCAGA
CAATTATTGTCTGGTATAGTGCAGCAGCAGAACAATTTGCTGAGGGCTATTGAGGCGCAACAGCATCTGTTGCAACTCACAGTCTGGGGCATCAAGCAGCTCCAGG
CAAGAATCCTGGCTGTGGAAAGATACCTAAAGGATCAACAGCTCCTGGGGATTTGGGGTTGCTCTGGAAAACTCATTTGCACCACTGCTGTGCCTTGGAATGCTAG
TTGGAGTAATAAATCTCTGGAACAGATTTGGAATCACACGACCTGGATGGAGTGGGACAGAGAAATTAACAATTACACAAGCTTAATACACTCCTTAATTGAAGAAT
CGCAAAACCAGCAAGAAAAGAATGAACAAGAATTATTGGAATTAGATAAATGGGCAAGTTTGTGGAATTGGTTTAACATAACAAATTGGCTGTGGTATATAAAATTA
TTCATAATGATAGTAGGAGGCTTGGTAGGTTTAAGAATAGTTTTTGCTGTACTTTCTATAGTGAATAGAGTTAGGCAGGGATATTCACCATTATCGTTTCAGACCCACC
TCCCAACCCCGAGGGGACCCGACAGGCCCGAAGGAATAGAAGAAGAAGGTGGAGAGAGAGACAGAGACAGATCCATTCGATTAGTGAACGGATCTCGACGGTA
TCGCTAGCTTTTAAAAGAAAAGGGGGGATTGGGGGGTACAGTGCAGGGGAAAGAATAGTAGACATAATAGCAACAGACATACAAACTAAAGAATTACAAAAACA
AATTACAAAAATTCAAAATTTTACTAGTGATTATCGGATCAACTTTGTATAGAAAAGTTGCTCGACATTGATTATTGACTAGTTATTAATAGTAATCAATTACGGGGTCA
TTAGTTCATAGCCCATATATGGAGTTCCGCGTTACATAACTTACGGTAAATGGCCCGCCTGGCTGACCGCCCAACGACCCCCGCCCATTGACGTCAATAATGACGTATG
TTCCCATAGTAACGCCAATAGGGACTTTCCATTGACGTCAATGGGTGGAGTATTTACGGTAAACTGCCCACTTGGCAGTACATCAAGTGTATCATATGCCAAGTACGC
CCCCTATTGACGTCAATGACGGTAAATGGCCCGCCTGGCATTATGCCCAGTACATGACCTTATGGGACTTTCCTACTTGGCAGTACATCTACGTATTAGTCATCGCTATT
ACCATGGTCGAGGTGAGCCCCACGTTCTGCTTCACTCTCCCCATCTCCCCCCCCTCCCCACCCCCAATTTTGTATTTATTTATTTTTTAATTATTTTGTGCAGCGATGGG
GGCGGGGGGGGGGGGGGGGCGCGCCAGGCGGGGCGGGGCGGGGCGAGGGGCGGGGCGGGGCGAGGCGGAGAGGTGCGGCGGCAGCCAATCAGAGC
GGCGCGCTCCGAAAGTTTCCTTTTATGGCGAGGCGGCGGCGGCGGCGGCCCTATAAAAAGCGAAGCGCGCGGCGGGCGGGAGTCGCTGCGCGCTGCCTTCGCC
CCGTGCCCCGCTCCGCCGCCGCCTCGCGCCGCCCGCCCCGGCTCTGACTGACCGCGTTACTCCCACAGGTGAGCGGGCGGGACGGCCCTTCTCCTCCGGGCTGTA

ATTAGCGCTTGGTTTAATGACGGCTTGTTTCTTTTCTGTGGCTGCGTGAAAGCCTTGAGGGGCTCCGGGAGGGCCCTTTGTGCGGGGGGAGCGGCTCGGGGGGT
GCGTGCGTGTGTGTGTGCGTGGGGAGCGCCGCGTGCGGCTCCGCGCTGCCCGGCGGCTGTGAGCGCTGCGGGCGCGGCGCGGGGCTTTGTGCGCTCCGCAGTG
TGCGCGAGGGGAGCGCGGCCGGGGGCGGTGCCCCGCGGTGCGGGGGGGGCTGCGAGGGGAACAAAGGCTGCGTGCGGGGTGTGTGCGTGGGGGGGTGAGC
AGGGGGTGTGGGCGCGTCGGTCGGGCTGCAACCCCCCCTGCACCCCCCTCCCCGAGTTGCTGAGCACGGCCCGGCTTCGGGTGCGGGGCTCCGTACGGGGCGT
GGCGCGGGGCTCGCCGTGCCGGGCGGGGGGTGGCGGCAGGTGGGGGTGCCGGGCGGGGCGGGGCCGCCTCGGGCCGGGGAGGGCTCGGGGGAGGGGCGC
GGCGGCCCCCGGAGCGCCGGCGGCTGTCGAGGCGCGGCGAGCCGCAGCCATTGCCTTTTATGGTAATCGTGCGAGAGGGCGCAGGGACTTCCTTTGTCCCAAAT
CTGTGCGGAGCCGAAATCTGGGAGGCGCCGCCGCACCCCCTCTAGCGGGCGCGGGGCGAAGCGGTGCGGCGCCGGCAGGAAGGAAATGGGCGGGGAGGGCC
TTCGTGCGTCGCCGCGCCGCCGTCCCCTTCTCCCTCTCCAGCCTCGGGGCTGTCCGCGGGGGGACGGCTGCCTTCGGGGGGGACGGGGCAGGGCGGGGTTCGG
CTTCTGGCGTGTGACCGGCGGCTCTAGAGCCTCTGCTAACCATGTTCATGCCTTCTTCTTTTTCCTACAGCTCCTGGGCAACGTGCTGGTTATTGTGCTGTCTCATCAT
TTTGGCAAAGAATTGCAAGTTTGTACAAAAAAGCAGGCTGCCACCATGGTCAATGAGAATACGAGGATGTACATTCCAGAGGAAAACCACCAAGGTTCCAACTATG
GGAGCCCACGCCCCGCCCATGCCAACATGAATGCCAATGCGGCAGCGGGGCTGGCCCCTGAGCACATCCCCACCCCGGGGGCTGCCCTGTCGTGGCAGGCGGCC
ATCGACGCAGCCCGGCAGGCTAAGCTGATGGGCAGCGCTGGCAATGCGACCATCTCCACAGTCAGCTCCACGCAGCGGAAGCGGCAGCAATATGGGAAACCCAA
GAAGCAGGGCAGCACCACGGCCACACGCCCGCCCCGAGCCCTGCTCTGCCTGACCCTGAAGAACCCCATCCGGAGGGCCTGCATCAGCATTGTCGAATGGAAAC
CATTTGAAATAATTATTTACTGACTATTTTTGCCAATTGTGTGGCCTTAGCGATCTATATTCCCTTTCCAGAAGATGATTCCAACGCCACCAATTCCAACCTGGAACGA
GTGGAATATCTCTTTCTCATAATTTTTACGGTGGAAGCGTTTTTAAAAGTAATCGCCTATGGACTCCTCTTTCACCCCAATGCCTACCTCCGCAACGGCTGGAACCTAC
TAGATTTTATAATTGTGGTTGTGGGGCTTTTTAGTGCAATTTTAGAACAAGCAACCAAAGCAGATGGGGCAAACGCTCTCGGAGGGAAAGGGGCCGGATTTGATG
TGAAGGCGCTGAGGGCCTTCCGCGTGCTGCGCCCCCTGCGGCTGGTGTCCGGAGTCCCAAGTCTCCAGGTGGTCCTGAATTCCATCATCAAGGCCATGGTCCCCCT
GCTGCACATCGCCCTGCTTGTGCTGTTTGTCATCATCATCTACGCCATCATCGGCTTGGAGCTCTTCATGGGGAAGATGCACAAGACCTGCTACAACCAGGAGGGCA
TAGCAGATGTTCCAGCAGAAGATGACCCTTCCCCTTGTGCGCTGGAAACGGGCCACGGGCGGCAGTGCCAGAACGGCACGGTGTGCAAGCCCGGCTGGGATGGT
CCCAAGCACGGCATCACCAACTTTGACAACTTTGCCTTCGCCATGCTCACGGTGTTCCAGTGCATCACCATGGAGGGCTGGACGGACGTGCTGTACTGGGTCAATG
ATGCCGTAGGAAGGGACTGGCCCTGGATCTATTTTGTTACACTAATCATCATAGGGTCATTTTTTGTACTTAACTTGGTTCTCGGTGTGCTTAGCAGAGAGTTTTCCA
AAGAGAGGGAGAAGGCCAAGGCCCGGGGAGATTTCCAGAAGCTGCGGGAGAAGCAGCAGCTAGAAGAGGATCTCAAAGGCTACCTGGATTGGATCACTCAGG
CCGAAGACATCGATCCTGAGAATGAGGACGAAGGCATGGATGAGGAGAAGCCCCGAAACATGAGCATGCCCACCAGTGAGACCGAGTCCGTCAACACCGAAAAC
GTGGCTGGAGGTGACATCGAGGGAGAAAACTGCGGGGCCAGGCTGGCCCACCGGATCTCCAAGTCAAAGTTCAGCCGCTACTGGCGCCGGTGGAATCGGTTCTG
CAGAAGGAAGTGCCGCGCCGCAGTCAAGTCTAATGTCTTCTACTGGCTGGTGATTTTCCTGGTGTTCCTCAACACGCTCACCATTGCCTCTGAGCACTACAACCAGC
CCAACTGGCTCACAGAAGTCCAAGACACGGCAAACAAGGCCCTGCTGGCCCTGTTCACGGCAGAGATGCTCCTGAAGATGTACAGCCTGGGCCTGCAGGCCTACT
TCGTGTCCCTCTTCAACCGCTTTGACTGCTTCGTCGTGTGTGGCGGCATCCTGGAGACCATCCTGGTGGAGACCAAGATCATGTCCCCACTGGGCATCTCCGTGCTC
AGATGCGTCCGGCTGCTGAGGATTTTCAAGATCACGAGGTACTGGAACTCCTTGAGCAACCTGGTGGCATCCTTGCTGAACTCTGTGCGCTCCATCGCCTCCCTGCT
CCTTCTCCTCTTCCTCTTCATCATCATCTTCTCCCTCCTGGGGATGCAGCTCTTTGGAGGAAAGTTCAACTTTGATGAGATGCAGTACCCATACGATGTTCCAGATTAC
GCTACCCGGAGGAGCACATTCGATAACTTCCCCCAGTCCCTCCTCACTGTGTTTCAGATCCTGACCGGGGAGGACTGGAATTCGGTGATGTATGATGGGATCATGGC
TTATGGCGGCCCCTCTTTTCCAGGGATGTTAGTCTGTATTTACTTCATCATCCTCTTCATCTGTGGAAACTATATCCTACTGAATGTGTTCTTGGCCATTGCTGTGGACA
ACCTGGCTGATGCTGAGAGCCTCACATCTGCCCAAAAGGAGGAGGAAGAGGAGAAGGAGAGAAAGAAGCTGGCCAGGACTGCCAGCCCAGAGAAGAAACAAG
AGTTGGTGGAGAAGCCGGCAGTGGGGGAATCCAAGGAGGAGAAGATTGAGCTGAAATCCATCACGGCTGACGGAGAGTCTCCACCCGCCACCAAGATCAACAT
GGATGACCTCCAGCCCAATGAAAATGAGGATAAGAGCCCCTACCCCAACCCAGAAACTACAGGAGAAGAGGATGAGGAGGAGCCAGAGATGCCTGTCGGCCCTC
GCCCACGACCACTCTCTGAGCTTCACCTTAAGGAAAAGGCAGTGCCCATGCCAGAAGCCAGCGCGTTTTTCATCTTCAGCTCTAACAACAGGTTTCGCCTCCAGTG
CCACCGCATTGTCAATGACACGATCTTCACCAACCTGATCCTCTTCTTCATTCTGCTCAGCAGCATTTCCCTGGCTGCTGAGGACCCGGTCCAGCACACCTCCTTCAG
GAACCATATTCTGTTTTATTTTGATATTGTTTTTACCACCATTTTCACCATTGAAATTGCTCTGAAGATGACTGCTTATGGGGCTTTCTTGCACAAGGGTTCTTTCTGCC
GGAACTACTTCAACATCCTGGACCTGCTGGTGGTCAGCGTGTCCCTCATCTCCTTTGGCATCCAGTCCAGTGCAATCAATGTCGTGAAGATCTTGCGAGTCCTGCGA
GTACTCAGGCCCCTGAGGGCCATCAACAGGGCCAAGGGGCTAAAGCATGTGGTTCAGTGTGTGTTTGTCGCCATCCGGACCATCGGGAACATCGTGATTGTCACCA
CCCTGCTGCAGTTCATGTTTGCCTGCATCGGGGTCCAGCTCTTCAAGGGAAAGCTGTACACCTGTTCAGACAGTTCCAAGCAGACAGAGGCGGAATGCAAGGGCA
ACTACATCACGTACAAAGACGGGGAGGTTGACCACCCCATCATCCAACCCCGCAGCTGGGAGAACAGCAAGTTTGACTTTGACAATGTTCTGGCAGCCATGATGGC
CCTCTTCACCGTCTCCACCTTCGAAGGGTGGCCAGAGCTGCTGTACCGCTCCATCGACTCCCACACGGAAGACAAGGGCCCCATCTACAACTACCGTGTGGAGATC
TCCATCTTCTTCATCATCTACATCATCATCATCGCCTTCTTCATGATGAACATCTTCGTGGGCTTCGTCATCGTCACCTTTCAGGAGCAGGGGGAGCAGGAGTACAAGA
ACTGTGAGCTGGACAAGAACCAGCGACAGTGCGTGGAATACGCCCTCAAGGCCCGGCCCCTGCGGAGGTACATCCCCAAGAACCAGCACCAGTACAAAGTGTGG
TACGTGGTCAACTCCACCTACTTCGAGTACCTGATGTTCGTCCTCATCCTGCTCAACACCATCTGCCTGGCCATGCAGCACTACGGCCAGAGCTGCCTGTTCAAAATC
GCCATGAACATCCTCAACATGCTCTTCACTGGCCTCTTCACCGTGGAGATGATCCTGAAGCTCATTGCCTTCAAACCCAAGCACTATTTCTGTGATGCATGGAATACAT
TTGACGCCTTGATTGTTGTGGGTAGCATTGTTGATATAGCAATCACCGAGGTAAACCCAGCTGAACATACCCAATGCTCTCCCTCTATGAACGCAGAGGAAAACTCCC
GCATCTCCATCACCTTCTTCCGCCTGTTCCGGGTCATGCGTCTGGTGAAGCTGCTGAGCCGTGGGGAGGGCATCCGGACGCTGCTGTGGACCTTCATCAAGTCCTTC
CAGGCCCTGCCCTATGTGGCCCTCCTGATCGTGATGCTGTTCTTCATCTACGCGGTGATCGGGATGCAGGTGTTTGGGAAAATTGCCCTGAATGATACCACAGAGAT
CAACCGGAACAACAACTTTCAGACCTTCCCCCAGGCCGTGCTGCTCCTCTTCAGGTGTGCCACCGGGGAGGCCTGGCAGGACATCATGCTGGCCTGCATGCCAGG
CAAGAAGTGTGCCCCAGAGTCCGAGCCCAGCAACAGCACGGAGGGTGAAACACCCTGTGGTAGCAGCTTTGCTGTCTTCTACTTCATCAGCTTCTACATGCTCTGT
GCCTTCCTGATCATCAACCTCTTTGTAGCTGTCATCATGGACAACTTTGACTACCTGACAAGGGACTGGTCCATCCTTGGTCCCCACCACCTGGATGAGTTTAAAAGA
ATCTGGGCAGAGTATGACCCTGAAGCCAAGGGTCGTATCAAACACCTGGATGTGGTGACCCTCCTCCGGCGGATTCAGCCGCCACTAGGTTTTGGGAAGCTGTGCC
CTCACCGCGTGGCTTGCAAACGCCTGGTCTCCATGAACATGCCTCTGAACAGCGACGGGACAGTCATGTTCAATGCCACCCTGTTTGCCCTGGTCAGGACGGCCCT
GAGGATCAAAACAGAAGGGAACCTAGAACAAGCCAATGAGGAGCTGCGGGCGATCATCAAGAAGATCTGGAAGCGGACCAGCATGAAGCTGCTGGACCAGGTG
GTGCCCCCTGCAGGTGATGATGAGGTCACCGTTGGCAAGTTCTACGCCACGTTCCTGATCCAGGAGTACTTCCGGAAGTTCAAGAAGCGCAAAGAGCAGGGCCTT
GTGGGCAAGCCCTCCCAGAGGAACGCGCTGTCTCTGCAGGCTGGCTTGCGCACACTGCATGACATCGGGCCTGAGATCCGACGGGCCATCTCTGGAGATCTCACC
GCTGAGGAGGAGCTGGACAAGGCCATGAAGGAGGCTGTGTCCGCTGCTTCTGAAGATGACATCTTCAGGAGGGCCGGTGGCCTGTTCGGCAACCACGTCAGCTA
CTACCAAAGCGACGGCCGGAGCGCCTTCCCCCAGACCTTCACCACTCAGCGCCCGCTGCACATCAACAAGGCGGGCAGCAGCCAGGGCGACACTGAGTCGCCAT
CCCACGAGAAGCTGGTGGACTCCACCTTCACCCCGAGCAGCTACTCGTCCACCGGCTCCAACGCCAACATCAACAACGCCAACAACACCGCCCTGGGTCGCCTCCC
TCGCCCCGCCGGCTACCCCAGCACGGTCAGCACTGTGGAGGGCCACGGGCCCCCCTTGTCCCCTGCCATCCGGGTGCAGGAGGTGGCGTGGAAGCTCAGCTCCA
ACAGGTGCCACTCCCGGGAGAGCCAGGCAGCCATGGCGGGTCAGGAGGAGACGTCTCAGGATGAGACCTATGAAGTGAAGATGAACCATGACACGGAGGCCTG
CAGTGAGCCCAGCCTGCTCTCCACAGAGATGCTCTCCTACCAGGATGACGAAAATCGGCAACTGACGCTCCCAGAGGAGGACAAGAGGGACATCCGGCAATCTCC
GAAGAGGGGTTTCCTCCGCTCTGCCTCACTAGGTCGAAGGGCCTCCTTCCACCTGGAATGTCTGAAGCGACAGAAGGACCGAGGGGGAGACATCTCTCAGAAGA
CAGTCCTGCCCTTGCATCTGGTTCATCATCAGGCATTGGCAGTGGCAGGCCTGAGCCCCCTCCTCCAGAGAAGCCATTCCCCTGCCTCATTCCCTAGGCCTTTTGCCA
CCCCACCAGCCACACCTGGCAGCCGAGGCTGGCCCCCACAGCCCGTCCCCACCCTGCGGCTTGAGGGGGTCGAGTCCAGTGAGAAACTCAACAGCAGCTTCCCA

TCCATCCACTGCGGCTCCTGGGCTGAGACCACCCCCGGTGGCGGGGGCAGCAGCGCCGCCCGGAGAGTCCGGCCCGTCTCCCTCATGGTGCCCAGCCAGGCTGG
GGCCCCAGGGAGGCAGTTCCACGGCAGTGCCAGCAGCCTGGTGGAAGCGGTCTTGATTTCAGAAGGACTGGGGCAGTTTGCTCAAGATCCCAAGTTCATCGAGG
TCACCACCCAGGAGCTGGCCGACGCCTGCGACATGACCATAGAGGAGATGGAGAGCGCGGCCGACAACATCCTCAGCGGGGGCGCCCCACAGAGCCCCAATGG
CGCCCTCTTACCCTTTGTGAACTGCAGGGACGCGGGGCAGGACCGAGCCGGGGGCGAAGAGGACGCGGGCTGTGTGCGCGCGCGGGGTCGACCGAGTGAGGA
GGAGCTCCAGGACAGCAGGGTCTACGTCAGCAGCCTGTAGACCCAGCTTTCTTGTACAAAGTGGTGATAATCGAATTCCGATAATCAACCTCTGGATTACAAAATTT
GTGAAAGATTGACTGGTATTCTTAACTATGTTGCTCCTTTTACGCTATGTGGATACGCTGCTTTAATGCCTTTGTATCATGCTATTGCTTCCCGTATGGCTTTCATTTTCT
CCTCCTTGTATAAATCCTGGTTGCTGTCTCTTTATGAGGAGTTGTGGCCCGTTGTCAGGCAACGTGGCGTGGTGTGCACTGTGTTTGCTGACGCAACCCCCACTGGT
TGGGGCATTGCCACCACCTGTCAGCTCCTTTCCGGGACTTTCGCTTTCCCCCTCCCTATTGCCACGGCGGAACTCATCGCCGCCTGCCTTGCCCGCTGCTGGACAGG
GGCTCGGCTGTTGGGCACTGACAATTCCGTGGTGTTGTCGGGGAAGCTGACGTCCTTTCCATGGCTGCTCGCCTGTGTTGCCACCTGGATTCTGCGCGGGACGTCC
TTCTGCTACGTCCCTTCGGCCCTCAATCCAGCGGACCTTCCTTCCCGCGGCCTGCTGCCGGCTCTGCGGCCTCTTCCGCGTCTTCGCCTTCGCCCTCAGACGAGTCG
GATCTCCCTTTGGGCCGCCTCCCCGCATCGGGAATTCCCGCGGTTCGCTTTAAGACCAATGACTTACAAGGCAGCTGTAGATCTTAGCCACTTTTTAAAAGAAAAGG
GGGGACTGGAAGGGCTAATTCACTCCCAACGAAGACAAGATCTGCTTTTTGCTTGTACTGGGTCTCTCTGGTTAGACCAGATCTGAGCCTGGGAGCTCTCTGGCTA
ACTAGGGAACCCACTGCTTAAGCCTCAATAAAGCTTGCCTTGAGTGCTTCAAGTAGTGTGTGCCCGTCTGTTGTGTGACTCTGGTAACTAGAGATCCCTCAGACCCT
TTTAGTCAGTGTGGAAAATCTCTAGCAGTAGTAGTTCATGTCATCTTATTATTCAGTATTTATAACTTGCAAAGAAATGAATATCAGAGAGTGAGAGGAACTTGTTTAT
TGCAGCTTATAATGGTTACAAATAAAGCAATAGCATCACAAATTTCACAAATAAAGCATTTTTTTCACTGCATTCTAGTTGTGGTTTGTCCAAACTCATCAATGTATCTT
ATCATGTCTGGCTCTAGCTATCCCGCCCCTAACTCCGCCCATCCCGCCCCTAACTCCGCCCAGTTCCGCCCATTCTCCGCCCCATGGCTGACTAATTTTTTTTATTTATGC
AGAGGCCGAGGCCGCCTCGGCCTCTGAGCTATTCCAGAAGTAGTGAGGAGGCTTTTTTGGAGGCCTAGGGACGTACCCAATTCGCCCTATAGTGAGTCGTATTACG
CGCGCTCACTGGCCGTCGTTTTACAACGTCGTGACTGGGAAAACCCTGGCGTTACCCAACTTAATCGCCTTGCAGCACATCCCCCTTTCGCCAGCTGGCGTAATAGC
GAAGAGGCCCGCACCGATCGCCCTTCCCAACAGTTGCGCAGCCTGAATGGCGAATGGGACGCGCCCTGTAGCGGCGCATTAAGCGCGGCGGGTGTGGTGGTTAC
GCGCAGCGTGACCGCTACACTTGCCAGCGCCCTAGCGCCCGCTCCTTTCGCTTTCTTCCCTTCCTTTCTCGCCACGTTCGCCGGCTTTCCCCGTCAAGCTCTAAATCG
GGGGCTCCCTTTAGGGTTCCGATTTAGTGCTTTACGGCACCTCGACCCCAAAAAACTTGATTAGGGTGATGGTTCACGTAGTGGGCCATCGCCCTGATAGACGGTTT
TTCGCCCTTTGACGTTGGAGTCCACGTTCTTTAATAGTGGACTCTTGTTCCAAACTGGAACAACACTCAACCCTATCTCGGTCTATTCTTTTGATTTATAAGGGATTTT
GCCGATTTCGGCCTATTGGTTAAAAAATGAGCTGATTTAACAAAAATTTAACGCGAATTTTAACAAAATATTAACGCTTACAATTTAGGTGGCACTTTTCGGGGAAAT
GTGCGCGGAACCCCTATTTGTTTATTTTTCTAAATACATTCAAATATGTATCCGCTCATGAGACAATAACCCTGATAAATGCTTCAATAATATTGAAAAAGGAAGAGTAT
GAGTATTCAACATTTCCGTGTCGCCCTTATTCCCTTTTTTGCGGCATTTTGCCTTCCTGTTTTTGCTCACCCAGAAACGCTGGTGAAAGTAAAAGATGCTGAAGATCA
GTTGGGTGCACGAGTGGGTTACATCGAACTGGATCTCAACAGCGGTAAGATCCTTGAGAGTTTTCGCCCCGAAGAACGTTTTCCAATGATGAGCACTTTTAAAGTT
CTGCTATGTGGCGCGGTATTATCCCGTATTGACGCCGGGCAAGAGCAACTCGGTCGCCGCATACACTATTCTCAGAATGACTTGGTTGAGTACTCACCAGTCACAGA
AAAGCATCTTACGGATGGCATGACAGTAAGAGAATTATGCAGTGCTGCCATAACCATGAGTGATAACACTGCGGCCAACTTACTTCTGACAACGATCGGAGGACCG
AAGGAGCTAACCGCTTTTTTGCACAACATGGGGGATCATGTAACTCGCCTTGATCGTTGGGAACCGGAGCTGAATGAAGCCATACCAAACGACGAGCGTGACACC
ACGATGCCTGTAGCAATGGCAACAACGTTGCGCAAACTATTAACTGGCGAACTACTTACTCTAGCTTCCCGGCAACAATTAATAGACTGGATGGAGGCGGATAAAGT
TGCAGGACCACTTCTGCGCTCGGCCCTTCCGGCTGGCTGGTTTATTGCTGATAAATCTGGAGCCGGTGAGCGTGGGTCTCGCGGTATCATTGCAGCACTGGGGCCA
GATGGTAAGCCCTCCCGTATCGTAGTTATCTACACGACGGGGAGTCAGGCAACTATGGATGAACGAAATAGACAGATCGCTGAGATAGGTGCCTCACTGATTAAGCA
TTGGTAACTGTCAGACCAAGTTTACTCATATATACTTTAGATTGATTTAAAACTTCATTTTTAATTTAAAAGGATCTAGGTGAAGATCCTTTTTGATAATCTCATGACCAA
AATCCCTTAACGTGAGTTTTCGTTCCACTGAGCGTCAGACCCCGTAGAAAAGATCAAAGGATCTTCTTGAGATCCTTTTTTTCTGCGCGTAATCTGCTGCTTGCAAAC
AAAAAAACCACCGCTACCAGCGGTGGTTTGTTTGCCGGATCAAGAGCTACCAACTCTTTTTCCGAAGGTAACTGGCTTCAGCAGAGCGCAGATACCAAATACTGTT
CTTCTAGTGTAGCCGTAGTTAGGCCACCACTTCAAGAACTCTGTAGCACCGCCTACATACCTCGCTCTGCTAATCCTGTTACCAGTGGCTGCTGCCAGTGGCGATAAG
TCGTGTCTTACCGGGTTGGACTCAAGACGATAGTTACCGGATAAGGCGCAGCGGTCGGGCTGAACGGGGGGTTCGTGCACACAGCCCAGCTTGGAGCGAACGAC
CTACACCGAACTGAGATACCTACAGCGTGAGCTATGAGAAAGCGCCACGCTTCCCGAAGAGAGAAAGGCGGACAGGTATCCGGTAAGCGGCAGGGTCGGAACAG
GAGAGCGCACGAGGGAGCTTCCAGGGGGAAACGCCTGGTATCTTTATAGTCCTGTCGGGTTTCGCCACCTCTGACTTGAGCGTCGATTTTTGTGATGCTCGTCAGG
GGGGCGGAGCCTATGGAAAAACGCCAGCAACGCGGCCTTTTTACGGTTCCTGGCCTTTTGCTGGCCTTTTGCTCACATGTTCTTTCCTGCGTTATCCCCTGATTCTG
TGGATAACCGTATTACCGCCTTTGAGTGAGCTGATACCGCTCGCCGCAGCCGAACGACCGAGCGCAGCGAGTCAGTGAGCGAGGAAGCGGAAGAGCGCCCAATA
CGCAAACCGCCTCTCCCCGCGCGTTGGCCGATTCATTAATGCAGCTGGCACGACAGGTTTCCCGACTGGAAAGCGGGCAGTGAGCGCAACGCAATTAATGTGAGT
TAGCTCACTCATTAGGCACCCCAGGCTTTACACTTTATGCTTCCGGCTCGTATGTTGTGTGGAATTGTGAGCGGATAACAATTTCACACAGGAAACAGCTATGACCAT
GATTACGCCAAGCGCGCAATTAACCCTCACTAAAGGGAACAAAAGCTGGAGCTGCAAGCTT

Supplementary Figure 6

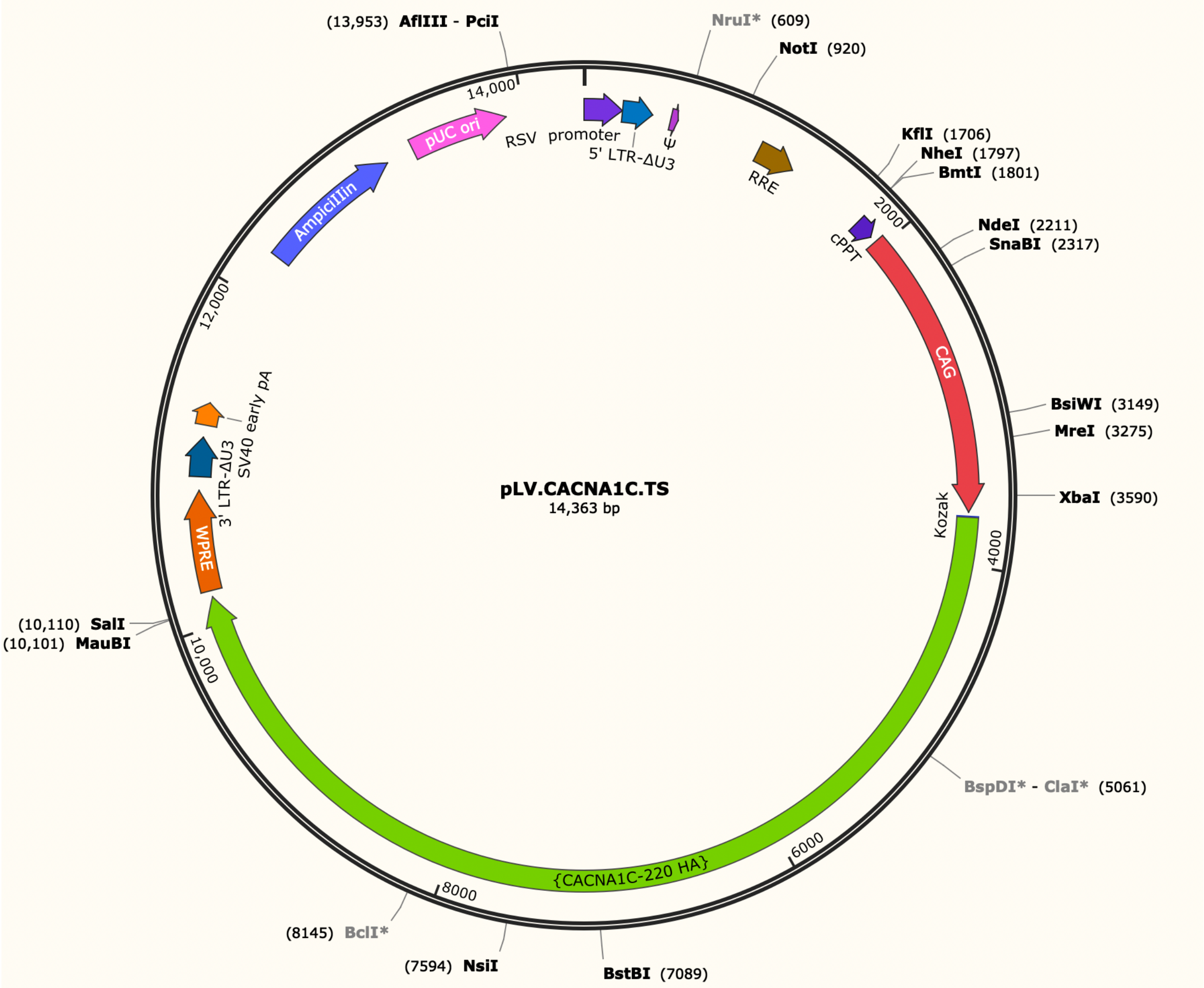

AATGTAGTCTTATGCAATACTCTTGTAGTCTTGCAACATGGTAACGATGAGTTAGCAACATGCCTTACAAGGAGAGAAAAAGCACCGTGCATGCCGATTGGTGGAAG
TAAGGTGGTACGATCGTGCCTTATTAGGAAGGCAACAGACGGGTCTGACATGGATTGGACGAACCACTGAATTGCCGCATTGCAGAGATATTGTATTTAAGTGCCTA
GCTCGATACATAAACGGGTCTCTCTGGTTAGACCAGATCTGAGCCTGGGAGCTCTCTGGCTAACTAGGGAACCCACTGCTTAAGCCTCAATAAAGCTTGCCTTGAGT
GCTTCAAGTAGTGTGTGCCCGTCTGTTGTGTGACTCTGGTAACTAGAGATCCCTCAGACCCTTTTAGTCAGTGTGGAAAATCTCTAGCAGTGGCGCCCGAACAGGG
ACTTGAAAGCGAAAGGGAAACCAGAGGAGCTCTCTCGACGCAGGACTCGGCTTGCTGAAGCGCGCACGGCAAGAGGCGAGGGGCGGCGACTGGTGAGTACGC
CAAAAATTTTGACTAGCGGAGGCTAGAAGGAGAGAGATGGGTGCGAGAGCGTCAGTATTAAGCGGGGGAGAATTAGATCGCGATGGGAAAAAATTCGGTTAAG
GCCAGGGGGAAAGAAAAAATATAAATTAAAACATATAGTATGGGCAAGCAGGGAGCTAGAACGATTCGCAGTTAATCCTGGCCTGTTAGAAACATCAGAAGGCTG
TAGACAAATACTGGGACAGCTACAACCATCCCTTCAGACAGGATCAGAAGAACTTAGATCATTATATAATACAGTAGCAACCCTCTATTGTGTGCATCAAAGGATAGA
GATAAAAGACACCAAGGAAGCTTTAGACAAGATAGAGGAAGAGCAAAACAAAAGTAAGACCACCGCACAGCAAGCGGCCGCTGATCTTCAGACCTGGAGGAGG
AGATATGAGGGACAATTGGAGAAGTGAATTATATAAATATAAAGTAGTAAAAATTGAACCATTAGGAGTAGCACCCACCAAGGCAAAGAGAAGAGTGGTGCAGAG
AGAAAAAAGAGCAGTGGGAATAGGAGCTTTGTTCCTTGGGTTCTTGGGAGCAGCAGGAAGCACTATGGGCGCAGCGTCAATGACGCTGACGGTACAGGCCAGA
CAATTATTGTCTGGTATAGTGCAGCAGCAGAACAATTTGCTGAGGGCTATTGAGGCGCAACAGCATCTGTTGCAACTCACAGTCTGGGGCATCAAGCAGCTCCAGG
CAAGAATCCTGGCTGTGGAAAGATACCTAAAGGATCAACAGCTCCTGGGGATTTGGGGTTGCTCTGGAAAACTCATTTGCACCACTGCTGTGCCTTGGAATGCTAG
TTGGAGTAATAAATCTCTGGAACAGATTTGGAATCACACGACCTGGATGGAGTGGGACAGAGAAATTAACAATTACACAAGCTTAATACACTCCTTAATTGAAGAAT
CGCAAAACCAGCAAGAAAAGAATGAACAAGAATTATTGGAATTAGATAAATGGGCAAGTTTGTGGAATTGGTTTAACATAACAAATTGGCTGTGGTATATAAAATTA
TTCATAATGATAGTAGGAGGCTTGGTAGGTTTAAGAATAGTTTTTGCTGTACTTTCTATAGTGAATAGAGTTAGGCAGGGATATTCACCATTATCGTTTCAGACCCACC
TCCCAACCCCGAGGGGACCCGACAGGCCCGAAGGAATAGAAGAAGAAGGTGGAGAGAGAGACAGAGACAGATCCATTCGATTAGTGAACGGATCTCGACGGTA
TCGCTAGCTTTTAAAAGAAAAGGGGGGATTGGGGGGTACAGTGCAGGGGAAAGAATAGTAGACATAATAGCAACAGACATACAAACTAAAGAATTACAAAAACA
AATTACAAAAATTCAAAATTTTACTAGTGATTATCGGATCAACTTTGTATAGAAAAGTTGCTCGACATTGATTATTGACTAGTTATTAATAGTAATCAATTACGGGGTCA
TTAGTTCATAGCCCATATATGGAGTTCCGCGTTACATAACTTACGGTAAATGGCCCGCCTGGCTGACCGCCCAACGACCCCCGCCCATTGACGTCAATAATGACGTATG
TTCCCATAGTAACGCCAATAGGGACTTTCCATTGACGTCAATGGGTGGAGTATTTACGGTAAACTGCCCACTTGGCAGTACATCAAGTGTATCATATGCCAAGTACGC
CCCCTATTGACGTCAATGACGGTAAATGGCCCGCCTGGCATTATGCCCAGTACATGACCTTATGGGACTTTCCTACTTGGCAGTACATCTACGTATTAGTCATCGCTATT
ACCATGGTCGAGGTGAGCCCCACGTTCTGCTTCACTCTCCCCATCTCCCCCCCCTCCCCACCCCCAATTTTGTATTTATTTATTTTTTAATTATTTTGTGCAGCGATGGG
GGCGGGGGGGGGGGGGGGGCGCGCGCCAGGCGGGGCGGGGCGGGGCGAGGGGCGGGGCGGGGCGAGGCGGAGAGGTGCGGCGGCAGCCAATCAGAGC
GGCGCGCTCCGAAAGTTTCCTTTTATGGCGAGGCGGCGGCGGCGGCGGCCCTATAAAAAGCGAAGCGCGCGGCGGGCGGGAGTCGCTGCGCGCTGCCTTCGCC

CCGTGCCCCGCTCCGCCGCCGCCTCGCGCCGCCCGCCCCGGCTCTGACTGACCGCGTTACTCCCACAGGTGAGCGGGCGGGACGGCCCTTCTCCTCCGGGCTGTA
ATTAGCGCTTGGTTTAATGACGGCTTGTTTCTTTTCTGTGGCTGCGTGAAAGCCTTGAGGGGCTCCGGGAGGGCCCTTTGTGCGGGGGGAGCGGCTCGGGGGGT
GCGTGCGTGTGTGTGTGCGTGGGGAGCGCCGCGTGCGGCTCCGCGCTGCCCGGCGGCTGTGAGCGCTGCGGGCGCGGCGCGGGGCTTTGTGCGCTCCGCAGTG
TGCGCGAGGGGAGCGCGGCCGGGGGCGGTGCCCCGCGGTGCGGGGGGGGCTGCGAGGGGAACAAAGGCTGCGTGCGGGGTGTGTGCGTGGGGGGGTGAGC
AGGGGGTGTGGGCGCGTCGGTCGGGCTGCAACCCCCCCTGCACCCCCCTCCCCGAGTTGCTGAGCACGGCCCGGCTTCGGGTGCGGGGCTCCGTACGGGGCGT
GGCGCGGGGCTCGCCGTGCCGGGCGGGGGGTGGCGGCAGGTGGGGGTGCCGGGCGGGGCGGGGCCGCCTCGGGCCGGGGAGGGCTCGGGGGAGGGGCGC
GGCGGCCCCCGGAGCGCCGGCGGCTGTCGAGGCGCGGCGAGCCGCAGCCATTGCCTTTTATGGTAATCGTGCGAGAGGGCGCAGGGACTTCCTTTGTCCCAAAT
CTGTGCGGAGCCGAAATCTGGGAGGCGCCGCCGCACCCCCTCTAGCGGGCGCGGGGCGAAGCGGTGCGGCGCCGGCAGGAAGGAAATGGGCGGGGAGGGCC
TTCGTGCGTCGCCGCGCCGCCGTCCCCTTCTCCCTCTCCAGCCTCGGGGCTGTCCGCGGGGGGACGGCTGCCTTCGGGGGGGACGGGGCAGGGCGGGGTTCGG
CTTCTGGCGTGTGACCGGCGGCTCTAGAGCCTCTGCTAACCATGTTCATGCCTTCTTCTTTTTCCTACAGCTCCTGGGCAACGTGCTGGTTATTGTGCTGTCTCATCAT
TTTGGCAAAGAATTGCAAGTTTGTACAAAAAAGCAGGCTGCCACCATGGTCAATGAGAATACGAGGATGTACATTCCAGAGGAAAACCACCAAGGTTCCAACTATG
GGAGCCCACGCCCCGCCCATGCCAACATGAATGCCAATGCGGCAGCGGGGCTGGCCCCTGAGCACATCCCCACCCCGGGGGCTGCCCTGTCGTGGCAGGCGGCC
ATCGACGCAGCCCGGCAGGCTAAGCTGATGGGCAGCGCTGGCAATGCGACCATCTCCACAGTCAGCTCCACGCAGCGGAAGCGGCAGCAATATGGGAAACCCAA
GAAGCAGGGCAGCACCACGGCCACACGCCCGCCCCGAGCCCTGCTCTGCCTGACCCTGAAGAACCCCATCCGGAGGGCCTGCATCAGCATTGTCGAATGGAAAC
CATTTGAAATAATTATTTACTGACTATTTTTGCCAATTGTGTGGCCTTAGCGATCTATATTCCCTTTCCAGAAGATGATTCCAACGCCACCAATTCCAACCTGGAACGA
GTGGAATATCTCTTTCTCATAATTTTTACGGTGGAAGCGTTTTTAAAAGTAATCGCCTATGGACTCCTCTTTCACCCCAATGCCTACCTCCGCAACGGCTGGAACCTAC
TAGATTTTATAATTGTGGTTGTGGGGCTTTTTAGTGCAATTTTAGAACAAGCAACCAAAGCAGATGGGGCAAACGCTCTCGGAGGGAAAGGGGCCGGATTTGATG
TGAAGGCGCTGAGGGCCTTCCGCGTGCTGCGCCCCCTGCGGCTGGTGTCCGGAGTCCCAAGTCTCCAGGTGGTCCTGAATTCCATCATCAAGGCCATGGTCCCCCT
GCTGCACATCGCCCTGCTTGTGCTGTTTGTCATCATCATCTACGCCATCATCGGCTTGGAGCTCTTCATGGGGAAGATGCACAAGACCTGCTACAACCAGGAGGGCA
TAGCAGATGTTCCAGCAGAAGATGACCCTTCCCCTTGTGCGCTGGAAACGGGCCACGGGCGGCAGTGCCAGAACGGCACGGTGTGCAAGCCCGGCTGGGATGGT
CCCAAGCACGGCATCACCAACTTTGACAACTTTGCCTTCGCCATGCTCACGGTGTTCCAGTGCATCACCATGGAGGGCTGGACGGACGTGCTGTACTGGGTCAATG
ATGCCGTAGGAAGGGACTGGCCCTGGATCTATTTTGTTACACTAATCATCATAGGGTCATTTTTTGTACTTAACTTGGTTCTCGGTGTGCTTAGCGGAGAGTTTTCCA
AAGAGAGGGAGAAGGCCAAGGCCCGGGGAGATTTCCAGAAGCTGCGGGAGAAGCAGCAGCTAGAAGAGGATCTCAAAGGCTACCTGGATTGGATCACTCAGG
CCGAAGACATCGATCCTGAGAATGAGGACGAAGGCATGGATGAGGAGAAGCCCCGAAACATGAGCATGCCCACCAGTGAGACCGAGTCCGTCAACACCGAAAAC
GTGGCTGGAGGTGACATCGAGGGAGAAAACTGCGGGGCCAGGCTGGCCCACCGGATCTCCAAGTCAAAGTTCAGCCGCTACTGGCGCCGGTGGAATCGGTTCTG
CAGAAGGAAGTGCCGCGCCGCAGTCAAGTCTAATGTCTTCTACTGGCTGGTGATTTTCCTGGTGTTCCTCAACACGCTCACCATTGCCTCTGAGCACTACAACCAGC
CCAACTGGCTCACAGAAGTCCAAGACACGGCAAACAAGGCCCTGCTGGCCCTGTTCACGGCAGAGATGCTCCTGAAGATGTACAGCCTGGGCCTGCAGGCCTACT
TCGTGTCCCTCTTCAACCGCTTTGACTGCTTCGTCGTGTGTGGCGGCATCCTGGAGACCATCCTGGTGGAGACCAAGATCATGTCCCCACTGGGCATCTCCGTGCTC
AGATGCGTCCGGCTGCTGAGGATTTTCAAGATCACGAGGTACTGGAACTCCTTGAGCAACCTGGTGGCATCCTTGCTGAACTCTGTGCGCTCCATCGCCTCCCTGCT
CCTTCTCCTCTTCCTCTTCATCATCATCTTCTCCCTCCTGGGGATGCAGCTCTTTGGAGGAAAGTTCAACTTTGATGAGATGCAGTACCCATACGATGTTCCAGATTAC
GCTACCCGGAGGAGCACATTCGATAACTTCCCCCAGTCCCTCCTCACTGTGTTTCAGATCCTGACCGGGGAGGACTGGAATTCGGTGATGTATGATGGGATCATGGC
TTATGGCGGCCCCTCTTTTCCAGGGATGTTAGTCTGTATTTACTTCATCATCCTCTTCATCTGTGGAAACTATATCCTACTGAATGTGTTCTTGGCCATTGCTGTGGACA
ACCTGGCTGATGCTGAGAGCCTCACATCTGCCCAAAAGGAGGAGGAAGAGGAGAAGGAGAGAAAGAAGCTGGCCAGGACTGCCAGCCCAGAGAAGAAACAAG
AGTTGGTGGAGAAGCCGGCAGTGGGGGAATCCAAGGAGGAGAAGATTGAGCTGAAATCCATCACGGCTGACGGAGAGTCTCCACCCGCCACCAAGATCAACAT
GGATGACCTCCAGCCCAATGAAAATGAGGATAAGAGCCCCTACCCCAACCCAGAAACTACAGGAGAAGAGGATGAGGAGGAGCCAGAGATGCCTGTCGGCCCTC
GCCCACGACCACTCTCTGAGCTTCACCTTAAGGAAAAGGCAGTGCCCATGCCAGAAGCCAGCGCGTTTTTCATCTTCAGCTCTAACAACAGGTTTCGCCTCCAGTG
CCACCGCATTGTCAATGACACGATCTTCACCAACCTGATCCTCTTCTTCATTCTGCTCAGCAGCATTTCCCTGGCTGCTGAGGACCCGGTCCAGCACACCTCCTTCAG
GAACCATATTCTGTTTTATTTTGATATTGTTTTTACCACCATTTTCACCATTGAAATTGCTCTGAAGATGACTGCTTATGGGGCTTTCTTGCACAAGGGTTCTTTCTGCC
GGAACTACTTCAACATCCTGGACCTGCTGGTGGTCAGCGTGTCCCTCATCTCCTTTGGCATCCAGTCCAGTGCAATCAATGTCGTGAAGATCTTGCGAGTCCTGCGA
GTACTCAGGCCCCTGAGGGCCATCAACAGGGCCAAGGGGCTAAAGCATGTGGTTCAGTGTGTGTTTGTCGCCATCCGGACCATCGGGAACATCGTGATTGTCACCA
CCCTGCTGCAGTTCATGTTTGCCTGCATCGGGGTCCAGCTCTTCAAGGGAAAGCTGTACACCTGTTCAGACAGTTCCAAGCAGACAGAGGCGGAATGCAAGGGCA
ACTACATCACGTACAAAGACGGGGAGGTTGACCACCCCATCATCCAACCCCGCAGCTGGGAGAACAGCAAGTTTGACTTTGACAATGTTCTGGCAGCCATGATGGC
CCTCTTCACCGTCTCCACCTTCGAAGGGTGGCCAGAGCTGCTGTACCGCTCCATCGACTCCCACACGGAAGACAAGGGCCCCATCTACAACTACCGTGTGGAGATC
TCCATCTTCTTCATCATCTACATCATCATCATCGCCTTCTTCATGATGAACATCTTCGTGGGCTTCGTCATCGTCACCTTTCAGGAGCAGGGGGAGCAGGAGTACAAGA
ACTGTGAGCTGGACAAGAACCAGCGACAGTGCGTGGAATACGCCCTCAAGGCCCGGCCCCTGCGGAGGTACATCCCCAAGAACCAGCACCAGTACAAAGTGTGG
TACGTGGTCAACTCCACCTACTTCGAGTACCTGATGTTCGTCCTCATCCTGCTCAACACCATCTGCCTGGCCATGCAGCACTACGGCCAGAGCTGCCTGTTCAAAATC
GCCATGAACATCCTCAACATGCTCTTCACTGGCCTCTTCACCGTGGAGATGATCCTGAAGCTCATTGCCTTCAAACCCAAGCACTATTTCTGTGATGCATGGAATACAT
TTGACGCCTTGATTGTTGTGGGTAGCATTGTTGATATAGCAATCACCGAGGTAAACCCAGCTGAACATACCCAATGCTCTCCCTCTATGAACGCAGAGGAAAACTCCC
GCATCTCCATCACCTTCTTCCGCCTGTTCCGGGTCATGCGTCTGGTGAAGCTGCTGAGCCGTGGGGAGGGCATCCGGACGCTGCTGTGGACCTTCATCAAGTCCTTC
CAGGCCCTGCCCTATGTGGCCCTCCTGATCGTGATGCTGTTCTTCATCTACGCGGTGATCGGGATGCAGGTGTTTGGGAAAATTGCCCTGAATGATACCACAGAGAT
CAACCGGAACAACAACTTTCAGACCTTCCCCCAGGCCGTGCTGCTCCTCTTCAGGTGTGCCACCGGGGAGGCCTGGCAGGACATCATGCTGGCCTGCATGCCAGG
CAAGAAGTGTGCCCCAGAGTCCGAGCCCAGCAACAGCACGGAGGGTGAAACACCCTGTGGTAGCAGCTTTGCTGTCTTCTACTTCATCAGCTTCTACATGCTCTGT
GCCTTCCTGATCATCAACCTCTTTGTAGCTGTCATCATGGACAACTTTGACTACCTGACAAGGGACTGGTCCATCCTTGGTCCCCACCACCTGGATGAGTTTAAAAGA
ATCTGGGCAGAGTATGACCCTGAAGCCAAGGGTCGTATCAAACACCTGGATGTGGTGACCCTCCTCCGGCGGATTCAGCCGCCACTAGGTTTTGGGAAGCTGTGCC
CTCACCGCGTGGCTTGCAAACGCCTGGTCTCCATGAACATGCCTCTGAACAGCGACGGGACAGTCATGTTCAATGCCACCCTGTTTGCCCTGGTCAGGACGGCCCT
GAGGATCAAAACAGAAGGGAACCTAGAACAAGCCAATGAGGAGCTGCGGGCGATCATCAAGAAGATCTGGAAGCGGACCAGCATGAAGCTGCTGGACCAGGTG
GTGCCCCCTGCAGGTGATGATGAGGTCACCGTTGGCAAGTTCTACGCCACGTTCCTGATCCAGGAGTACTTCCGGAAGTTCAAGAAGCGCAAAGAGCAGGGCCTT
GTGGGCAAGCCCTCCCAGAGGAACGCGCTGTCTCTGCAGGCTGGCTTGCGCACACTGCATGACATCGGGCCTGAGATCCGACGGGCCATCTCTGGAGATCTCACC
GCTGAGGAGGAGCTGGACAAGGCCATGAAGGAGGCTGTGTCCGCTGCTTCTGAAGATGACATCTTCAGGAGGGCCGGTGGCCTGTTCGGCAACCACGTCAGCTA
CTACCAAAGCGACGGCCGGAGCGCCTTCCCCCAGACCTTCACCACTCAGCGCCCGCTGCACATCAACAAGGCGGGCAGCAGCCAGGGCGACACTGAGTCGCCAT
CCCACGAGAAGCTGGTGGACTCCACCTTCACCCCGAGCAGCTACTCGTCCACCGGCTCCAACGCCAACATCAACAACGCCAACAACACCGCCCTGGGTCGCCTCCC
TCGCCCCGCCGGCTACCCCAGCACGGTCAGCACTGTGGAGGGCCACGGGCCCCCCTTGTCCCCTGCCATCCGGGTGCAGGAGGTGGCGTGGAAGCTCAGCTCCA
ACAGGTGCCACTCCCGGGAGAGCCAGGCAGCCATGGCGGGTCAGGAGGAGACGTCTCAGGATGAGACCTATGAAGTGAAGATGAACCATGACACGGAGGCCTG
CAGTGAGCCCAGCCTGCTCTCCACAGAGATGCTCTCCTACCAGGATGACGAAAATCGGCAACTGACGCTCCCAGAGGAGGACAAGAGGGACATCCGGCAATCTCC
GAAGAGGGGTTTCCTCCGCTCTGCCTCACTAGGTCGAAGGGCCTCCTTCCACCTGGAATGTCTGAAGCGACAGAAGGACCGAGGGGGAGACATCTCTCAGAAGA
CAGTCCTGCCCTTGCATCTGGTTCATCATCAGGCATTGGCAGTGGCAGGCCTGAGCCCCCTCCTCCAGAGAAGCCATTCCCCTGCCTCATTCCCTAGGCCTTTTGCCA

CCCCACCAGCCACACCTGGCAGCCGAGGCTGGCCCCCACAGCCCGTCCCCACCCTGCGGCTTGAGGGGGTCGAGTCCAGTGAGAAACTCAACAGCAGCTTCCCA
TCCATCCACTGCGGCTCCTGGGCTGAGACCACCCCCGGTGGCGGGGGCAGCAGCGCCGCCCGGAGAGTCCGGCCCGTCTCCCTCATGGTGCCCAGCCAGGCTGG
GGCCCCAGGGAGGCAGTTCCACGGCAGTGCCAGCAGCCTGGTGGAAGCGGTCTTGATTTCAGAAGGACTGGGGCAGTTTGCTCAAGATCCCAAGTTCATCGAGG
TCACCACCCAGGAGCTGGCCGACGCCTGCGACATGACCATAGAGGAGATGGAGAGCGCGGCCGACAACATCCTCAGCGGGGGCGCCCCACAGAGCCCCAATGG
CGCCCTCTTACCCTTTGTGAACTGCAGGGACGCGGGGCAGGACCGAGCCGGGGGCGAAGAGGACGCGGGCTGTGTGCGCGCGCGGGGTCGACCGAGTGAGGA
GGAGCTCCAGGACAGCAGGGTCTACGTCAGCAGCCTGTAGACCCAGCTTTCTTGTACAAAGTGGTGATAATCGAATTCCGATAATCAACCTCTGGATTACAAAATTT
GTGAAAGATTGACTGGTATTCTTAACTATGTTGCTCCTTTTACGCTATGTGGATACGCTGCTTTAATGCCTTTGTATCATGCTATTGCTTCCCGTATGGCTTTCATTTTCT
CCTCCTTGTATAAATCCTGGTTGCTGTCTCTTTATGAGGAGTTGTGGCCCGTTGTCAGGCAACGTGGCGTGGTGTGCACTGTGTTTGCTGACGCAACCCCCACTGGT
TGGGGCATTGCCACCACCTGTCAGCTCCTTTCCGGGACTTTCGCTTTCCCCCTCCCTATTGCCACGGCGGAACTCATCGCCGCCTGCCTTGCCCGCTGCTGGACAGG
GGCTCGGCTGTTGGGCACTGACAATTCCGTGGTGTTGTCGGGGAAGCTGACGTCCTTTCCATGGCTGCTCGCCTGTGTTGCCACCTGGATTCTGCGCGGGACGTCC
TTCTGCTACGTCCCTTCGGCCCTCAATCCAGCGGACCTTCCTTCCCGCGGCCTGCTGCCGGCTCTGCGGCCTCTTCCGCGTCTTCGCCTTCGCCCTCAGACGAGTCG
GATCTCCCTTTGGGCCGCCTCCCCGCATCGGGAATTCCCGCGGTTCGCTTTAAGACCAATGACTTACAAGGCAGCTGTAGATCTTAGCCACTTTTTAAAAGAAAAGG
GGGGACTGGAAGGGCTAATTCACTCCCAACGAAGACAAGATCTGCTTTTTGCTTGTACTGGGTCTCTCTGGTTAGACCAGATCTGAGCCTGGGAGCTCTCTGGCTA
ACTAGGGAACCCACTGCTTAAGCCTCAATAAAGCTTGCCTTGAGTGCTTCAAGTAGTGTGTGCCCGTCTGTTGTGTGACTCTGGTAACTAGAGATCCCTCAGACCCT
TTTAGTCAGTGTGGAAAATCTCTAGCAGTAGTAGTTCATGTCATCTTATTATTCAGTATTTATAACTTGCAAAGAAATGAATATCAGAGAGTGAGAGGAACTTGTTTAT
TGCAGCTTATAATGGTTACAAATAAAGCAATAGCATCACAAATTTCACAAATAAAGCATTTTTTTCACTGCATTCTAGTTGTGGTTTGTCCAAACTCATCAATGTATCTT
ATCATGTCTGGCTCTAGCTATCCCGCCCCTAACTCCGCCCATCCCGCCCCTAACTCCGCCCAGTTCCGCCCATTCTCCGCCCCATGGCTGACTAATTTTTTTTATTTATGC
AGAGGCCGAGGCCGCCTCGGCCTCTGAGCTATTCCAGAAGTAGTGAGGAGGCTTTTTTGGAGGCCTAGGGACGTACCCAATTCGCCCTATAGTGAGTCGTATTACG
CGCGCTCACTGGCCGTCGTTTTACAACGTCGTGACTGGGAAAACCCTGGCGTTACCCAACTTAATCGCCTTGCAGCACATCCCCCTTTCGCCAGCTGGCGTAATAGC
GAAGAGGCCCGCACCGATCGCCCTTCCCAACAGTTGCGCAGCCTGAATGGCGAATGGGACGCGCCCTGTAGCGGCGCATTAAGCGCGGCGGGTGTGGTGGTTAC
GCGCAGCGTGACCGCTACACTTGCCAGCGCCCTAGCGCCCGCTCCTTTCGCTTTCTTCCCTTCCTTTCTCGCCACGTTCGCCGGCTTTCCCCGTCAAGCTCTAAATCG
GGGGCTCCCTTTAGGGTTCCGATTTAGTGCTTTACGGCACCTCGACCCCAAAAAACTTGATTAGGGTGATGGTTCACGTAGTGGGCCATCGCCCTGATAGACGGTTT
TTCGCCCTTTGACGTTGGAGTCCACGTTCTTTAATAGTGGACTCTTGTTCCAAACTGGAACAACACTCAACCCTATCTCGGTCTATTCTTTTGATTTATAAGGGATTTT
GCCGATTTCGGCCTATTGGTTAAAAAATGAGCTGATTTAACAAAAATTTAACGCGAATTTTAACAAAATATTAACGCTTACAATTTAGGTGGCACTTTTCGGGGAAAT
GTGCGCGGAACCCCTATTTGTTTATTTTTCTAAATACATTCAAATATGTATCCGCTCATGAGACAATAACCCTGATAAATGCTTCAATAATATTGAAAAAGGAAGAGTAT
GAGTATTCAACATTTCCGTGTCGCCCTTATTCCCTTTTTTGCGGCATTTTGCCTTCCTGTTTTTGCTCACCCAGAAACGCTGGTGAAAGTAAAAGATGCTGAAGATCA
GTTGGGTGCACGAGTGGGTTACATCGAACTGGATCTCAACAGCGGTAAGATCCTTGAGAGTTTTCGCCCCGAAGAACGTTTTCCAATGATGAGCACTTTTAAAGTT
CTGCTATGTGGCGCGGTATTATCCCGTATTGACGCCGGGCAAGAGCAACTCGGTCGCCGCATACACTATTCTCAGAATGACTTGGTTGAGTACTCACCAGTCACAGA
AAAGCATCTTACGGATGGCATGACAGTAAGAGAATTATGCAGTGCTGCCATAACCATGAGTGATAACACTGCGGCCAACTTACTTCTGACAACGATCGGAGGACCG
AAGGAGCTAACCGCTTTTTTGCACAACATGGGGGATCATGTAACTCGCCTTGATCGTTGGGAACCGGAGCTGAATGAAGCCATACCAAACGACGAGCGTGACACC
ACGATGCCTGTAGCAATGGCAACAACGTTGCGCAAACTATTAACTGGCGAACTACTTACTCTAGCTTCCCGGCAACAATTAATAGACTGGATGGAGGCGGATAAAGT
TGCAGGACCACTTCTGCGCTCGGCCCTTCCGGCTGGCTGGTTTATTGCTGATAAATCTGGAGCCGGTGAGCGTGGGTCTCGCGGTATCATTGCAGCACTGGGGCCA
GATGGTAAGCCCTCCCGTATCGTAGTTATCTACACGACGGGGAGTCAGGCAACTATGGATGAACGAAATAGACAGATCGCTGAGATAGGTGCCTCACTGATTAAGCA
TTGGTAACTGTCAGACCAAGTTTACTCATATATACTTTAGATTGATTTAAAACTTCATTTTTAATTTAAAAGGATCTAGGTGAAGATCCTTTTTGATAATCTCATGACCAA
AATCCCTTAACGTGAGTTTTCGTTCCACTGAGCGTCAGACCCCGTAGAAAAGATCAAAGGATCTTCTTGAGATCCTTTTTTTCTGCGCGTAATCTGCTGCTTGCAAAC
AAAAAAACCACCGCTACCAGCGGTGGTTTGTTTGCCGGATCAAGAGCTACCAACTCTTTTTCCGAAGGTAACTGGCTTCAGCAGAGCGCAGATACCAAATACTGTT
CTTCTAGTGTAGCCGTAGTTAGGCCACCACTTCAAGAACTCTGTAGCACCGCCTACATACCTCGCTCTGCTAATCCTGTTACCAGTGGCTGCTGCCAGTGGCGATAAG
TCGTGTCTTACCGGGTTGGACTCAAGACGATAGTTACCGGATAAGGCGCAGCGGTCGGGCTGAACGGGGGGTTCGTGCACACAGCCCAGCTTGGAGCGAACGAC
CTACACCGAACTGAGATACCTACAGCGTGAGCTATGAGAAAGCGCCACGCTTCCCGAAGAGAGAAAGGCGGACAGGTATCCGGTAAGCGGCAGGGTCGGAACAG
GAGAGCGCACGAGGGAGCTTCCAGGGGGAAACGCCTGGTATCTTTATAGTCCTGTCGGGTTTCGCCACCTCTGACTTGAGCGTCGATTTTTGTGATGCTCGTCAGG
GGGGCGGAGCCTATGGAAAAACGCCAGCAACGCGGCCTTTTTACGGTTCCTGGCCTTTTGCTGGCCTTTTGCTCACATGTTCTTTCCTGCGTTATCCCCTGATTCTG
TGGATAACCGTATTACCGCCTTTGAGTGAGCTGATACCGCTCGCCGCAGCCGAACGACCGAGCGCAGCGAGTCAGTGAGCGAGGAAGCGGAAGAGCGCCCAATA
CGCAAACCGCCTCTCCCCGCGCGTTGGCCGATTCATTAATGCAGCTGGCACGACAGGTTTCCCGACTGGAAAGCGGGCAGTGAGCGCAACGCAATTAATGTGAGT
TAGCTCACTCATTAGGCACCCCAGGCTTTACACTTTATGCTTCCGGCTCGTATGTTGTGTGGAATTGTGAGCGGATAACAATTTCACACAGGAAACAGCTATGACCAT
GATTACGCCAAGCGCGCAATTAACCCTCACTAAAGGGAACAAAAGCTGGAGCTGCAAGCTT

Supplementary Notes

```
---# This code normalizes the mean gray values to the baseline, removes low respondents & technical
noise
---# and calculates the residual calcium

# Assuming you have the 'readxl' package installed
library(readxl)

# Specify the path to your Excel file
excel_file <- "/path/to/folder/sample1.xlsx"

# Read the data from the Excel sheet
data <- read_excel(excel_file)

# Calculate the average baseline value of each cell
cell_averages <- apply(data[1:30, ], 2, mean)

# Normalize the data based on the average value of each cell
data <- sweep(data, 2, cell_averages, FUN = "/")

# Calculate the mean of the baseline period
baseline_means <- apply(data[1:30, ], 2, mean)

# Calculate the threshold for response amplitude
thresholds_min <- baseline_means * 1
thresholds_max <- baseline_means * 20

# Identify cells with response amplitudes within the threshold range
selected_cells <- apply(data[31:nrow(data), ], 2, function(cell) {
  response_amplitude <- max(cell, na.rm = TRUE) - baseline_means
  response_amplitude >= thresholds_min & response_amplitude <= thresholds_max
})

# Convert the data to numeric format
data <- as.data.frame(apply(data, 2, as.numeric))

# Filter out cells that have response amplitudes less than the threshold
filtered_data <- data[, apply(selected_cells, 2, any), drop = FALSE]

# Save filtered and normalized data
write.csv(filtered_data, "/path/to/folder/filt.csv")

# Calculating the residual calcium

# Assuming your data is stored in a variable called 'data'
# where each column represents a cell and each row represents a timepoint

# Calculate the number of timepoints
num_timepoints <- nrow(filtered_data)

# Initialize vectors to store the results
peaks <- vector("numeric", ncol(filtered_data))
```

```
values_A <- vector("numeric", ncol(filtered_data))
values_B <- vector("numeric", ncol(filtered_data))
values_C <- vector("numeric", ncol(filtered_data))
ups <- vector("numeric", ncol(filtered_data))
downs <- vector("numeric", ncol(filtered_data))
residuals <- vector("numeric", ncol(filtered_data))

# Loop over each cell
for (i in 1:ncol(filtered_data)) {
  # Find the index of the peak value within frame 10 and 40
  peak_index <- which.max(filtered_data[10:40, i]) + 10

  # Check if peak_index is within valid range
  if (peak_index >= 21 && peak_index <= num_timepoints - 200) {
    # Extract values A, B, and C
    value_A <- filtered_data[peak_index - 20, i]
    value_B <- filtered_data[peak_index, i]
    value_C <- filtered_data[peak_index + 200, i]

    # Calculate 'up' and 'down'
    up <- value_C - value_A
    down <- value_B - value_A

    # Calculate 'residual'
    residual <- up / down

    # Store the results
    peaks[i] <- peak_index
    values_A[i] <- value_A
    values_B[i] <- value_B
    values_C[i] <- value_C
    ups[i] <- up
    downs[i] <- down
    residuals[i] <- residual
  }
}

# Create a data frame with the results
result <- data.frame(Cell = 1:ncol(filtered_data),
                     PeakIndex = peaks,
                     ValueA = values_A,
                     ValueB = values_B,
                     ValueC = values_C,
                     Up = ups,
                     Down = downs,
                     Residual = residuals)

# Filter out cells with NA residuals or residuals smaller than -5 or larger than 5
filtered_result <- result[!is.na(result$Residual) & result$Residual > -5 & result$Residual < 5 &
result$Residual != 0, ]

print(filtered_result)
```

```
write.csv(filtered_result, "/path/to/folder/residuals.csv")
```
