# Supplementary material for: Antisense oligonucleotide therapeutic approach for Timothy syndrome
Source: Nature. 2024 Apr 24;628(8009):818–25. doi: 10.1038/s41586-024-07310-6 (PMC11043036; doi:10.1038/s41586-024-07310-6)
Supplement: Supplementary file 2 — Reporting Summary [file 41586_2024_7310_MOESM2_ESM.pdf]

nature portfolio

Corresponding author(s): Sergiu P. Pasca

Last updated by author(s): 2024/01/28

Reporting Summary

Nature Portfolio wishes to improve the reproducibility of the work that we publish. This form provides structure for consistency and transparency in reporting. For further information on Nature Portfolio policies, see our Editorial Policies and the Editorial Policy Checklist.

Statistics

For all statistical analyses, confirm that the following items are present in the figure legend, table legend, main text, or Methods section.

n/a	Confirmed	
☐	☒	The exact sample size (*n*) for each experimental group/condition, given as a discrete number and unit of measurement
☐	☒	A statement on whether measurements were taken from distinct samples or whether the same sample was measured repeatedly
☐	☒	The statistical test(s) used AND whether they are one- or two-sided<br>*Only common tests should be described solely by name; describe more complex techniques in the Methods section.*
☐	☒	A description of all covariates tested
☐	☒	A description of any assumptions or corrections, such as tests of normality and adjustment for multiple comparisons
☐	☒	A full description of the statistical parameters including central tendency (e.g. means) or other basic estimates (e.g. regression coefficient) AND variation (e.g. standard deviation) or associated estimates of uncertainty (e.g. confidence intervals)
☐	☒	For null hypothesis testing, the test statistic (e.g. *F*, *t*, *r*) with confidence intervals, effect sizes, degrees of freedom and *P* value noted<br>*Give P values as exact values whenever suitable.*
☒	☐	For Bayesian analysis, information on the choice of priors and Markov chain Monte Carlo settings
☒	☐	For hierarchical and complex designs, identification of the appropriate level for tests and full reporting of outcomes
☒	☐	Estimates of effect sizes (e.g. Cohen's *d*, Pearson's *r*), indicating how they were calculated

Our web collection on statistics for biologists contains articles on many of the points above.

Software and code

Policy information about availability of computer code

Data collection	QuantStudio 6/7 software V7 (real-time qPCR);<br>Image Lab (DNA gel electrophoresis, V6.1.0 build 7);<br>Clampex (pClamp) 11.1 (Patch clamp recordings);<br>Paravision 6.0.1 (MR imaging);<br>Las-X (Leica) was used for acquiring interneuron migration, immunohistochemistry images and Calbryte 520 imaging; FACS Diva was used for acquiring flow cytometry;<br>U-COR Odyssey Clx imaging system for Western blot.
Data analysis	Image Lab V6.l.0 build 7;<br>GraphPad Prism 9.3.1 {350);<br>FlowJo 10.7.1;<br>MATLAB R2019b and R2022b;<br>ImageJ V2.1.0/1.53q;<br>Clampfit (pClamp) 10.6.2.2;<br>Geneious prime 2020.2.3:<br>Image Studio V5.2.5.

For manuscripts utilizing custom algorithms or software that are central to the research but not yet described in published literature, software must be made available to editors and reviewers. We strongly encourage code deposition in a community repository (e.g. GitHub). See the Nature Portfolio guidelines for submitting code & software for further information.

Data

Policy information about availability of data

All manuscripts must include a data availability statement. This statement should provide the following information, where applicable:

- Accession codes, unique identifiers, or web links for publicly available datasets
- A description of any restrictions on data availability
- For clinical datasets or third party data, please ensure that the statement adheres to our policy

Details on source data and code are provided with this manuscript.

Research involving human participants, their data, or biological material

Policy information about studies with human participants or human data. See also policy information about sex, gender (identity/presentation), and sexual orientation and race, ethnicity and racism.

Reporting on sex and gender	Sample 1: Male (18 years old)<br>Sample 2: Female (3 years old)
Reporting on race, ethnicity, or other socially relevant groupings	None
Population characteristics	The two postnatal human tissue samples (age 3- and 18-year-old) were obtained from resection of frontal lobe cortex (middle frontal gyrus), and were reported in Revah et al., Nature (2022).
Recruitment	Postnatal samples were collected as part of surgeries for treating medically refractory epilepsy.
Ethics oversight	Human cerebral cortical tissue was obtained with informed consent under a protocol approved by the Stanford University Institutional Review Board.

Note that full information on the approval of the study protocol must also be provided in the manuscript.

Field-specific reporting

Please select the one below that is the best fit for your research. If you are not sure, read the appropriate sections before making your selection.

☒ Life sciences ☐ Behavioural & social sciences ☐ Ecological, evolutionary & environmental sciences

For a reference copy of the document with all sections, see nature.com/documents/nr-reporting-summary-flat.pdf

Life sciences study design

All studies must disclose on these points even when the disclosure is negative.

Sample size	Sample sizes were estimated empirically, based on previous studies (Birey et al., Nature 2017; Marton et al., Nature Neuroscience; Pasca et al, Nature Medicine 2019; Miura et al., Nature Biotechnology 2020; Khan et al, Nature Medicine 2020; Birey et al, Cell Stem Cell 2022)
Data exclusions	To extract migration parameters, a cell had to undergo at least one saltation. Immobile cells were not included in the analysis.<br>For GCaMP imaging analysis, a genetically encoded indicator (rather than a ratio-metric dye) was used; cells displaying imaging artifacts or cells with very low or very high rises (20x) from the baseline mean were not included in the analysis. Extreme residual calcium values (lower than -5 or higher than +5) were also excluded.
Replication	Data shown from representative experiments were repeated with similar results in at least 3 independent experiments, unless otherwise indicated by sample size.
Randomization	Organoids and assembloids were randomly picked for each assay and distributed for ASO treatment or ASO scramble control.
Blinding	Golgi staining and dendrite tracing were blinded. Blinding was not relevant or used for other experiments.

Reporting for specific materials, systems and methods

We require information from authors about some types of materials, experimental systems and methods used in many studies. Here, indicate whether each material, system or method listed is relevant to your study. If you are not sure if a list item applies to your research, read the appropriate section before selecting a response.

Materials & experimental systems

n/a	Involved in the study
☐	☒ Antibodies
☐	☒ Eukaryotic cell lines
☒	☐ Palaeontology and archaeology
☐	☒ Animals and other organisms
☒	☐ Clinical data
☒	☐ Dual use research of concern
☒	☐ Plants

Methods

n/a	Involved in the study
☒	☐ ChIP-seq
☐	☒ Flow cytometry
☐	☒ MRI-based neuroimaging

Antibodies

Antibodies used	anti-CTIP2 (Rat, 1:300, Abeam, ab18465; [25B6]) ; anti-SATB2 (Mouse, 1:300, Abeam, Cat. No. ab51502; [GR323578-6]) ; anti-human nuclear antigen-(HNA) (Mouse, 1:200, Abeam, ab191181; [235-1] ;anti-Human CD90-PE (Mouse, 1:100, BD Biosciences, 555596; [93151721) ; anti-CaVl.2 (CACNA1C) Antibody (rabbit, 1:1000, Alomone labs, ACC-003; [ACC003AN7102]); anti-rat Alexa 488(Donkey, 1:1000, Thermofisher Scientific; Cat. No. A-21208); anti-mouse Alexa 568 (Donkey, 1:1000, Thermofisher Scientific; Cat. No. A10037); anti-rabbit 568 (1:1000, Thermofisher Scientific; Cat. No. A10042)
Validation	CTIP2, see Andersen et. al Cell 2020, validated in immunohistochemistry staining (IHC) in human cortical organoids.<br>SATB2 and HNA, see Revah et. al Nature 2022, validated in IHC in transplanted human cortical organoids.<br>CD90-PE, same clone as the non-conjugated CD90 validated in Trevino et. al Science 2020 used for immunopanning of human neurons. This is also validated by manufacturer by flow cytometry.<br>CaV1.2, validated in KO experiments by the manufacturer, and in human cardiac tissue (Crossman, D.J. et al. (2011) PLoS ONE 6, e17901.)

Eukaryotic cell lines

Policy information about cell lines and Sex and Gender in Research

Cell line source(s)	2242-1 Stanford IRB; 8119-1 UCLA IRB; 1208-2 UCLA IRB;<br>9862-2 Stanford IRB; 7643-6 Stanford IRB; 8303-4 Stanford IRB<br>KOLF2.1 and TS-KOLF2.l from the the Jackson laboratory<br>CW30293 from CIRM<br>Lenti-X 293T from Takara Bio
Authentication	hiPS cell lines were assessed for genomic integrity by SNP microarray (lllumina genome-wide SNP microarray GSAMD-24v2-0)
Mycoplasma contamination	Cell lines were regularly tested for Mycoplasma contamination and tested negative.
Commonly misidentified lines (See ICLAC register)	The HEK 293T cell line was used.

Animals and other research organisms

Policy information about studies involving animals; ARRIVE guidelines recommended for reporting animal research, and Sex and Gender in Research

Laboratory animals	FOXNl-/- male and female rats between P3 and 9 months of age were used in this study.
Wild animals	No wild animals were used in this study.
Reporting on sex	Both male and female
Field-collected samples	No field samples were collected in this study.
Ethics oversight	Approval for transplantation of hCSO into rats was obtained from the Stanford Laboratory Animal Care (APLAC) Research Compliance Office.

Note that full information on the approval of the study protocol must also be provided in the manuscript.

Plants

Seed stocks	*Report on the source of all seed stocks or other plant material used. If applicable, state the seed stock centre and catalogue number. If plant specimens were collected from the field, describe the collection location, date and sampling procedures.*
Novel plant genotypes	*Describe the methods by which all novel plant genotypes were produced. This includes those generated by transgenic approaches, gene editing, chemical/radiation-based mutagenesis and hybridization. For transgenic lines, describe the transformation method, the number of independent lines analyzed and the generation upon which experiments were performed. For gene-edited lines, describe the editor used, the endogenous sequence targeted for editing, the targeting guide RNA sequence (if applicable) and how the editor was applied.*
Authentication	*Describe any authentication procedures for each seed stock used or novel genotype generated. Describe any experiments used to assess the effect of a mutation and, where applicable, how potential secondary effects (e.g. second site T-DNA insertions, mosiacism, off-target gene editing) were examined.*

Flow Cytometry

Plots

Confirm that:

☒ The axis labels state the marker and fluorochrome used (e.g. CD4-FITC).

☒ The axis scales are clearly visible. Include numbers along axes only for bottom left plot of group (a 'group' is an analysis of identical markers).

☒ All plots are contour plots with outliers or pseudocolor plots.

☒ A numerical value for number of cells or percentage (with statistics) is provided.

Methodology

Sample preparation	Dissociated single cells from hCO
Instrument	BD Aria
Software	Samples were acquired byBD FACS Diva and analyzed by Flowjo.
Cell population abundance	No sorting was performed.
Gating strategy	hCOs not treated with Cy5-ASO were used as a negative control for setting the gate.

☒ Tick this box to confirm that a figure exemplifying the gating strategy is provided in the Supplementary Information.

Magnetic resonance imaging

Experimental design

Design type	Anatomical brain structures in anesthetized rats
Design specifications	N/A
Behavioral performance measures	N/A

Acquisition

Imaging type(s)	Structural
Field strength	7T
Sequence & imaging parameters	Axial 2D Turbo-RARE (TR=2500 ms, TE=33 ms, 2 averages) 16 slice acquisitions were performed with 0.6-0.8 mm slice thickness, with 256x256 samples. Signal was received with a 2 cm inner-diameter quadrature transmit-receive volume radio frequency coil (Rapid MR international LLC)
Area of acquisition	Whole rat brain

Diffusion MRI ☐ Used ☒ Not used

Preprocessing

Preprocessing software	N/A
Normalization	N/A
Normalization template	N/A
Noise and artifact removal	N/A
Volume censoring	N/A

Statistical modeling & inference

Model type and settings	N/A
Effect(s) tested	N/A

Specify type of analysis: ☒ Whole brain ☐ ROI-based ☐ Both

Statistic type for inference (See Eklund et al. 2016)	N/A
Correction	N/A

Models & analysis

n/a	Involved in the study
☒	☐ Functional and/or effective connectivity
☒	☐ Graph analysis
☒	☐ Multivariate modeling or predictive analysis
